# Conductive Covalent Organic Frameworks as Chemiresistive Sensor Arrays for the Detection and Differentiation of Gasotransmitters

**DOI:** 10.1021/jacs.5c11454

**Published:** 2025-11-13

**Authors:** Georganna Benedetto, Robert M. Stolz, Zheng Meng, Joseph Y. M. Chan, Elissa O. Shehayeb, Colin T. Morrell, Gbenga Fabusola, Nikolaus Elsaesser, Cory M. Simon, Katherine A. Mirica

**Affiliations:** † Department of Chemistry, 3728 Dartmouth College, Hanover, New Hampshire 03755, United States; ‡ School of Chemical, Biological, and Environmental Engineering, 2694 Oregon State University, Corvallis, Oregon 97331, United States; § Department of Mathematics, Oregon State University, Corvallis, Oregon 97331, United States

## Abstract

This paper describes a chemiresistive sensor array using four structurally analogous, but chemically distinct, conductive covalent organic frameworks (COFs) (M-COF-DC-8, M = Fe, Co, Ni, and Cu) capable of detecting and differentiating four important gaseous analytes: nitric oxide (NO), carbon monoxide (CO), hydrogen sulfide (H_2_S), and ammonia (NH_3_). The COFs were synthesized from the condensation of 2,3,9,10,16,17,23,24-octaamino-metallophthalocyanine precursors with pyrenetetraone linkers resulting in chemically robust and electrically conductive materials. Chemiresistive sensing experiments, together with machine learning to parse the response pattern of the sensor array, show that the M-COF-DC-8 (M = Fe, Co, Ni, Cu) materials can detect and differentiate this suite of oxidizing and reducing gases at parts-per-million concentrations, with theoretical limits of detection (LOD) in the parts-per-billion range in dry N_2_. Importantly, the COF array containing M-COF-DC-8 (M = Co, Ni, Cu) retains its ability to detect and differentiate these analytes in air and humidity under low power consumption. Spectroscopic investigations reveal that the synthetic control over the identity of the metallophthalocyanine core efficiently tunes material–analyte interactions and, therefore, emergent device performance. The use of highly tunable COFs as the active material in sensor arrays enables low-power, sensitive, and real-time gas detection with future applications in healthcare and personal protection.

## Graphical abstract

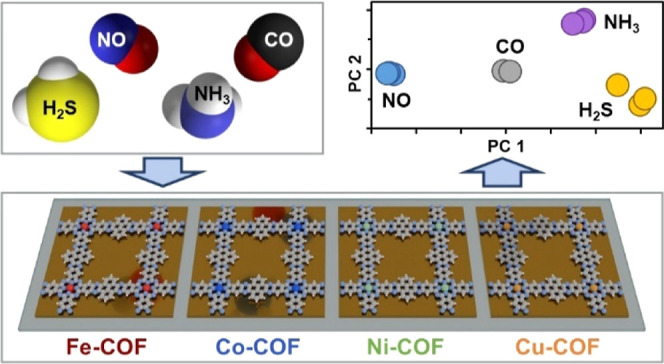

## Introduction

Quantification of gaseous analytes such as gasotransmitters—NO, CO, and H_2_S—as well as NH_3_ in the parts-per million (ppm) regime via sensing devices enables continuous environmental monitoring,^1^ long-term medical monitoring,^2^ and closed system studies such as bioreactor gas monitoring.^3,4^ These analytes are both potent toxins via inhalation^5,6^ and endogenous biological regulators for cardiovascular, neurological, pulmonary, gastrointestinal, and immune functions.^7–9^ However, due to the low concentrations of these gases and the presence of interferants, methods enabling sensitive and selective analysis at relevant physiological concentrations are limited.^10^ The current approaches to identification and differentiation of these gases in high-risk exposure environments require a multitude of semiconductor metal oxide (MOS) sensors with limitations in room-temperature operation.^11^ Clinical settings currently rely on the use of gas-chromatography–mass spectrometry (GC–MS) techniques that limit continuous and widespread monitoring due to large instruments and the requirement of technicians to operate them.^12^ Selective gas differentiation to enable understanding of mechanism, function, and concentration profiles of gasotransmitters and NH_3_ remains inaccessible with current techniques. The exogenous and endogenous importance of these key analytes necessitates the development of novel materials to enable the detection and differentiation of gasotransmitters and NH_3_ at biologically relevant LODs (parts per billion (ppb)), while limiting the diminishing effect of humidity on analyte identification and reducing the power consumption necessary for device function.

Chemiresistive sensors possess features necessary to meet the challenges associated with gas quantification due to ease of miniaturization,^13^ minimal power requirements,^14,15^ and aggregation of responses from an array of chemiresistive active materials to enable transduction and differentiation of sensing events from a complex mixture of analytes.^14,16–22^ While various 1D (conductive polymers,^23^ and carbon nanotubes (CNTs)) and 2D (graphene-based materials,^24–26^ black phosphorus,^27^ transition metal dichalcogenides (TMDC),^28–30^ and MOSs^31–34^ ) active materials are capable of detection of certain gasotransmitters and NH_3_, these materials fail to simultaneously differentiate multiple gases, limiting widespread applicability.^14^ Chemiresistive arrays overcome this challenge where strategic choice of a set of active materials provides a wide range of discernible responses to gasotransmitters.^35^ Arrays consisting of CNTs,^36^ and MOSs,^37–39^ have been able to differentiate some gasotransmitters, but not all three with sub-ppm theoretical LODs.^13^ These materials are limited by intrinsic chemical sensitivity and selectivity of their conductive components, and they require sensitizers, such as transition metal complexes or metal nanoparticles, to provide useful levels of sensitivity and selectivity.^33^ Additionally, MOS sensors require post-synthetic modification and high operating temperatures, resulting in high power consumption.^14,40^ While operative, methods that combine separate components to impart electrical and chemical sensitivity lack simplicity, efficiency, chemical stability, and enhanced performance. These benefits can be achieved by integrating conductive pathways and chemical recognition motifs within a single molecularly precise framework, such as conductive metal−organic frameworks (MOFs) and covalent organic frameworks (COFs).^20,41–45^ Framework materials are capable of low ppm detection of NO, CO, H_2_S, and NH_3_ ^44,46^ and, in some cases, limited analyte differentiation,^43,47–49^ but the technology has not yet demonstrated the ability to detect and differentiate all three gasotransmitters and NH_3_. Moreover, examination of the effect of humidity on analyte selectivity in air remains underexplored for framework-based arrays. Further strategic material development and systematic sensing studies are needed to deconvolute material–analyte interactions to design a chemiresistive sensor capable of sensitive and selective gas differentiation.

This paper describes the use of four structurally analogous, conductive COFs with embedded metallophthalocyanine (MPc) molecules in a chemiresistive array for the detection and differentiation of three gasotransmitters (NO, CO, H_2_S) and NH_3_. The sensor array comprising a set of M-COF-DC-8 materials (M = Fe, Co, Ni, and Cu) provided partially cross-reactive material–analyte interactions between NO, CO, H_2_S, and NH_3_. Ni-COF-DC-8 previously showed chemiresistive sensing capabilities for detection of NO, H_2_S, and NH_3_.^50^ Although spectroscopic techniques supported charge-transfer mechanisms as a key feature of the reported chemiresistive sensitivity,^50^ the impact of variable metal identity in the MPc core on resulting material sensing capabilities remains unexplored in a COF array. The COF array in this work achieved (i) detection of the analytes with theoretical LODs well below permissible exposure limits, LOD_CO_ = 860 ppb, LOD_NO_ = 0.4 ppb, LOD_H_2_S_ = 28 ppb, LOD_NH_3__ = 73 ppb (based on 30 min exposure experiments), (ii) differentiation of all analytes and different ppm-level concentrations of them via principal component analysis (PCA), and (iii) differentiation of all analytes at 80 ppm despite the presence of potential interferants in air and humidified environments. To the best of our knowledge, this work constitutes the first report of a chemiresistive array for the chemical sensing and differentiation of three gasotransmitters (NO, CO, H_2_S) and NH_3_. This work also reports two novel COFs (Fe-COF-DC-8 and Cu-COF-DC-8). The use of diffuse reflectance infrared Fourier transform spectroscopy (DRIFTS), X-ray photoelectron spectroscopy (XPS), and electron paramagnetic resonance spectroscopy (EPR) enabled the identification of structural features of the M-COF-DC-8 materials responsible for binding and reacting to gas analytes. Specifically, the identity of the metal in the MPc unit influenced the strength of Lewis acid sites of the framework materials and their ability to bind diatomic gases, such as NO and CO. Additionally, we identified reactions between reducing gases, such as H_2_S and NH_3_, with defect sites (edge sites and incomplete annulation) as a possible pathway in the chemiresistive response. Our study provides fundamental insight into the role of the MPc unit in chemical sensing and demonstrates an important link between the selectivity of material–analyte interactions imparted by MPc portions of the frameworks and the selectivity of chemiresistive sensing. Density functional theory (DFT) suggests chemiresistive transduction pathways could be an emergent property arising from material–analyte interactions at the MPc centers and the intrinsic electronic structure of the material. Our investigation offers an approach to sensor array design that leverages the atomically precise tunability enabled by reticular synthesis, and the chemical stability, sensitivity, and conductivity of pyrazine-linked COFs.

## Experimental Design

### Gases as Analytical Targets and Spectroscopic Probes

We chose to focus this study on the detection and differentiation of NO, CO, H_2_S, and NH_3_ because these analytes are important endogenously as biological regulators^51,52^ and exogenously as hazardous pollutants.^53^ Deviations in the concentrations of these gases in exhaled breath from those found in healthy individuals (NO: 10−50 ppb, CO: 0−6 ppm, H_2_S: 0−1.3 ppm, and NH_3_: 0.5−2 ppm) could serve as general indicators of disease.^54^ Based on their respective physiological functions and points of generation, the presence of these endogenous small molecules could indicate the possibility of conditions including inflammatory respiratory disorders, general inflammatory diseases, lung and airway tissue diseases, and kidney failure.^7,54^ From exogenous sources, these analytes possess time-weighted average (TWA) 8 h permissible exposure limits of 25, 50, 10, and 50 ppm for NO, CO, H_2_S, and NH_3_, respectively, as defined by the Occupational Safety and Health Administration (OSHA).^55–58^ From a public health and safety perspective, it is important to accurately detect and differentiate these analytes.

From a spectroscopic perspective, NO, CO, H_2_S, and NH_3_ possess distinct elemental compositions, symmetries, and electronic structures, providing unique features to probe the surface of novel materials. Upon binding to a surface, the unique features of these molecules can change as their symmetry, oxidation state, and distribution of electron density within bonds are altered by chemisorption on a surface.^59^ Identifying hosting surfaces can provide insights into host-site chemistry, such as types of acidity and basicity (e.g., Lewis acid/base or Brønsted acid/base), and locations of host sites on heterogeneous surfaces (e.g., organic ligand, metal center, or defect sites).^60^

### Molecular Design of Conductive COFs for Sensing Gasotransmitters and NH_3_

#### Metallophthalocyanines

The molecular design for sensing gases in this work employs compounds based on the Fe, Co, Ni, and Cu derivatives of the phthalocyanine macrocycle embedded within a conductive 2D porous scaffold. The first-row transition metals (TMs) were chosen to represent a sliding scale of d-orbital population, electronegativity, and reduction potentials. These features influence the surface chemistry during interactions with gases, potentially enabling the materials to differentiate among analytes.

MPc monomers have served as active components in the chemiresistive detection of NO, CO, and H_2_S, and NH_3_.^61–65^ Fundamental investigations have been performed by Trogler and others that provide a strong precedent for the ability of MPc molecules to act as transducing materials for chemical detection.^66–70^ Despite their ability to transduce gas adsorption events, their application in chemiresistive devices required driving voltages as high as 10 V, due to their low conductivity, incurring high power usage, resistive heating, and low device stability. Building on this precedent to amplify the utility of MPc-based sensing materials, our group demonstrated that integrating MPc units into 2D COFs^50^ and MOFs^48,49^ results in ppb–ppm range sensing capabilities at driving voltages as low as 0.1 V, with good selectivity for a range of gas analytes.

#### Framework Material Scaffold

Our molecular engineering strategy in this study employs 4-fold connectivity and rigid planar aromatic structure of four transition MPcs, resulting in four isostructural COFs exhibiting a monoclinic lattice system and strong interlayer interactions (Figure 1). The conjugated 2D conductive M-COF-DC-8 materials possess three unique characteristics enabling them to serve as active materials in a chemiresistive array. First, MPc units confer tunable characteristics to the framework materials, depending on the identity of the metal center, and serve as possible host sites for analyte interaction. Second, conjugated COFs combine sufficient conductivity, enabling low-power electronic transduction mechanisms, with a precisely ordered crystalline structure.^46,71–73^ These highly ordered, crystalline structures are beneficial for studies aimed at understanding structure–property relationships.^74–77^ Third, COFs, especially those with pyrazine linkages, exhibit increased chemical and thermal stability compared to MOFs while retaining a metal center capable of interaction with gaseous analytes.^74–78^

**Figure 1:**
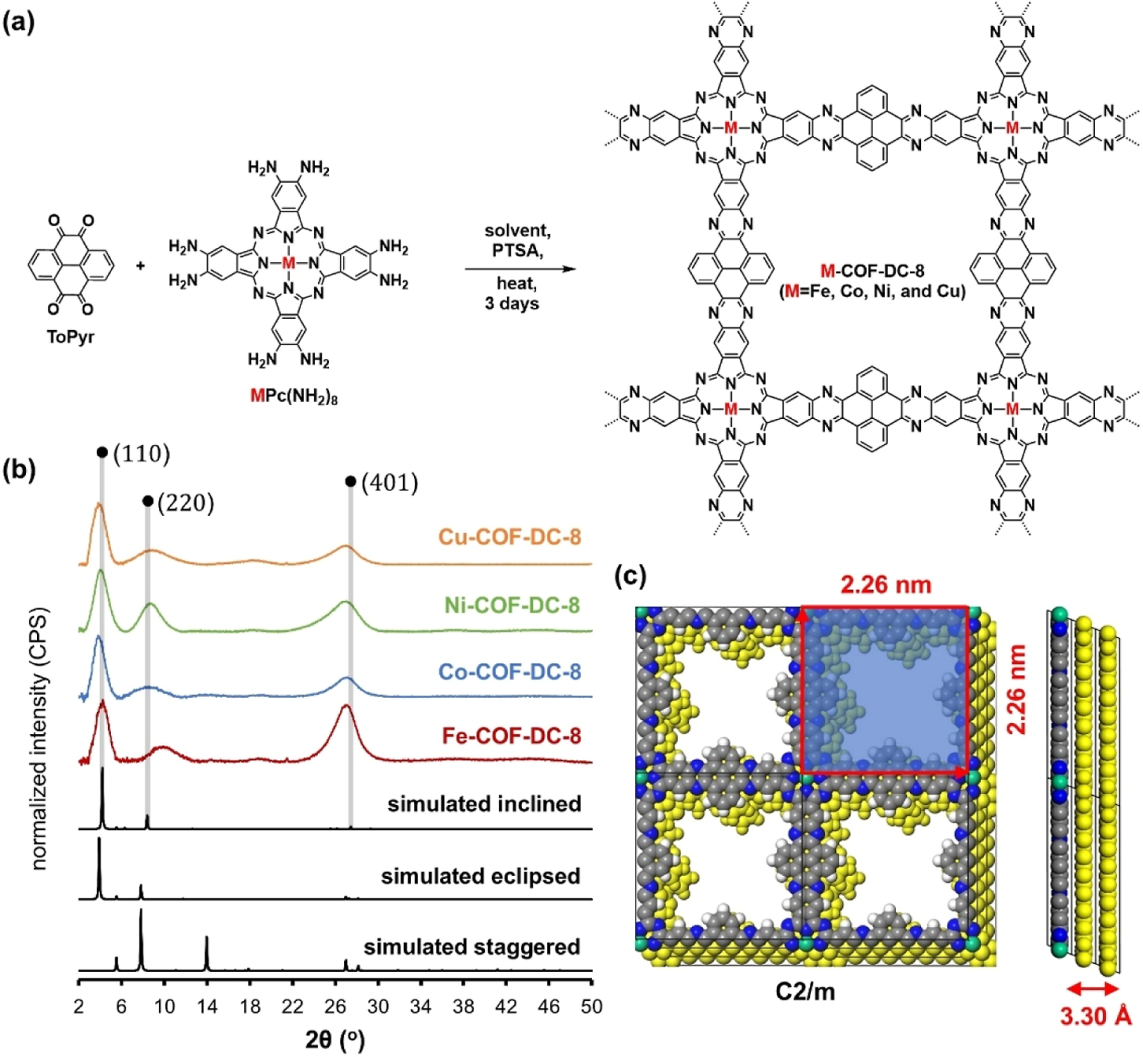
(a) The synthetic scheme used to achieve 4 distinct derivatives of M-COF-DC-8 (M = Fe, Co, Ni, and Cu). (b) Characterization of isostructural M-COF-DC-8 (M = Fe, Co, Ni, and Cu) by PXRD. Diffraction planes of the simulated inclined packing pattern are listed as shaded lines. The simulated inclined, eclipsed, and staggered diffraction patterns are presented at the bottom of the plot for comparison. (c) Simulated crystal structure of the COFs consisting of 2D sheets with inclined stacking patterns. Colored spheres are atomic positions: gray = carbon, blue = nitrogen, green = metal, white = hydrogen, yellow = additional unit cell layers. Distances between atoms and 2D layers are shown in red.

## Results and Discussion

### Synthesis of M-COF-DC-8

Four derivatives of M-COF-DC-8 (M = Fe, Co, Ni, and Cu) were synthesized via the condensation of 2,3,9,10,16,17,23,24-octaamino-metallophthalocyanine (MPc­(NH_2_)_8_) with pyrenetetraone as seen in Figure 1a. MPc­(NH_2_)_8_ (M = Fe, Co, Ni, and Cu) were synthesized via approaches detailed in Section II of the Supporting Information. Condensation of the two precursors in a 1:2 ratio (MPc­(NH_2_)_8_:pyrenetetraone) formed fully conjugated, aromatic layers linked by robust pyrazine linkages. Due to the different metal center identities, MPc­(NH_2_)_8_ precursors can vary in solubility and dimerization constants, which affects MPc stacking,^79^ thus necessitating individualized synthetic conditions for each COF. To optimize material crystallinity and quality, 3.5 M *p*-toluenesulfonic acid (PTSA) was used as an acid promoter, and the specific synthetic procedure for each COF varied with respect to the identity of the organic solvent, and the reaction temperature (150–185 °C). Additionally, the free-base precursor (NiPc­(NH_2_)_8_) was used for the synthesis of Ni-COF-DC-8. However, for the synthesis of M-COF-DC-8 (M = Fe, Co, and Cu), the MPc­(NH_2_)_8_ precursor was washed with hydrochloric acid (HCl) to generate an HCl salt form of the precursor prior to COF synthesis. The salt form of the precursor reduced the reactivity of the free amines and prevented precursor degradation prior to COF condensation. The resulting dark green to black COF powders were isolated in 31–98% yield. For more information on COF synthesis and synthetic optimization trials, see Section III of the Supporting Information.

### Structure and Characterization of Crystallinity and Morphology

The formation of stacked 2D crystalline frameworks was supported by unit cell parameters obtained from an analysis of powder X-ray diffraction (PXRD) experiments. Experimental PXRD patterns generated from the four COF powders were compared to simulated spectra generated from crystal structures featuring inclined, eclipsed, and staggered packing patterns (Figures 1b,c, S4 and S5).^50^ While the COFs exhibited some variation in the peak positions, indicative of slight changes in crystal structure (see Table S6 in Section III of the Supporting Information), the experimental diffraction peaks generally aligned with the simulated inclined stacking pattern, which features peaks at 2θ = 4.2°, 8.4°, and 27.4°, corresponding to the (110), (220), and (401) diffraction planes, respectively (Figure 1c). The experimental diffraction peaks between 3.80–4.06° 2θ were close but slightly lower than the simulated inclined (110) diffraction plane generated from the proposed COF pore diameter of 2.26 nm (Figure 1b). These experimental peaks align slightly better with the simulated eclipsed (100) diffraction plane at 3.90°. However, due to the position of the second experimental peak positioned at 8.52–9.90° for the four COFs, which align more closely with the inclined stacking pattern as opposed to the eclipsed, we anticipate some degree of inclination in COF layers. The broad experimental diffraction peaks at 27.02–27.16° are well approximated by the experimental (401) peak, indicating an anticipated interlayer distance of 3.3 Å (Figure 1b). Despite the strong diffraction patterns from all four materials, the slight variation in peak positions for all three prominent lattice planes suggests variations in 2D layer stacking and structural disorder in the COFs, a known phenomenon noted in the field.^78,80^ However, due to the broadness of diffraction peaks, likely caused by small crystallite size and variation in packing patterns in a single COF sample, more precise details regarding the specific angles of COF layer inclination could not be fully resolved. Further work is required to control the irreversible COF formation reaction to produce larger, more ordered crystals for robust structural analysis.

Scanning electron microscopy (SEM) images showed that the nanocrystalline powders were composed of faceted particles ranging from submicron to micron scale (Figure 2a–d). Samples were prepared for transmission electron microscopy (TEM) analysis by sonication in water overnight, followed by drop casting the suspension onto the TEM grid and allowing the grid to dry in air. TEM analysis determined that the dimensions of the framework structure were consistent with those obtained by PXRD. We observed that pore–pore distances (Figure 2e–h) were consistent with previously reported Ni-COF-DC-8 and with PXRD analysis (Figure 1b).^50^ More electron microscopy images detailing material morphology and particle size at different imaging sites are seen in Figures S12–S14.

**Figure 2:**
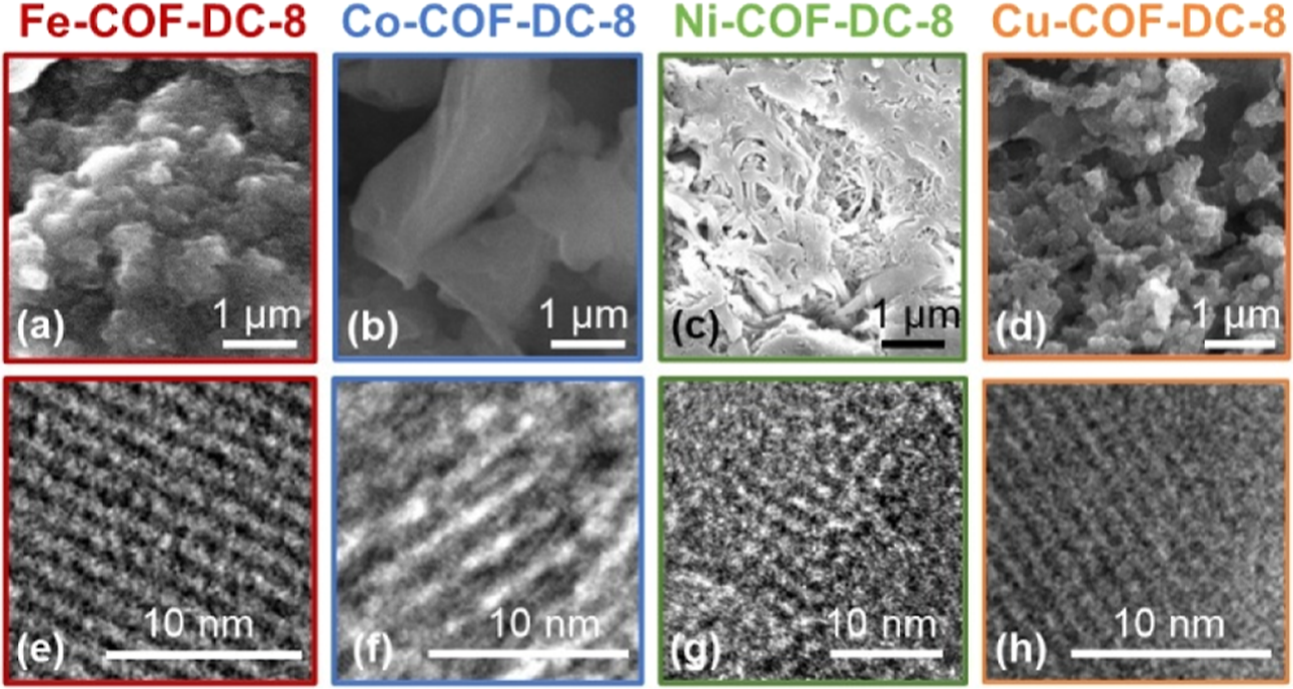
Electron microscopy of M-COF-DC-8 (M = Fe, Co, Ni, and Cu) framework materials. Scanning electron microscopy images of (a) Fe-, (b) Co-, (c) Ni-, and (d) Cu-COF-DC-8. Transmission electron microscopy images of (e) Fe-, (f) Co-, (g) Ni-, and (h) Cu-COF-DC-8.

### Chemical Characterization of Bond Formation and the Component Redox States

IR characterization supported the successful formation of pyrazine linkages, as evidenced by the appearance of absorbance bands around 1518, 1431, and 1351 cm^–1^ (see Figure S11).^50,81^ Additionally, the broad peaks around 3300–3400 cm^–1^ suggest the presence of either unreacted primary amines or the presence of water in the COF.^81^ Elemental analysis confirmed that the materials contained M (M = Fe, Co, Ni, or Cu), C, N, and H near abundances anticipated by the theoretical stoichiometry (Section IV of the Supporting Information). XPS analysis further identified that the elemental composition of all four COFs consisted of C, N, and the M anticipated for each material (Section V of the Supporting Information). Additionally, all four COFs showed strong O 1s emission lines which we assigned to adsorbed water and defect sites (e.g., edge sites with ketones due to incomplete annulation).

EPR spectroscopy of the pristine COFs also suggested a +2 oxidation state of the transition metal components in the Co-, Ni-, and Cu-analogues of M-COF-DC-8 (Figure S16). At room temperature (RT), Fe-COF-DC-8 displayed no observable absorbance in the X-band across a wide field (600–5000 G, *g* = 9.5–1.5). At cryogenic temperatures (4.2 K), Fe-COF-DC-8 showed three prominent absorbance features (*g* = 5.807, 4.269, and 1.997, Figure S16), as well as a complex set of line shapes at g = 2.5–2.3. The absorbance lines at high g-values (*g* = 5.807 and 4.269) were assigned to high spin (HS) Fe^3+^ (S = 5/2),^82,83^ while the complex set of line shapes with an absorbance line observed at *g* = 2.500 was assigned to the low-spin (LS) Fe^3+^ (S = 1/2) or an admixed intermediate spin Fe^3+^ having a possible S = 3/2 component. The remaining absorbance at *g* = 1.997 was assigned to a radical with little spin–orbit coupling possibly centered in a ligand orbital or O_2_ adsorbed to the framework (Figure S16).^84,85^

Similarly, the room temperature EPR spectrum of Co-COF-DC-8 displayed a small absorbance at the *g* = 1.955 position (Figure S16). No metal-centered radicals were observed at this temperature. Once the sample was lowered to cryogenic conditions (4.2 K), we observed a new set of absorbance lines at *g* = 4.301, 2.254, and 1.975. The weak absorbance at *g* = 4.301 and the intense absorbance at 2.254 were assigned to an LS Co^2+^ (S = 1/2) having *g*_∥_ and *g*_⊥_ components, respectively. We assigned the feature centered at 1.975 exhibiting hyperfine interactions, to interactions with the Co^2+^ nucleus due to the multiplicity of the line (8 features).^86^

The EPR spectrum of the Ni-COF-DC-8 matched our previous report.^50^ At room temperature, we observed a similar absorbance location and FWHM compared to the RT spectrum of Co-COF-DC-8, but with a much stronger intensity. This absorbance, with an absorbance maximum at *g* = 1.997, was assigned to a ligand-centered radical potentially part of the Pc ring or adsorbed O_2_ species. At 4.2 K, the spectrum of Ni-COF-DC-8 showed similar absorbance features, but with a higher intensity (Figure S16).

Cu-COF-DC-8 displayed an absorption line at *g* = 2.048, which was shifted to higher *g* values from those observed for the organic radical contained in Ni-COF-DC-8 and Co-COF-DC-8 analogs (Figure S16). Additionally, the absorption line was broad and symmetric, which suggested the radical was centered in a Cu^2+^ (S = 1/2) frontier orbital.^87^

### Characterization of Porosity

Nitrogen (N_2_) adsorption isotherms were recorded for all four COFs at 77 K (Figure S17). The void space in the measurement vessels was measured by backfilling with He at 295 K. Brunauer–Emmett–Teller (BET) analysis in the region *p*/*p*° 0.2–0.3 revealed that the M-COF-DC-8 materials displayed varying surface areas: Fe-COF-DC-8: 120 m^2^/g, Co-COF-DC-8: 89 m^2^/g, Ni-COF-DC-8: 217 m^2^/g, Cu-COF-DC-8: 11 m^2^/g. The values for the Co- and Ni-based COFs were lower than those previously reported in the literature.^50,81^ We attribute these discrepancies to differences in activation procedure and smaller crystallite size.^88^ Considering the predicted isostructural nature of the COFs, the comparatively low surface area obtained for Cu-COF-DC-8 indicates either (i) diminished crystallinity of the material compared to the other three COFs, (ii) variation in 2D layer stacking resulting in inaccessible pores,^89^ and/or (iii) incomplete material activation and pore collapse.^90^ Despite various synthetic and structural reasons for this low surface area, Cu-COF-DC-8 exhibited a micropore area of 2 m^2^/g indicating microporous nature of the material. A more in-depth discussion of the specific surface area (SSA) results is provided in Section X of the Supporting Information.

### Characterization of Conductivity

The conductivity of the M-COF-DC-8 materials was assessed using the four-point probe method on a pressed pellet (under ∼1000 psi) of polycrystalline powder with no further activation. The conductivity of the pellets under ambient air for M-COF-DC-8 (M = Fe, Co, Ni, and Cu) was 1.1 × 10^–4^ S/cm, 3.8 × 10^–5^ S/cm, 9.1 × 10^–7^ S/cm, and 2.2 × 10^–6^ S/cm, respectively. The conductivities of the Co- and Ni-derivatives were reported previously (CoPc-PDQ: 3.7 × 10^–5^ S/cm,^81^ Ni-COF-DC-8: 2.5 × 10^–5^ S/cm).^50^ The conductivity values measured for Co-COF-DC-8 and Ni-COF-DC-8 were similar to those found in other reports on these materials. It is likely that the difference in material conductivity arises from the effect of (1) different metal centers and (2) varying degrees of material crystallinity. Additionally, it is important to note that the measurement of bulk conductivity from a pressed pellet may be influenced by extrinsic aspects of the material being measured (e.g., particle size, surface chemistry, activation method, the pressure used to press the pellet, and the relative humidity in the environment during testing, etc.).

### Computational Investigation of Electronic Structure

DFT calculations were used to model the electronic band structure of the materials using the CASTEP program applying the GGA-PBE exchange-correlation functional (Section XV of the Supporting Information).^91,92^ All four COFs presented highly curved Dirac bands overlapping indirectly to give a semimetal band structure. The band structures were observed to be highly dependent on the spin state of the materials. Our computational assessment of the band structure of the materials was in agreement with previous reports.^50^ Symmetry lowering antiferromagnetic states for the metal centers (HS Fe^2+^, Co^2+^, and Cu^2+^) were of *I*4/*mmm* symmetry when antiferromagnetically coupled, and *P*4/*mmm* when they were ferromagnetically aligned (Section XV of the Supporting Information). The band structure of the antiferromagnetically coupled materials had a gapless direct band structure and less curvature in their Dirac bands.

### Chemiresistivity of M-COF-DC-8

In our first report on this class of materials, Ni-COF-DC-8 showed excellent sensitivity toward small reactive gases such as NO, NO_2_, H_2_S and NH_3_ at ppb−ppm concentrations.^50^ Multifunctional properties, such as an embedded metal center, intrinsic conductivity or conductance in a thin film, and chemically robust skeleton, are features common to all the herein reported M-COF-DC-8 frameworks, suggesting a tunable capacity for chemical sensing. To determine the chemiresistive response of the M-COF-DC-8 materials toward gas-phase analytes, we tested the materials against oxidizing (NO) and reducing (CO, H_2_S, NH_3_) gases. Each M-COF-DC-8 was sonicated into a suspension in H_2_O (1.5 mg/mL) and 10 μL was applied to gold interdigitated microelectrodes (5 μm gap) by drop casting and air drying. Devices prepared in this way had a yield of 75% across all materials (*N* = 64). Typical devices had resistances on the order of 0.1–5.0 MΩ. To determine sensing performance metrics, such as reversibility, rate of response, the magnitude of response, and limit of detection, we performed 30 min gas exposure experiments to at least three devices fabricated from the same COF synthetic batch for each analyte and material combination (Figure 3). Errors represent the standard deviation from the mean calculated from 3−5 devices used in one exposure experiment. For specifics on the chemiresistive sensing procedures, see Section XII of the Supporting Information.

**Figure 3:**
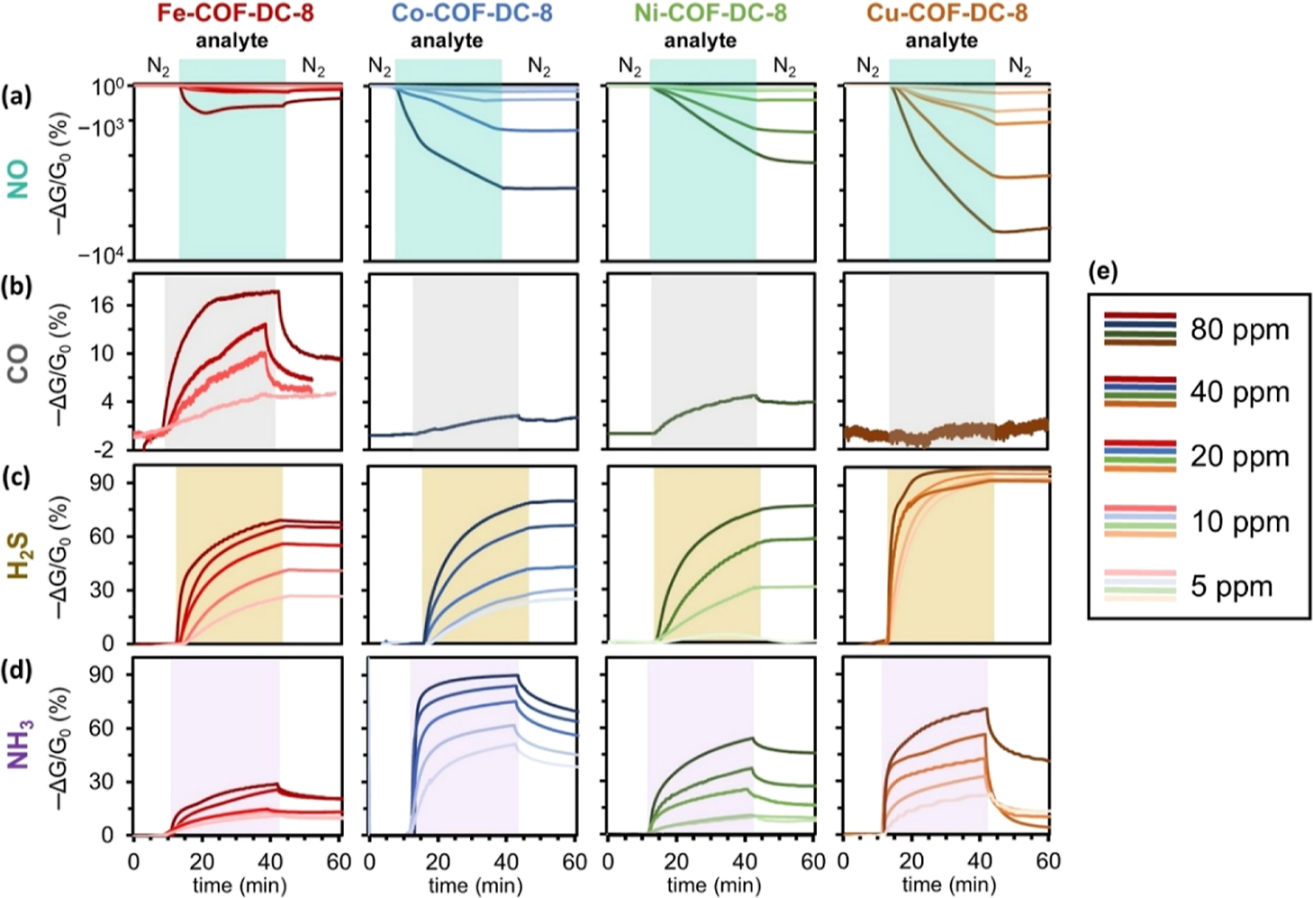
Chemiresistive sensing responses of M-COF-DC-8 (a−d: M = Fe, Co, Ni, and Cu) to analytes at concentrations of 80, 40, 20, 10, and 5 ppm of (top−bottom) NO, CO, H_2_S, and NH_3_. Responses are measured in negative normalized conductance (−Δ*G*/*G*_0_). Exposures of the devices to analyte (shaded regions) were maintained for 30 min before the flow was returned to 100% N_2_. Measurements were made using an applied potential of 1.0 V and a measurement time interval of 0.5 s. (e) Key depicting analyte concentration corresponding to sensing trace color.

In response to 80 ppm of NO, the largest normalized response was observed for Cu-COF-DC-8 at −7235 ± 662%, followed by Co-COF-DC-8 ( −5665 ± 144%), and Ni-COF-DC-8 (−3097 ± 971%) (Figure 3a). Our previous report discovered that Ni-COF-DC-8 was able to give high responses toward NO (Ni-COF-DC-8: −3939 ± 317%).^50^ While these three materials showed high sensitivity and no reversibility in response to NO, Fe-COF-DC-8, by comparison, had a normalized response of −1644 ± 142% with a nonmonotonic response curve and a semi-reversible response allowing partial recovery of device response from NO exposure (Figure 3a). The negative-going normalized response for these materials toward NO reinforced the p-type nature of these semiconductive materials.

Chemiresistive sensing experiments demonstrated that Fe-COF-DC-8 was able to detect 80 ppm of CO with a normalized response of 17 ± 1% after 30 min (Figure 3b). This study constitutes the first report of a COF-based sensor for the detection of CO via chemiresistive sensing. However, CO sensing capability was highly dependent on Fe-COF-DC-8 crystallinity and synthetic reproducibility as evidenced in Figure S23. M-COF-DC-8 (M = Co, Ni, and Cu) showed similarly unreliable but much weaker responses to 80 ppm of CO in batch-dependent studies (Figures 3b, and S20). The response of Fe-COF-DC-8 coupled with the smaller response of the Co-, Ni-, and Cu-analogues toward CO provided an opportunity for a cross-reactive sensor array for differentiating CO from other gasotransmitters and NH_3_.

In response to 80 ppm of H_2_S for 30 min, Cu-COF-DC-8 had the highest response at 99 ± 1%, followed by Co-COF-DC-8, Ni-COF-DC-8, and Fe-COF-DC-8 with responses of 77 ± 3%, 76 ± 1%, and 70 ± 1%, respectively (Figure 3c). All materials exhibited dosimetric responses to H_2_S as evidence by the irreversible chemiresistive responses. In response to 80 ppm of NH_3_ for 30 min, Co-COF-DC-8 yielded the highest −Δ*G*/*G*_0_ of 88 ± 3%, followed by Cu-COF-DC-8 (78 ± 6%), Ni-COF-DC-8 (25 ± 10%), and Fe-COF-DC-8 (29 ± 1%) (Figure 3d). Although Co-COF-DC-8, Ni-COF-DC-8, and Cu-COF-DC-8 showed partial reversibility in response to NH_3_ (Figure 3d). The positive normalized response observed for H_2_S and NH_3_, corresponded to an increased resistance of the materials. The increased resistance in response to reducing gases likely stems the p-type semiconductor COF interacting with a reducing gases, thereby diminishing the concentration of free charge carriers.^93^ In general, the relationship between the concentration of all four analyte and different device metrics (response at saturation, area under the curve, and initial rate of response (RoR)) followed a linear relationship as seen in Figure S18, thereby indicating concentration dependence.

Most of the material–analyte pairings did not reach full saturation during the 30 min analyte exposure and, as previously mentioned, the devices exhibited either semi-reversible or dosimetric responses following analyte exposure. These features likely stem from kinetics and availability of active sites for analyte interaction, as well as the irreversibility of the chemisorptive material–analyte interactions, respectively. Literature precedent suggests that in some cases device regeneration and recyclability is possible following heat treatment or washing, depending on the nature of chemisorption.^50^

### Determination of Limits of Detection

We adopted the convention of defining theoretical LOD as the intersection of the linear fit of the 30 min percent response (−Δ*G*/*G*_0_) at different concentrations of analytes with the value 3 × (root-mean-square (RMS) noise). The RMS noise was determined by fitting a fifth-order polynomial to the baseline of the sensing traces in a region before gas exposure. Calculated LOD values are presented in Table 1. As an array, the LOD was taken as the lowest observed theoretical LOD across all M-COF-DC-8 materials for each analyte. Cu-COF-DC-8 presented the lowest LOD against NO at 0.4 ppb and H_2_S at 28 ppb. The array LOD for CO, provided by Fe-COF-DC-8, was 860 ppb, and the lowest LOD for NH_3_ was 75 ppb using Ni-COF-DC-8. These values are either on par or exceed some of the best reported LODs obtained with MPc-based framework materials such as MPc-based MOFs and COFs.^46,48–50^ Additionally, the marked increase in chemiresistive response to CO enabled via Fe-COF-DC-8 compared to the negligible responses of the other M-COF-DC-8 (M = Co, Ni, and Cu) materials indicates that the FePc analog plays the dominant, if not sole role in CO detection. While previous MPc-based framework materials have achieved ppb-level CO LODs by relying on Cu-bis­(dioxolene) linkages for analyte–material interaction,^48^ no framework has leveraged the advantageous FePc analogue, which demonstrates improved CO adsorption compared to the other MPcs.^48^

**Table 1:** Theoretical Limits of Detection at 30 min of Exposure for Each M-COF-DC-8 in the Array against Each Gas

	LOD (ppb)			
M-COF-DC-8	NO	CO	H_2_S	NH_3_
Fe-COF-DC-8	7	860	46	297
Co-COF-DC-8	1.4	n/a	63	79
Ni-COF-DC-8	3.6	n/a	160	75
Cu-COF-DC-8	0.4	n/a	28	106

### Analysis of Sensing Kinetics

In chemiresistive sensing experiments, the RoR within the first 1–2 min of exposure can be used to investigate reaction rates and as an attribute in multivariate selectivity analysis. The initial RoR is found by fitting a line to the initial response data and plotted with the initial RoR for other concentrations to provide a linear response regime (Figure S18c). While Fe-COF-DC-8 was the least responsive to NO in terms of conductance, the material exhibited the largest initial RoR due to the large increase in RoR at 80 ppm of NO exposure compared to lower NO concentrations. While the RoR was not directly proportional to NO concentrations for Fe-COF-DC-8, it was for the rest of the COFs. The initial RoR of Ni-COF-DC-8 to NO, H_2_S, and NH_3_ was generally the lowest while the initial RoR of Cu-COF-DC-8 was the largest for H_2_S. On an array of M-COF-DC-8 materials, these RoR are further differentiated by material providing a potential method of discriminating between gases and concentrations.

### Spectroscopic Investigation of Host–Guest Interactions and Sensing Mechanism

To determine the nature of host–guest interactions between analyte and M-COF-DC-8 we applied XPS, DRIFTS, and EPR to characterize key features of the chemiresistive mechanism, such as guest adsorption location and transduction mechanism. Our method of determining these features relied on a strategy of using analyte gases as probes of surface chemistry. For the DRIFTS experiments, dosing gas of 1% analyte (10,000 ppm) in dry N_2_ was used to ensure sufficient signal-to-noise and maximize surface adsorption to analyze analyte–material interactions. This high concentration facilitates detection of subtle chemical interaction. However, it is important to note that sensing performance was evaluated at a maximum of 80 ppm analyte. Despite this disparity between analyte concentrations used for sensing and spectroscopic investigation, we anticipate similar modes of interaction within this concentration range.

#### NO Gas as a Probe

Exposure of COFs to 1% NO (balance N_2_) using *in situ* DRIFTS revealed material-specific responses characterized by three distinct spectroscopic features. First, we observed strong absorption bands appeared at 1694 cm^–1^ on Fe-COF-DC-8 and at 1715 cm^–1^ on Co-COF-DC-8, but no similar bands in the region appeared for the other two analogs, Ni-COF-DC-8 and Cu-COF-DC-8 (Figure 4a). We also noticed potential weak bands for Ni-COF-DC-8 that overlapped with the R-branch of gas-phase NO (Figures 4a, and S27). We assigned the strong bands at 1694 cm^–1^ and 1715 cm^–1^ to *v*(NO) modes of NO bound to FePc and CoPc centers, respectively.^94^ Additionally, we observed vibrational modes of NO on these metal centers at wavenumbers below that of free NO,^95^ suggesting a large degree of back bonding between NO and the metal to generate a NO^δ−^ species. This band was most prominent for Cu-COF-DC-8 and least prominent for Fe-COF-DC-8 compared to all materials suggesting a correlation of this band with the sensing response of the materials (Figure 4a). The identity of this band was ambiguous because few molecular motifs exhibit vibrational frequencies in this region. We, therefore, tentatively assigned this band to one of four potential interactions: (1) the non-specific adsorption of NO to the frameworks, (2) perturbations to the aromatic backbone of the framework causing new C–H deformation modes in this region, (3) formation of electronic trap sites upon adsorption of NO at defect sites (unreacted ketones), (4) distortions to the framework stemming from NO_2_ contamination, or (5) related to band structure changes in the material. Third, we observed that, with time, all the frameworks exhibited a growing band at 1432 and 1356 cm^–1^ which we assigned to adsorbed nitrate and nitro groups, respectively (Figure S27). Because of the high reactivity of NO with O_2_, which we anticipated to be adsorbed to the framework surface,^96^ we could not definitively identify the source of these products from impurities in the gas stream or production at the COF surface.^97^

**Figure 4:**
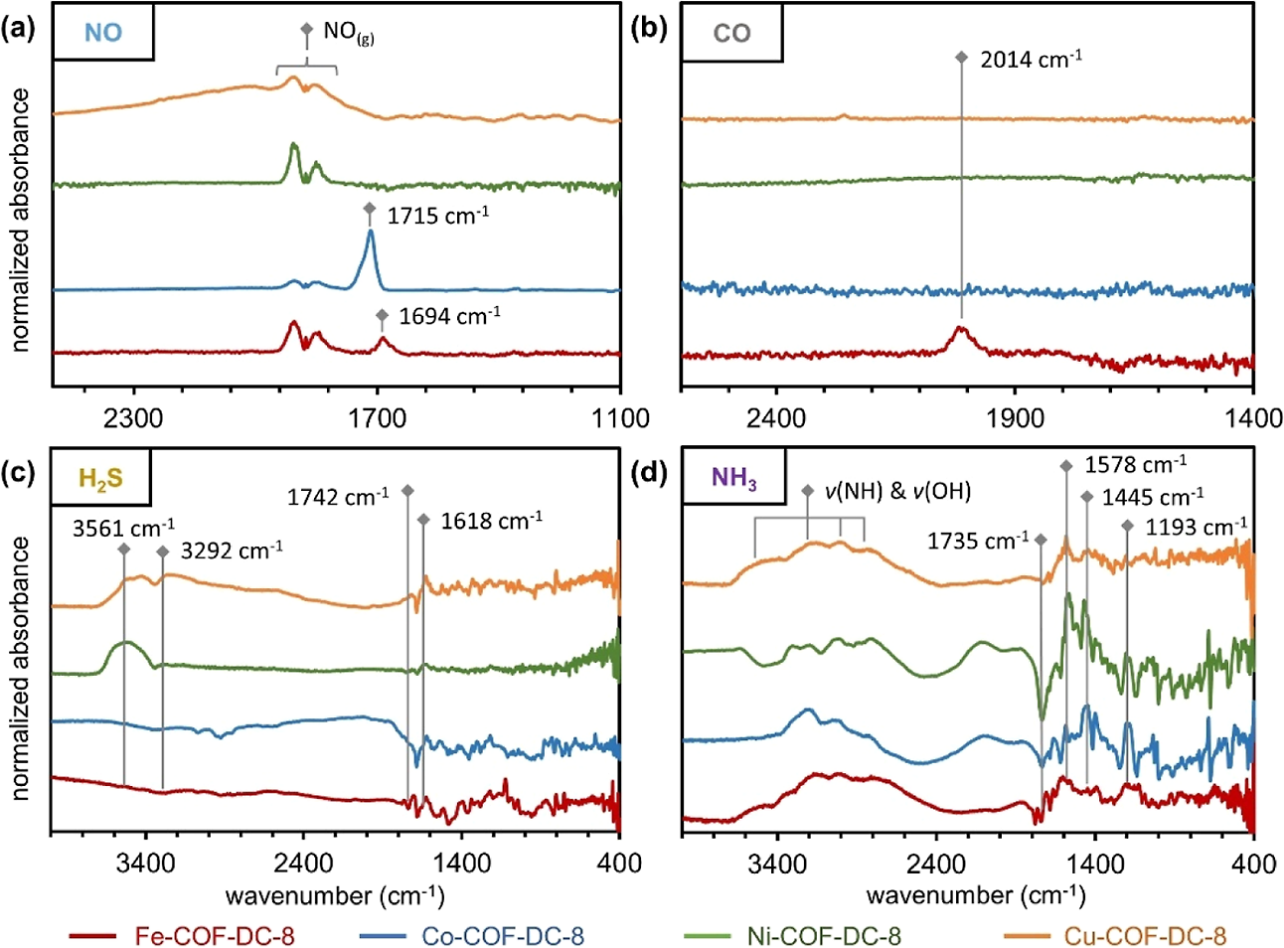
DRIFTS difference spectra of the four M-COF-DC-8 COFs after exposure 1% of analyte (a) NO, (b) CO, (c) H_2_S, and (d) NH_3_. Materials were exposed to NO for 10 min. Materials were exposed to the rest of the analytes for 20 min followed by 4 min of purging with N_2_.

XPS spectroscopy of the NO-dosed samples investigated the irreversible changes to the COFs. The spectra remained largely unchanged apart from the regions corresponding to the N 1s and O 1s emission. Variations in the O 1S species content (related to unreacted ketones or adsorbed solvent),^50^ indicated that defect sites played key roles in analyte interaction and the materials exhibited changes in bound solvent upon exposure. All M-COF-DC-8 materials investigated displayed a new component at high binding energies in the N 1s region (405–407 eV, Figures S32–S35). This new species was assigned to NO_3_^–^, which was corroborated by DRIFTS.

EPR analysis of M-COF-DC-8 samples after dosing showed discrete changes from the pristine spectra (Figure S48). For all ERP experiments, materials were dosed with 1% analyte for 20 min followed by brief degassing for 10 s to remove any free analyte from the headspace. On Fe-COF-DC-8, EPR revealed that NO caused the formation of a new strong and broad absorbance feature observed at *g* = 2.030, suggesting a LS system with some spin–orbit coupling. We attributed this new feature to a low-spin metal-centered radical. The unchanged nature of the HS Fe^3+^ features implied that the new absorbance most likely resulted from a LS Fe^2+^ to LS Fe^3+^ upon adsorption of NO.^98^ The strong shift of the *v*(NO) corroborated a strong donating interaction from Fe to NO.^95^ For Ni- and Cu-COF-DC-8, the addition of NO caused a strong increase in the features corresponding to organic radicals. We attributed this increase to the adsorption of radical-containing NO. Additionally, Co-COF-DC-8 showed a strong feature at *g* = 4.400 suggesting the formation of CoPc-NO complexes (Figure S48).^99^

#### CO as a Probe

Like NO, CO is capable of back bonding and σ-donating interactions in coordination complexes. DRIFTS spectra revealed that Fe-COF-DC-8 was the only COF in the series that showed spectral features corresponding to vibrational modes of adsorbed CO. All the materials showed weak perturbations to features in the region below 1800 cm^–1^. These features were reminiscent of features observed in other framework systems upon exposure to gas analytes, although they were too weak here to reliably assign to particular lattice modes. We note that the less robust sensing response of the other COF batches (batch 1 in Figure S26) to CO might indicate that adsorption interactions between the Ni-, Co- and Cu-COF-DC-8 materials and CO might be too weak to observe in our DRIFTS experiments beyond these small perturbations. For Fe-COF-DC-8, we observed a new asymmetric absorption band with a maximum at 2014 cm^–1^ under an atmosphere of 1% CO in N_2_ (Figure 4b).^95^ The new band was persistent after CO was removed from the headspace, but slowly decreased with extended purging. Closer inspection of the absorption band early in the exposure process revealed two distinct adsorption features centered at 2009 cm^–1^ and 1998 cm^–1^ (Figure 4b). The location of these new features was consistent with CO bonding to a FePc molecule with strong back bonding contributions.^100^ We assigned the absorbance bands to CO bonding with exposed FePc units of the framework. XPS and EPR spectroscopy did not reveal any changes in the frameworks after exposure to CO. We expect this observation is due to the weak adsorption energy of CO (XPS: Figures S36–S39, EPR: Figure S48). We noted that Fe-COF-DC-8 was the only material we examined that exhibited spectroscopic evidence for CO binding to the FePc site on the COFs and the only material that exhibited a response to CO in chemiresistive experiments. These two observations lead us to conclude that the incorporation of a FePc host site into the COF structure led to the selective ability to bind and transduce interactions with CO.

#### H_2_S as a Probe

Upon exposure to 1% H_2_S in N_2_, DRIFTS experiments revealed that the COFs displayed four spectral features that were generally conserved across the set of M-COF-DC-8 frameworks. The first of these features was a positive-going bands between 3670–3340 cm^–1^ and another positive going band at 3292 cm^–1^ (Figures 4c, and S29). The bands were most pronounced for the Ni- and Cu-derivatives while Fe-COF-DC-8 showed weaker and broader bands in this region and the Co-derivative exhibited a broad band. Second, we observed a set of broad and weak bands between 2981–1972 cm^–1^ (Figure S29). Third, we observed a pair of sharp negative-going bands between 1756–1737 cm^–1^ and 1685–1675 cm^–1^ for all the M-COF-DC-8s (Figure S29). Fourth, a broad positive-going band was observed at 1637 cm^–1^ (Figure 4c). The bands between 3670–3340 cm^–1^ and the band at 1637 cm^–1^ were assigned to the *v*(OH) and δ­(HOH) modes of water, respectively.^101^ The higher energy modes at 3292 cm^–1^ could also indicate the presence of alcohol groups formed during the reduction of ketone defect sites. The positive nature of these bands suggested water and alcohol groups were potential products of the reaction of H_2_S with the COFs. The pair of negative-going bands between 1756–1737 cm^–1^ and 1685–1675 cm^–1^ corresponded with similarly shaped bands in the pristine COFs ascribed to carbonyl groups at the terminal edges of the COF crystallites.^50^ We ascribe these negative-going bands to the reduction of carbonyl groups. Finally, the broad bands observed between 2981–1972 cm^–1^ could correspond to the formation of N–H species upon reduction of the pyrazine linkage, or disruption of aromatic deformation modes within the framework (Figure S29).^102^

DRIFTS suggested that the COFs reacted with H_2_S, including redox properties involving framework host-sites. While providing useful information as to the reactivity of the framework toward reducing gases, DRIFTS was unable to identify specific sulfur-containing products. XPS was able to identify sulfur-containing species by the appearance of new lines in the region corresponding to the S 2p transition. All of the COFs exhibited two new primary lines in this region at 163.8 and 165.1 eV corresponding to the S 2p_3/2_ emission lines for sulfur, sulfide, or thiols, and sulfoxides or sulfinyls, respectively.^103,104^ These spectroscopic results suggest that the chemiresistive response toward H_2_S may arise from interactions of the reducing gas with defect sites of the COFs. EPR revealed that H_2_S exposure caused a decrease in the absorbance intensity of the absorbance bands for all four M-COF-DC-8 materials (Figure S48). These types of radicals were broken down into two types. First, metal-centered radicals such as HS/LS Fe^3+^, LS Co^2+^, and Cu^2+^ are all oxidation state-dependent, and reduction (or conversion to an LS state) would lead to a reduced X-band absorbance. Second, radicals centered at *g* = 1.997 (Co-, Ni-COF-DC-8) were not metal-centered but instead most likely a result of either adsorbed species (O_2_) or defects (semiquinone from pyrene dicarbonyl edge groups) systemic to the 2D framework.^105,106^ Because H_2_S interacted with these materials as a reducing gas, we anticipated that H_2_S could be reducing O_2_ adsorbed to the framework (to generate water) or could be oxidizing radicals at defect sites.^107^

#### NH_3_ as a Probe

With DRIFTS, NH_3_ showed specific vibrational modes corresponding to NH_3_ adsorbing to BAS or LAS of the frameworks (Figures 4d, and S30).^60^ Our first observation was that Fe-COF-DC-8 and Co-COF-DC-8 showed the strongest Lewis acidity as evidenced by sharp bands with maxima at 1580 and 1603 cm^–1^, respectively, corresponding to the asymmetrical vibration (δ_asym_) of adsorbed NH_3_.^108^ Cu-COF-DC-8 showed the next strongest Lewis acidity with a band maximum at 1578 cm^–1^ followed by Ni-COF-DC-8 (maximum: 1571 cm^–1^). The trend of Lewis acidity (FePc, CoPc, CuPc, NiPc) was born out in the symmetrical vibration (δ_sym_) of adsorbed NH_3_ as well (Figure S30). All four COFs also showed weak Lewis acidity evidenced by bands observed in the region of ∼1200 cm^–1^ (Figure S30). Additional stronger bands we assigned to strong Brønsted acidity were observed for Cu-, Ni-, and Co-COF-DC-8 from 1442–1475 cm^–1^ (Figure S30).^108^ XPS (Figures S44–S47) and EPR (Figure S48) results did not reveal any additional information about interactions between NH_3_ and the frameworks. Like H_2_S, NH_3_ interactions with the four COFs also caused strong negative-going bands in the region assigned to carbonyl groups at edge sites. We propose that these interactions were vital to the observed chemiresistive response. The pattern of Lewis acidity that analysis of NH_3_ bands revealed (strongest-to-weakest: FePc, CoPc, CuPc, NiPc), matched previously reported adsorption energies for NH_3_ on MPc molecules.^109^

Taken together, spectroscopic evidence revealed three specific insights into the host–guest chemistry of materials. First, the identity of the metal center controlled the ability of the frameworks to strongly adsorb NO and CO, two gases that interact at metal sites as both σ-donors and π-acceptors. Fe-COF-DC-8 and Co-COF-DC-8 both showed strong interactions with NO while the other derivatives (Ni-COF-DC-8 and Cu-COF-DC-8) did not. Of the materials examined, Fe-COF-DC-8 was also the only COF capable of binding CO. This trend suggests that the Fe centers within Fe-COF-DC-8 are both the strongest σ-accepting metal and the strongest π-donating metal followed by Co-COF-DC-8. Second, spectroscopic evidence from DRIFTS studies with Lewis basic NH_3_ pointed to a sliding scale of Lewis acidity for the COFs. The strongest acidity was observed for Fe-COF-DC-8 followed by Co-COF-DC-8, Cu-COF-DC-8, and Ni-COF-DC-8. Although variation in COF morphology and particle size may contribute to different sensing responses,^88^ the consistent metal-dependent spectral changes observed across the COF samples indicate that metal–gas interactions are a major contributing factor in sensor performance. A systematic study of morphology and film thickness effects would be an important avenue for future work. Third, we noted that the two strongly reducing gases, NH_3_ and H_2_S, were likely reacting strongly with the edge sites and defect sites of the COFs, according to spectral features we assigned to the reduction of edge- and defect-site carbonyl groups. XPS showed that the reaction of H_2_S with the COFs resulted in the production of oxidized S species (relative to H_2_S). These three key insights generated from our spectroscopic investigations present an understanding of how these MPc units embedded in frameworks interact with their gas-phase environments that is comprehensive across a systematic array of metal identities.

### Pattern Recognition Algorithms for Discriminating the Analytes using the Response of the COF Array Consisting M-COF-DC-8 (M = Fe, Co, Ni, and Cu)

#### Principal Component Analysis (PCA)

We applied principal component analysis (PCA)^110–113^ to visualize the response of the COF array to 5, 10, 20, 40, and 80 ppm of NO, CO, H_2_S, and NH_3_ in dry N_2_. As a baseline, we also conducted two “null” exposure experiments where the COF array was exposed to pure N_2_ (no analyte, Ø). Our aim was to qualitatively evaluate the ability to discriminate between the carrier gas, NO, CO, H_2_S, and NH_3_ analytes, and ppm-level concentrations thereof, based on the response of the COF array. As an unsupervised dimensionality reduction method, PCA is commonly used to visualize and discover patterns in the high-dimensional responses of gas sensor arrays.^114^ More detailed discussion can be found in Section XIV of the Supporting Information.

To prepare for PCA, we represented the response pattern of the COF array under each gas exposure with a 12-dimensional vector by concatenating three (signed) features of the response curve (i.e., −Δ*G*/*G*_0_ vs time) of each of the four sensors: (1) the initial slope, (2) the extremum, and (3) the area under the curve (see Figure S49). We then transformed each response feature to resemble a Gaussian-like distribution with mean zero and unit variance. Figure 5a visualizes the transformed response vectors of the COF array to each gas exposure.

**Figure 5:**
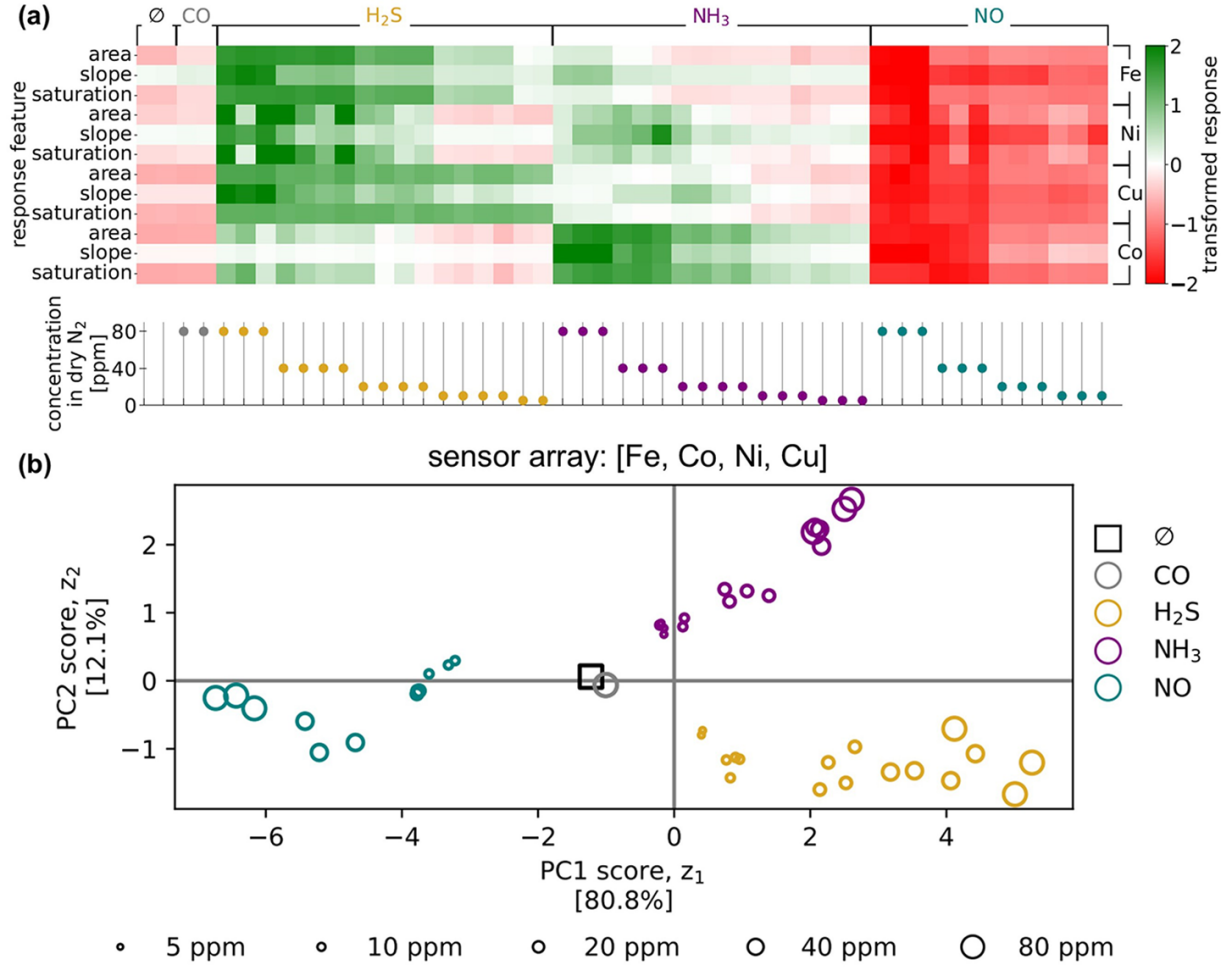
PCA to evaluate the ability to discriminate between the carrier gas, NO, CO, H_2_S, and NH_3_ analytes and different concentrations of them based on the response of the COF array consisting of M-COF-DC-8 (M = Fe, Co, Ni, and Cu). (a) A heatmap (top) visualizing the data matrix, whose columns contain the transformed response vectors of the COF array to different gas compositions (all dry N_2_ carrier) indicated on the bottom. (b) The two-dimensional encodings of the transformed response vectors of the COF array, obtained via PCA of the data matrix.

Next, we employed PCA to compress each transformed COF sensor array response vector into a two-dimensional encoding/latent vector for the purpose of visualization. With an effective array, we expect the response vectors to the same/similar (different/dissimilar) analytes to congregate (separate) in the response space. More, we expect the clusters of responses, grouped by analyte, to be organized according to concentration. Figure 5b shows the PCA encodings of the response vectors. First, the percentage of the variance in the response vectors in the 12-dimensional space explained by each principal component is indicated in the corresponding axis label; the first two principal components together explain approximately 93% of the variance in the response vectors. Thus, the scatter of the encodings in 2D is a rather faithful visual representation of the response vectors in the 12-dimensional space. Second, we observe that the responses are well-clustered according to the analyte. With only the first PC, we can discriminate NO and CO from H_2_S and NH_3_; two PCs are needed to distinguish between all four analytes. Third, the response vectors to each NO, H_2_S, and NH_3_ are roughly arranged, via distance from the origin, according to concentration. These observations are compelling evidence that the response of the COF array contains sufficient information to discriminate NO, CO, H_2_S, and NH_3_ analytes, and different concentrations thereof, in dry N_2_.

The two baseline response vectors, also displayed in Figure 5, lie near the CO response vectors. This reflects the lack of a distinguishable response of the Co-, Ni-, Cu-COFs to CO. Even for the Fe-based sensor, which does respond to CO, its response to CO is small in magnitude compared to its responses to the NO, H_2_S, and NH_3_ analytes, which dominate the spread in the PCA plot. Consequently, we propose a two-stage approach to distinguish between NO, CO, H_2_S, NH_3_, and null. First, use the entire COF array response vector to discriminate between NO, H_2_S, NH_3_, and (CO or null). Then, in the case of a CO or null result, use only the response of the Fe-COF to decide if CO is present or if the gas is pure N_2_. See Figures S50 and S51 for a visualization of the decision tree and a PCA plot showing the Fe-COF distinguishing CO from null exposures.

#### Nearest Neighbors Classification

Next, we turned to supervised machine learning to further assess the ability to discriminate NO, CO, H_2_S, NH_3_, and Ø using the COF array. Like in our PCA, we represented each response of the COF array to a gas exposure as a 12-dimensional vector containing features of each sensor’s response. Unlike PCA, each response vector was then labeled with the identity of the analyte (NO, CO, H_2_S, NH_3_, Ø) that caused the response. First, we partitioned the labeled examples into a train set and test set. We split the responses to NO, H_2_S, and NH_3_ according to concentration (train: 5, 10, 40 ppm; test: 20, 80 ppm). For CO, we allocated one of the two 80 ppm responses to the train set and the other to the test set. For Ø, we put one response in the train set, the other in the test set (see Figure S52). Second, we transformed the response vectors in the train set, then applied the same transformation to those in the test set. Next, we trained a nearest neighbor classifier on the train partition. The nearest neighbor classifier assigns a label to a new (test) response vector according to the label of the closest response vector in the train partition. We used the classifier to make predictions on the response vectors in the test set and compared the predictions to the true labels. Note, we used the two-stage classification shown in Figure S50. The confusion matrix is shown in Figures S53 and S54. Perhaps unsurprisingly given the separation according to analyte that we found from PCA in Figure 5b, the classifier achieves perfect predictions on the test set. Figure S55 gives a high-level overview of our machine learning workflow.

### Challenging the COF Array with Potential Interferants

Since many sensors must function in air and humid environments,^115,116^ the COF array was challenged using different carrier gases instead of dry N_2_, which was the carrier in all previously mentioned sensing experiments. To challenge the COF array’s ability to differentiate the four analytes under more realistic application conditions, sensing exposure to analytes was performed in dry air, humid N_2_ (18% relative humidity (RH)), and humid air (18% RH). All devices were exposed to 80 ppm analyte, which was the highest concentration used in dry N_2_ experiments (see Figure 3), to attribute any difficulty to solely the sensing environment and not low concentration of analytes. The description of humidity sensing set up can be found in Section XII of the Supporting Information. Based on sensing traces of M-COF-DC-8 (M = Co, Ni, and Cu) exposed to 80 ppm analyte in various environments, the sensing performance in terms of direction and order of magnitude of response was generally retained for NO, H_2_S, and NH_3_ but diminished for CO (Figures S19–S22). The maximum response during 30 min of analyte exposure and the initial RoR extracted from the sensing traces (Figure S25) shows the effect of different carrier gases on the ability of different COFs to sense 80 ppm of NO, CO, H_2_S, and NH_3_. This general high-fidelity performance demonstrates the selective ability of the COF devices to retain response to key analytes in the presence of potential interferants in humid air environments.

However, in some cases, there were notable deviations in sensor performance as a result of the carrier gas environment. For instance, the initial RoR of Co-COF-DC-8 to NH_3_ exhibited different initial directionality of response depending on the carrier gas identity, and Ni-COF-DC-8 exhibited a 2-fold increase in response to NO in the presence of humid N_2_ when compared to other carrier gases. A more detailed discussion of these anomalies is found in Section XII of the Supporting Information. This deviation in both maximum response and initial RoR does not necessarily limit the COF array’s utility but instead suggests that these specific analyte-COF device pairings require calibration for specific environmental conditions.

#### Robustness of Gas Discrimination in the Presence of Humidity

To evaluate the ability of the COF array to discriminate between the analytes under humid conditions, we conduct PCA on the response vectors on the *N* = 3 COF array consisting of M-COF-DC-8 (M = Co, Ni, and Cu), constructed from features of each sensor’s response (as in the PCA above). First, a PCA model was trained on the responses under dry N_2_ and dry air. Then, the response vectors to NO, CO, H_2_S, and NH_3_ at 80 ppm with humidity present were projected onto the learned PCs. The data in Figure 6 shows that the responses to all four analytes in the presence of humidity fell into the same analyte-based clusters as those under dry conditions. So, remarkably, the 3-COF array retained its ability to discriminate NO, H_2_S, and NH_3_ despite the presence of water vapor, which notoriously tends to interfere with the response of chemiresistive sensors and confound the predicted analyte composition.^115^ Due to the diminished sensing performance of the COFs for CO in humid environments, the COF array fails to meaningfully discriminate CO from the carrier gases. The general robustness to humidity we observe demonstrates the utility of conductive MPc-based COFs for detecting and discriminating these analytes in realistic environments without the need for engineering complicated strategies to cope with or prevent humidity from interfering with the response.

**Figure 6:**
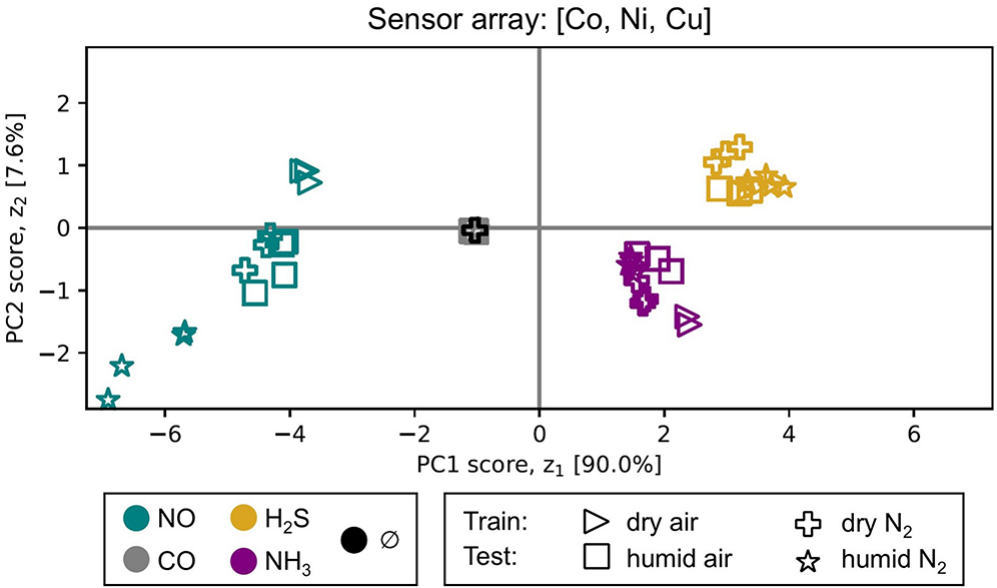
Evaluating the ability to discriminate between NO, CO, H_2_S, and NH_3_ analytes at 80 ppm from the response of the COF array consisting of M-COF-DC-8 (M = Co, Ni, and Cu), in the presence of humidity. PCA plot showing the compressed response vectors under (i) dry conditions, obtained from training, and (ii) humid conditions, obtained by projecting onto the PCs learned from dry conditions.

Note, Fe-COF-DC-8 was left out of the COF array challenged with different carrier gas environments despite the material’s optimal performance for sensing CO in dry N_2_ environments. Due to batch-to-batch material effects, some Fe-COF-DC-8 devices exhibited diminished CO sensing in dry N_2_ environments depending on the specific COF batch (Figure S23) and device fabrication method (Figure S24), which limited its use in other experiments with different carrier gas environments. More detailed discussion can be found in Section XII of the Supporting Information prior to Figure S23.

### Sensor Importance Study

We next investigated if all four COF sensors are required for the discrimination of the NO, CO, H_2_S, and NH_3_ analytes in dry N_2_. We did so by (1) virtually eliminating sensor(s), via removing the response features from the data matrix in Figure 5a that are associated with the eliminated sensor(s), then (2) retraining a PCA model and visually inspecting the quality of the clustering of the responses according to the analyte. The PCA plots for each combination of sensors are displayed in Figures S56–S58. With *N* = 3 sensors, the capability to robustly discriminate NO, CO, H_2_S, and NH_3_ was disturbed the most when the Co- or Cu-COF was removed. With *N* = 2 sensors, the COF arrays consisting of (1) M-COF-DC-8 (M = Cu, and Co) and (2) M-COF-DC-8 (M = Fe, and Co) best retained the ability to discriminate the NO, H_2_S, and NH_3_ analytes and different concentrations of them. With just *N* = 1 sensor, only the Cu-COF sensor could discriminate the analytes. Thus, the choose-one sensor analysis suggests the Cu-COF is most important, to differentiate NO, CO, H_2_S, and NH_3_. Finally, the sensor removal analysis suggests the Co- and Cu-COFs are the most important, which is consistent with the choose-two sensors analysis. Of course, for detecting CO (i.e., distinguishing it from the carrier gas), the Fe-COF is most important since it is the only COF that shows a response to CO.

### Limitations

Despite these advantages, the proposed COFs possess limitations, such as lower crystallinity and conductivity compared to MOFs due to less reversible linkage formation and, ergo, limited kinetic control over crystal growth. Additionally, the synthetic challenges in accessing reliably crystalline M-COF-DC-8 materials hindered the inclusion of all 4 materials in the COF array subjected to humidity. However, these limitations may be surmounted by continued study of COF crystal growth, and strategic development of synthetic methods for this class of materials and precursors.

## Conclusion

The current contribution is the first report of a chemiresistive COF array for the gas-phase detection and differentiation of single analytes exposure (NO, CO, H_2_S, and NH_3_) with low, distinguishable LODs. Our strategy employed four derivatives of M-COF-DC-8 (M = Fe, Co, Ni, and Cu), which provided two principal advantages. First, the array of distinct, but structurally analogous M-COF-DC-8 materials provided the ability to detect and differentiate each of the analytes by varying the surface chemistry of each COF and thus providing opportunity for nuanced chemiresistive responses due to specific analytes. Second, the sliding scale of transition metals we accessed allowed us to investigate how MPc structural motifs can be used to control the host–guest chemistry within conductive frameworks and how incorporating MPc units into a stacked 2D material impacts the properties of each metal center.

The success of our approach can be seen in the sensing properties of our chemiresistive array. First, the 4 COF array in dry N_2_ conditions showed ppb-level theoretical LODs toward NO: 0.4 ppb, CO: 860 ppb, H_2_S: 28 ppb, and NH_3_: 75 ppb using Cu-COF-DC-8, Fe-COF-DC-8, Cu-COF-DC-8, and Ni-COF-DC-8, respectively. Second, the response of the COF array enabled discrimination between the NO, CO, H_2_S, and NH_3_ analytes in dry N_2_ conditions using both PCA (unsupervised machine learning) and the nearest neighbor classifier (supervised). That is, the contributions of each of the COFs provided sufficient information to allow differentiation of the gases using the overall response pattern of the COF array. Even in the presence of potential interferants (e.g., O_2_, H_2_O, and CO_2_), the 3 COF array consisting of M-COF-DC-8 (M = Co, Ni, and Cu) retained its chemiresistive response to gasotransmitters and NH_3_ for detection and differentiation. The targeted host–guest interactions between analytes of interest and COF dominate to enable differentiation despite interferents present.

The spectroscopic study we performed using DRIFTS, XPS, and EPR with probe gases revealed three primary chemical features of the frameworks relevant to chemical sensing. First, the Lewis acidity of the COFs was controlled by the metal center. Fe-COF-DC-8 exhibited the strongest Lewis acid sites, followed by Co-COF-DC-8, Cu-COF-DC-8, and Ni-COF-DC-8, which follows a previously reported adsorption strength of MPc molecules toward NH_3_.^109^ Second, the identity of the metal center in the MPc units controlled the ability of the framework to adsorb guest molecules that undergo σ-donation and π-back bonding. Only Co-COF-DC-8 and Fe-COF-DC-8 hosted strong adsorption sites for NO, while we observed that with material of moderate crystallinity only Fe-COF-DC-8 was able to appreciably bind CO at the phthalocyanine metal center. This result is direct evidence that incorporating MPc molecules into framework materials allows for direct control over surface reactivity. Third, we observed that the reducing gases H_2_S and NH_3_ reacted strongly with defect sites on the COFs leading to their reduction. These spectroscopic trends correlated well with sensing results for these reducing gases.

This work advances the understanding of host–guest interactions within MPc-embedded frameworks. It demonstrates that the capabilities of a sensor array for detection and differentiation can be controlled through bottom-up molecular design and that single atom substitution to the unit cell of a framework can greatly impact the sensing properties of the frameworks in a predictable manner. As the use of MPc-embedded materials continues to become more prominent and continues to expand into more divergent electronically transduced applications, understanding how structural properties and atomic identities of the frameworks impact chemical properties will become an increasingly important factor to understand and control. This fundamental work paves the way for future studies of MPc-based framework materials to serve as chemiresistors in practical applications where mixture deconvolution and resistance to humidity and degradation is imperative.^117–120^ Future studies should target optimal material configuration, device form factor, and machine learning techniques to function in realistic conditions. The use of anti-humidity strategies (hydrophobic membranes, and physical isolation, etc.) during device fabrication can be an effective strategy to enhance analyte detection and discrimination in complex environments.^115^ Additionally, future investigations should include extended adsorption–desorption cycles to fully assess device lifespan and stability in the presence of these highly reactive gases. This work uncovers important fundamental properties of transition metal phthalocyanines at the interface of framework materials that we anticipate will have a great impact on the development of future systems for electronically transduced applications such as chemical detection.

## Supplementary Material



## Notes

Additional experimental details, synthetic optimizations, material characterization, chemiresistive sensing traces, machine learning, spectroscopic analyses of material–analyte interactions, and computational models (PDF)
