# Supplementary material for: Conductive Covalent Organic Frameworks as Chemiresistive Sensor Arrays for the Detection and Differentiation of Gasotransmitters
Source: J Am Chem Soc. 2025 Nov 13;147(47):43438–52. doi: 10.1021/jacs.5c11454 (PMC12673599; doi:10.1021/jacs.5c11454)
Supplement: Supplementary file 1 [file ja5c11454_si_001.pdf]

Conductive Covalent Organic Frameworks as Chemiresistive Sensor Arrays for the Detection and Differentiation of Gasotransmitters

Georganna Benedetto[1#], Robert M. Stolz[1#], Zheng Meng[1#], Joseph Y. M. Chan[1], Elissa O. Shehayeb[1], Colin T. Morrell[1], Gbenga Fabusola[2], Nikolaus Elsaesser[3], Cory M. Simon[2,*], and Katherine A. Mirica[1,*]

[1]Dartmouth College, Department of Chemistry, Hanover, NH 03755, USA
[2]Oregon State University, School of Chemical, Biological, and Environmental Engineering, Corvallis, OR 97331, USA
[3]Oregon State University, Department of Mathematics, Corvallis, OR 97331, USA
*email: cory.simon@oregonstate.edu, Katherine.a.mirica@dartmouth.edu
# contributed equally

SUPPORTING INFORMATION

**I.**	**Methods**	**S2**
**II.**	**Synthesis of Phthalocyanine COF Precursors**	**S4**
**III.**	**Synthesis of M-COF-DC-8 (M=Fe, Co, Ni, Cu)**	**S12**
**IV.**	**Elemental Analysis of M-COF-DC-8 materials**	**S25**
**V.**	**X-Ray Photoelectron Spectroscopy (XPS) of Pristine M-COF-DC-8 Materials**	**S26**
**VI.**	**Diffuse Resonance Infrared Fourier Transform Spectroscopy (DRIFTS) of Pristine M-COF-DC-8 Materials**	**S31**
**VII.**	**Electron Microscopy of M-COF-DC-8 Materials**	**S32**
**VIII.**	**Thermal Gravimetric Analysis of Pristine M-COF-DC-8 Materials**	**S35**
**IX.**	**Electron Paramagnetic Resonance (EPR) Spectroscopy of Pristine M-COF-DC-8 Materials**	**S36**
**X.**	**Specific Surface Area of M-COF-DC-8 Materials**	**S37**
**XI.**	**Conductivity of M-COF-DC-8 Materials**	**S39**
**XII.**	**Chemiresistive Gas Sensing**	**S40**
**XIII.**	**Spectroscopic Characterization of M-COF-DC-8 materials with Probe Gases by DRIFTS, XPS, and EPR**	**S52**
**XIV.**	**Methodology for PCA of the Sensor Array Response Data**	**S79**
**XV.**	**Computational Modeling of Host–Guest Interactions and Properties**	**S88**
**XVI.**	**References**	**S94**

I. Methods

Ethylenediaminetetraacetic acid, tetrasodium salt dihydrate (EDTA•4Na, CAS N.O.: 10378-23-1) was purchased from Acros Organics, Thermo Fisher Scientific Inc. Acetone (CAS N.O.: 67-64-1) was purchased from BDH Chemicals. Mesitylene (CAS N.O.: 108-67-8), and *p*-toluene-sulfonyl chloride (CAS N.O.: 98-59-9) were purchased from BeanTown Chemical. 200 proof ethanol (CAS N.O.: 64-17-5) was purchased from Decon Labs. Dichloromethane (CAS N.O.: 75-09-2) was purchased from Macron Chemicals. 36.5-38 % hydrochloric acid (CAS N.O.: 7647-01-0), *N,N*-Dimethylformamide (DMF, CAS N.O.: 68-12-2), *N*-hexanol (CAS N.O.: 111-27-3), and *p*-toluenesulfonic acid, monohydrate (CAS N.O.: 6192-52-5) were purchased from MilliporeSigma. 25%-28% ammonium hydroxide (CAS N.O.: 1336-21-6), bromine (CAS N.O.: 7726-95-6), 1,8-diazabicyclo[5.4.0]undec-7-ene (CAS N.O.: 67-56-1), nickel(II) chloride (CAS N.O.: 7718-54-9), copper(II) acetate (CAS N.O.: 142-71-2), iron(II) chloride (CAS N.O.: 7705-08-0), cobalt(II) chloride(CAS N.O.: 7646-79-9), pyridine (CAS N.O: 110-86-1), and 98% sulfuric acid (CAS N.O.: 7664-93-9) were purchased from Sigma-Aldrich Corporation. Dimethylacetamide (DMAC, CAS N.O.: 127-19-5), 1,2-dichlorobenzene (DCB, CAS N.O.: 95-50-1), *N*-methyl-2-pyrrolidone (CAS N.O.: 872-50-4), and *o*-phenylenediamine (CAS N.O.: 95-54-5) were purchased from TCI America. Ethyl acetate (CAS N.O.: 141-78-6), glacial acetic acid (CAS N.O.: 64-19-7), methanol (CAS N.O.: 67-56-1), and petroleum ether (CAS N.O.: 64742-49-0) were purchased from VWR Life International. Anhydrous sodium acetate (CAS N.O.: 127-09-3), and sodium hydroxide (CAS N.O.: 1310-73-2) were purchased from VWR Life Science.

Pyrene-4,5,9,10-tetraone (TOPyr CAS N.O.: 14727-71-0) was synthesized by following the literature procedure.[1] The yields were given as isolated yields.

NMR spectra were recorded on a Bruker™ 600 MHz spectrometer. The chemical shifts (δ) were expressed in ppm with internal standard tetramethylsilane (TMS), solvent signals as internal references, and *J* values were expressed in Hz. Standard abbreviations indicating multiplicity were as follows: ss (singlet), br (broad), d (doublet), t (triplet), q (quartet), and m (multiplet). Infrared spectra were collected using a JASCO™ model FT IR-6100 Fourier transform infrared spectrophotometer. X-ray photoelectron spectroscopy (XPS) experiments were conducted on a Kratos Analyitcal™ AXIS Supra X-ray Photoelectron Spectrometer equipped with a monochromatic Al (Kα) X-ray source under ultrahigh vacuum (base pressure 10^{-9} Torr, tube current: 10 mA, voltage: 15 kV). Both survey and high-resolution spectra were obtained using a beam diameter of 200 μm. The pass energy for the low resolution survey san was 0−1200 eV. The pass energies for the high resolutions scans were as follows: C (275−303 eV), N (390−413 eV), O (525−546 eV), S (160−175 eV), Fe (700−743 eV), Co (770−813 eV), Ni (843−888 eV), Cu

(920−970 eV). XPS samples dosed with analytes were exposed to 1% analyte (balance N_2), capped, and then shipped to be analyzed. The Shirley background and Gauss Lorentz fitting were used to fit the XPS spectra and all scans were referenced to adventitious carbon. S 2p regions were analyzed considering the spin-orbital components of a 1.16 eV offset and a 0.5:1 ratio of 2p 1/2:3/2. Elemental analyses (including the elements C, H, N) were performed by Atlantic Microlab Inc. using a combustion method by automatic analyzers. Metal content analyses were performed by the Dartmouth Trace Element Analysis Core using an Agilent™7900 inductively coupled plasma mass spectrometer (ICP-MS) on samples digested with nitric acid. Powder X-ray diffraction (PXRD) measurements were performed with a Rigaku sixth generation MiniFlex X-ray diffractometer with a 600 W (40 kV, 15 mA) CuKα (α = 1.54 Å) radiation source. Scanning electron microscopy (SEM) was performed on a Thermo ScientificTM HeliosTM 5 CX DualBeam (Waltham, MA) instrument equipped for X-ray analysis with Oxford Instruments UltimMax 100 and Oxford Instruments Ultim Extreme 100 detectors. (Abingdon, United Kingdom).

Imaging was performed using a 15 kV beamline, with a 10 pA beam current at a working distance of 7 mm in a 10-6 torr vacuum chamber. Transmission electron microscopy was carried out on a FEI™Tecnai F20ST FEG TEM instrument. An operating voltage of 120 kV was used for imaging.

Gas sensing experiments were conducted using two Sierra Micro-Trak and a Smart-Trak mass flow controllers in combination to deliver concentrations of gases from custom-ordered pre-mixed tanks (10,000 ppm of NH_3, H_2S, and NO in N_2) supplied by Air Gas, and equipped with two-stage stainless steel regulators. Gas streams from the tanks were diluted with dry N_2. Target gas concentrations were adjusted by controlling the analyte gas and balance gas flow rates with two mass-flow controllers. The flow of balance/purging gas was controlled at 0.5 or 1.0 L/min and the flow of the analyte was controlled between 0.25 to 4.0 mL/min. Fabricated sensors built on 5 μm interdigitated gold electrodes (part N.O.: G-IDEAU5, purchased from Metrohm) were sealed in a custom Teflon chamber and connected to a potentiostat and the current of these devices was measured using the chronoamperometry analytical method. The applied voltage was set at 1.0 V and the time interval for recording current was set at 0.5 s.

Electron paramagnetic resonance (EPR) spectra were collected on a Bruker BioSpin Gmbh spectrometer equipped with a standard mode cavity. High-resolution mass spectroscopy was performed at the University of Illinois Urbana-Champaign.

II. General Synthesis of Phthalocyanine COF Precursors

Tosylation of *o*-phenylenediamine

Into a 2 L RBF equipped with a stir bar was added tosyl chloride (420 g, 2.2 mol, 2.04 eq.) and pyridine (1 L). The reaction was cooled to a temperature between -10 – -20°C using a salt-ice water bath. Once cooled, *o*-phenylenediamine (*o*-PD, 117 g, 1.08 mol, 1 eq.) was added in 5 g portions to prevent overheating. Once the addition was complete, the reaction was allowed to return to room temperature and stirred overnight while covered. The reaction was poured into a 4 L Erlenmeyer flask. The reaction was very slowly diluted with 500 mL of DI water and quenched slowly with HCl (38 %) until no pyridine smell was detected. The reaction was then vacuum filtered through a 600 mL fritted filter and washed with EtOH (200 proof) and DI water until the filtrate ran clear. The solid product was put in a 1 L beaker with EtOH and boiled for ~20 minutes. The solid did not fully dissolve in EtOH. The reaction was cooled, covered, and allowed to recrystallize in a refrigerator (7°C) overnight. The product was then isolated by vacuum filtration and the solid was tried overnight under reduced pressure to yield off-white crystals. (> 400 g, >95%). Characterization matched previous reports.[2]

Bromination of N,N'-(1,2-Phenylene)bis(4-methylbenzenesulfonamide).

To a 1 L RBF equipped with a large stir bar was added tosylated diamine (100 g, 0.24 mol, 1 eq.), anhydrous NaOAc (41.3 g, 0.50 mol, 2.1 eq.), and 300 mL of glacial AcOH. The solids and AcOH were stirred into suspension. The reaction was stirred vigorously at room temperature while Br_2 (24.6 mL, 2 eq.) was added dropwise using an addition funnel that was lightly capped to prevent pressure build-up but prevent ingress of dust and excess air exchange. Addition rate of Br_2 can be as fast as 1-2 mL/10 min, or as fast as red color dissipates and returns to a cream or off-white color after addition of Br_2. The rate of addition of Br_2 should become progressively slower as the reaction progresses. Glacial AcOH was added to the RBF as needed to keep a consistent viscosity of the suspension (Br_2 addition increases viscosity). This addition was accomplished by lifting the addition funnel and pouring in additional AcOH, Typically, a reaction using 100 g of tosylated diamine required the addition of 100 mL of additional AcOH. After the addition of Br_2 was completed, the reaction was allowed to stir for an additional 90 min at RT. The color of the reaction should be a light cream color. The reaction vessel was fixed with a water jacket condenser and was then heated to reflux (hot plate set to 120°C, RBF wrapped with Al-foil) overnight. The next morning, the reaction was filtered through a fine fritted filter. The solids were washed with AcOH (50 mL x 2) and recrystallized from boiling EtOH (filter from cold EtOH). The solid was

dried overnight in a vacuum oven to provide 110 g of the dibrominated product as an off-white powder. The product was stored at RT under air. Characterization matched previous reports.[2]

Cyanization of N,N'-(4,5-dibromo-1,2-phenylene)bis(4-methylbenzenesulfonamide) .

A 50 mL 3-neck round bottom flask was charged with 2.00 g of A (1.0 eq., 3.48 mmol) and 1.25 g CuCN (4.0 eq., 13.96 mmol). The contents were degassed by three cycles of vacuum purging for 2 minutes followed by dosing with Ar. With Ar blowing through the reaction vessel, 10 mL of DMAC was added with a glass syringe and the reaction vessel was capped. The reaction vessel was heated from RT to 120°C. The reaction vessel was stirred and heated at 49°C with Ar bubbling slowly (~1 bubble/sec) for 48 hrs. Upon cooling to room temperature, the reaction mixture was diluted with 150 mL of 14.8 M NH_4OH. The mixture was vigorously stirred with air bubbling into it for ~4 hrs. The brown solid product was then isolated using vacuum filtration and washed with copious water until the filtrate ran clear and yielded a pH of ~7. The solid was then dried on the vacuum line for 20 mins. At the end of drying the color was a taupe/brown. The solid was collected and diluted with 25 mL acetone and 3 mL AcOH. The mixture was sonicated for 2 minutes to ensure the product dissolved. (Note: some brown solid did not dissolve but this is not product). The mixture was vacuum filtered to isolate the purple filtrate from the minimal brown solid. The filtrate was poured into 200 mL of 90 M Ethylenediaminetetraacetic acid tetrasodium salt, dihydrate solution. The mixture color changed to dull blue then dull green. 200 mL DI H_2O was subsequently added to the mixture, followed by 2 mL aliquots of AcOH over the course of 1 hour (12 mL total AcOH) The beaker was left for 20 minutes to let product precipitate out of solution. The light tan/yellow solid product was isolated via vacuum filtration from the bright blue filtrate. The solid was dried in a 49°C vacuum oven overnight. (0.75 g, 46%). ^{1}H NMR (500 MHz, 298K, DMSO- d_6) δ = 7.66 (*d, J*=10 Hz, 4H), 7.59 (s, 2H), 7.36 (*d, J*=10 Hz, 4H), 2.37 (s, 6H) ppm. ^{13}C NMR (600 MHz, DMSO- d_6): δ= 144.12, 136.02, 133.90, 129.97, 126.85, 123.92, 115.59, 109.40, 21.02 ppm.

Synthesis of 2,3,9,10,16,17,23,24-octaamino-phthalocyanine iron (II) **FePc(NH2)8**

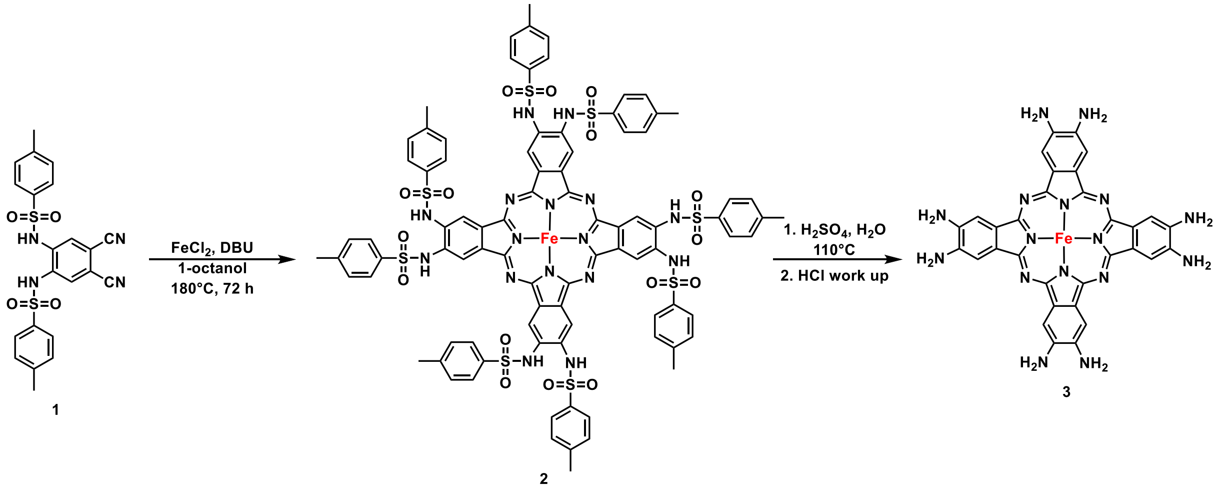

Scheme S1. The synthetic scheme towards **FePc(NH2)8** precursor.

Synthesis of 2,3,9,10,16,17,23,24-octatosylamino-phthalocyanine iron(II) (2). The synthesis of the 2,3,9,10,16,17,23,24-octatosylamino-phthalocyanine Fe(II) was adapted from the literature.[S3] To a suspension of 1.00 g of (2 mmol) **1** in n-octanol (3 mL) in a glass pressure vessel, 0.324 g of $FeCl_2$ (2 mmol, 1.0 eq) and 2 mL of DBU (1,8-diazabicyclo[5.4.0]undec-7-ene) were added. The system was purged with Argon (Ar) for 10 minutes and then sealed. The reaction vessel was heated at 180°C and stirred for 72 hours under Ar. Upon cooling to room temperature, the reaction mixture was diluted with a 100 mL solution of Dichloromethane (DCM): Acetic acid (AcOH) (5:1). The organic extract was washed with deionized water (300 mL × 3) and evaporated to dryness to afford a blue oil. To remove the residual high boiling point hexanol remaining, the product was fully dissolved in DCM (4 mL). 100 mL of petroleum ether was added to the solution to afford a precipitate, which was isolated via filtration and washing in 200 additionally mLs of petroleum ether. The blue crude product was them washed with AcOH until the filtrate ran less dark. Into a clean Erlenmeyer flask, the crude solid product was dissolved in 60 mL of DCM: MeOH (10:1). 150 mL of AcOH was added to the filtrate and placed in a fridge for 2 days to recrystallize to produce dark blue crystals (0.049 g, 5%). ^{1}H NMR (600 MHz, 298K, Acetone- d_6) δ = 9.61 (b, 8H), 8.98 (b, 5H), 7.90 (s, 16H), 7.45 (s, 16H), 2.40 (s, 24H) ppm. HRMS (EI+): m/z calculated for $C_{88}H_{73}FeN_{16}O_{16}S_8$: 1920.24; found: 1920.24, 1921.24, 1922.25.

Synthesis of 2,3,9,10,16,17,23,24-octaamino-phthalocyanine iron (II) (FePc(NH$_2$)$_8$) (3). To a 50 mL round bottom flask charged with 1.54 g of **2**, a mixture of 9.25 mL deionized water and 92.46 mL sulfuric acid (H_2SO_4) was added. The mixture was heated for 50 mins. The reaction was then cooled to room temperature, and the reaction mixture was poured into ice-water (100 mL). The dark blue precipitate was divided into four falcon tubes collected by centrifugation. Each sample of precipitate was washed thoroughly with hydrochloric acid (1M) (25 mL × 2), and tetrahydrofuran (20 mL × 3) sequentially with the help of a vortex. Product **3** was obtained as a purple to black powder (0.374 g, 48%). HRMS (EI+): m/z calculated for $C_{32}H_{25}FeN_{16}$: 688.1719; found: 960.5.

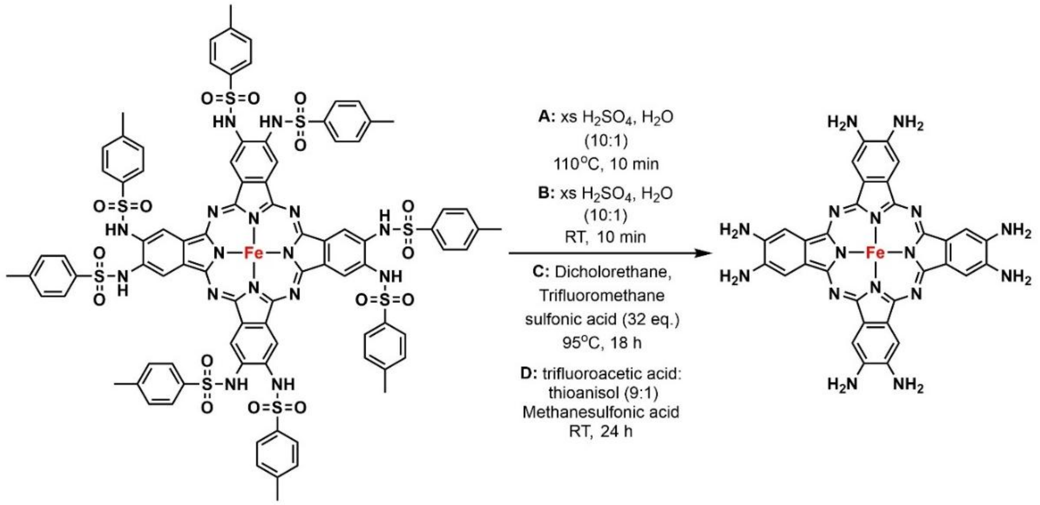

Scheme S2. Various synthetic trials (A−D) used for deprotecting FePc(NH$_2$)$_8$.

Various attempts were made to deprotect tosyl-protected FePc(NH$_2$)$_8$ to yield a precursor with the correct mass spectrum, but all attempts did not yield characterizable precursor. The material could not be identified via NMR or HRMS. However, material using synthetic trial A was used to successfully synthesize Fe-COF-DC-8 as seen in **SI Section III**. In leu of characterization, the presence of crystalline COF ultimately verifies the presence of the FePc(NH$_2$)$_8$ precursor.

Synthesis of 2,3,9,10,16,17,23,24-octaamino-phthalocyanine cobalt (II) **CoPc(NH$_2$)$_8$**

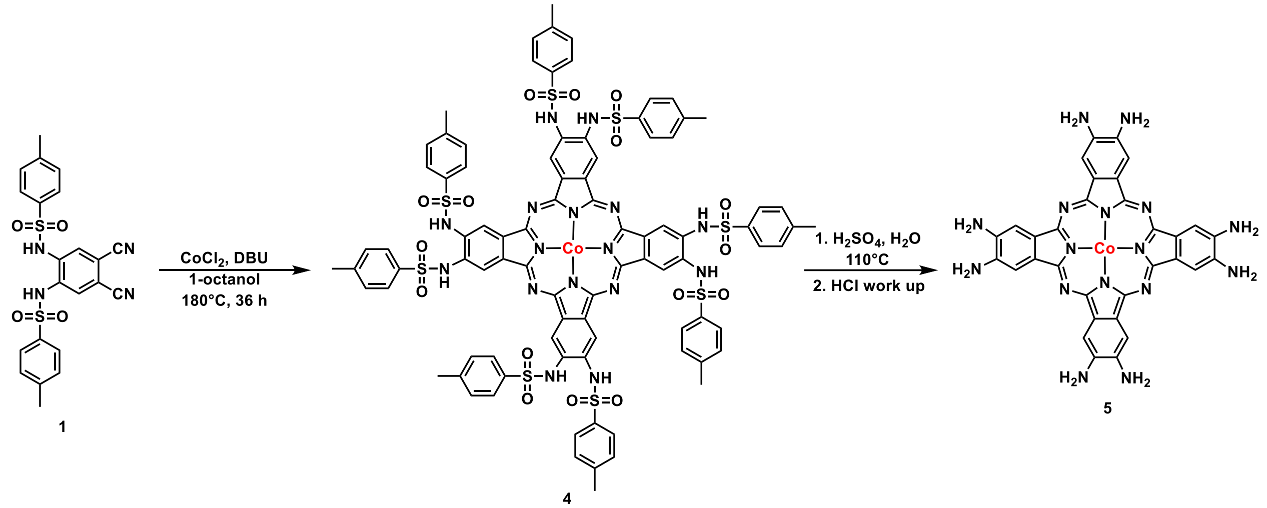

Scheme S3. The synthetic scheme towards **CoPc(NH$_2$)$_8$** precursor.

Synthesis of 2,3,9,10,16,17,23,24-octatosylamino-phthalocyanine cobalt (II) 4. The synthesis of the 2,3,9,10,16,17,23,24-octatosylamino-phthalocyanine Co(II) was adapted from the literature.[S3] To a suspension of 2.00 g of **1** in n-octanol (9.52 mL) in a glass pressure vessel, 0.232 g of CoCl$_2$ (1.0 eq) and 1.12 mL of DBU were added. The system was purged with Ar for 10 minutes and then sealed. The reaction vessel was heated at 180°C and stirred for 36 hours under Ar. Upon cooling to room temperature, the reaction mixture was diluted with a 100 mL solution of DCM:AcOH (5:1). The organic extract was washed with deionized water (300 mL × 3) and evaporated to dryness to afford a blue oil. To remove the residual high boiling point octanol remaining, the product was fully dissolved in DCM (4 mL). 100 mL of petroleum ether was added to the solution to afford a precipitate, which was isolated via filtration and washing in 200 mLs of petroleum ether. The blue crude product was then further purified via silica column (eluent: 2% MeOH in DCM) and recrystallized in 10 mL methanol (MeOH): DCM (3:1) mixture. Product **4** was isolated as dark blue crystals (0.023 g, 1%). ^{1}H NMR (600 MHz, 298K, Acetone-d_6) δ = 10.63 (s, 4H), 9.98 (s, 4H), 9.91 (s, 4H), 9.47 (s, 4H), 9.14 (s, 8H), 8.48 (s, 8H), 7.46 (*d, J*=10 Hz, 8H), 6.66 (*d, J*=10 Hz, 8H), 3.38 (s, 12H), 2.04 (s, 12H). HRMS (EI+): m/z calculated for $C_{88}H_{73}CoN_{16}O_{16}S_8$: 1923.2410; found: 1923.2362, 1924.2435, 1925.2426, 1926.2437.

Synthesis of 2,3,9,10,16,17,23,24-octaamino-phthalocyanine cobalt (II) (CoPc(NH$_2$)$_8$) (5). To a 50 mL round bottom flask charged with 78 mg (0.51 mmol) of **4,** a mixture of 0.245 mL deionized water and 2.45 mL sulfuric acid (H_2SO_4) was added. The mixture was heated for 50 mins. The reaction was then cooled to room temperature, and the reaction mixture was poured into ice-water

(25 mL). The dark blue precipitate was collected by centrifugation and the solid was washed thoroughly with deionized water (30 mL × 3), 10% potassium hydroxide (KOH) (25 mL × 3), deionized water (25 mL x 3), hydrochloric acid (1M) (25 mL × 2), and ethanol (25 mL × 3) sequentially with the help of a vortex. The product **5** was obtained as a purple to black powder (0.142 g, 72%). ^{1}H NMR (600 MHz, DMSO-d_6): δ = 11.61 (b, 8H), 6.05 (s, 16H).

Synthesis of 2,3,9,10,16,17,23,24-octaamino-phthalocyanine nickel (II) **NiPc(NH$_2$)$_8$**

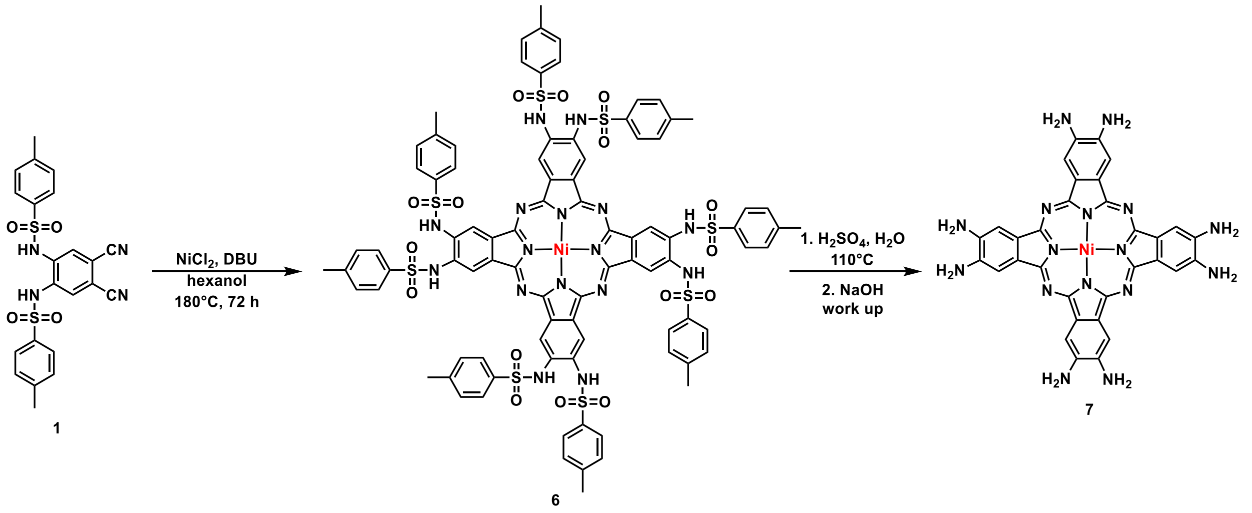

Scheme S4. The synthetic scheme towards **NiPc(NH$_2$)$_8$** precursor.

Synthesis of 2,3,9,10,16,17,23,24-octatosylamino-phthalocyanine nickel (II). The synthesis of the 2,3,9,10,16,17,23,24-octatosylamino-phthalocyanine Ni(II) was performed as previously reported in the literature.[S3] To a suspension of 1.00 g of **1** in hexanol (2.94 mL) in a glass pressure vessel, 0.278 g of $NiCl_2$ (1.0 eq) and 0.588 mL of DBU were added. The system was purged with Ar for 10 minutes and then sealed. The reaction vessel was heated at 180°C and stirred for 36 hours under Ar. Upon cooling to room temperature, the reaction mixture was diluted with a 100 mL solution of DCM:AcOH (5:1). The organic extract was washed with deionized water (300 mL × 3) and evaporated to dryness to afford a blue oil. To remove the residual high boiling point hexanol remaining, the product was fully dissolved in DCM (4 mL). 100 mL of petroleum ether was added to the solution to afford a precipitate, which was isolated via filtration and washing in an additional 200 mLs of petroleum ether. The blue crude product was then further purified via silica column (eluent: 2.5% MeOH in DCM) and recrystallized in 4 mL MeOH: DCM (3:1) mixture. Product **6** was isolated as dark blue crystals (0.455 g, 44%).^{1}H NMR (600 MHz, 298K, Acetone- d_6) δ = 9.14 (b, 6H), 9.01 (s, 8H), 7.92 (*d, J*=6 Hz, 16H), 7.44 (*d, J*=12 Hz, 16H), 2.34

(s, 24H). ^{13}C NMR (600 MHz, Acetone- d_6): δ= 146.43, 145.61, 137.12, 135.14, 133.86, 130.88, 128.55, 118.45, 21.55.

Synthesis of 2,3,9,10,16,17,23,24-octaamino-phthalocyanine nickel (II) (NiPc(NH$_2$)$_8$) (7). To a 50 mL round bottom flask charged with 0.54 g of **6**, a mixture of 1.62 mL deionized water and 16.2 mL H_2SO_4 was added. The mixture was heated for 50 mins. The reaction was then cooled to room temperature, and the reaction mixture was poured into ice-water (40 mL). The dark blue precipitate was collected by centrifugation and the solid was washed thoroughly with acetone (25 mL × 3), 10% sodium hydroxide (NaOH) (25 mL × 3), DI water (25 mL × 3), acetone (25 mL × 4) sequentially with the help of a vortex. The product **7** was obtained as a purple to black powder (0.138 g, 71%). ^1H NMR (600 MHz, DMSO-d_6): δ = 8.34 (s, 8H), 5.65 (s, 16H). ^{13}C NMR (600 MHz, DMSO- d_6): δ= 144.61, 138.70, 128.68, 104.69. HRMS (MALDI): m/z calculated for $C_{32}H_{25}NiN_{16}$: 690.1723; found: 690.1688, 691.1834, 692.1749.

Synthesis of 2,3,9,10,16,17,23,24-octaamino-phthalocyanine copper (II) **CuPc(NH$_2$)$_8$**

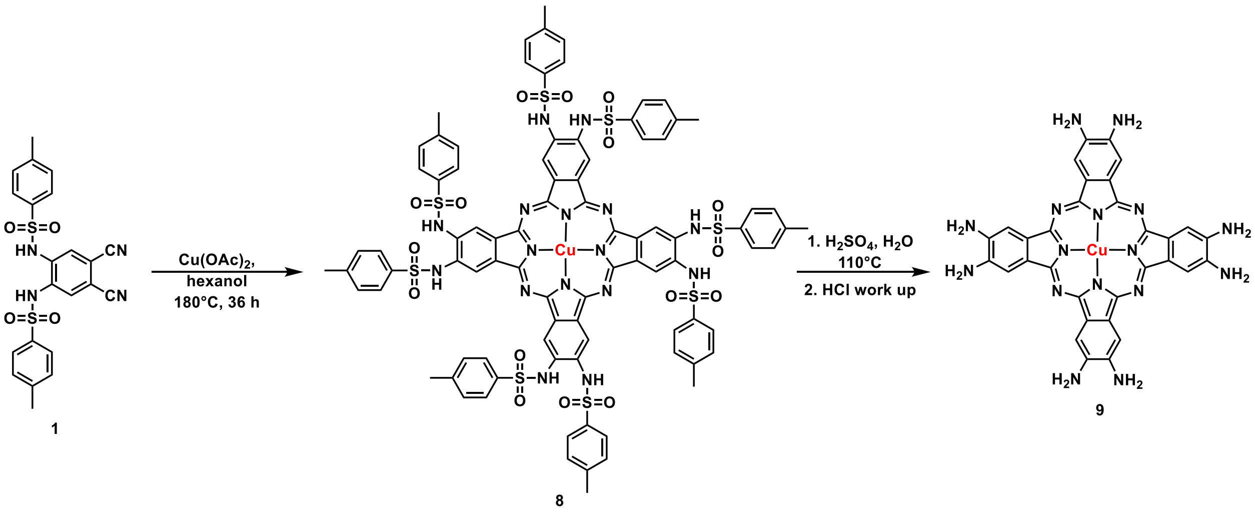

Scheme S5. The synthetic scheme towards **CuPc(NH$_2$)$_8$** precursor.

Synthesis of 2,3,9,10,16,17,23,24-octatosylamino-phthalocyanine copper (II) (8). synthesis of the 2,3,9,10,16,17,23,24-octatosylamino-phthalocyanine Cu(II) was achieved from the tosylated precursor. To a suspension of 1.13 g of **1** in hexanol (5.63 mL) in a glass pressure vessel, 0.131 g of $Cu(OAc)_2$ (1.0 eq) was added. The system was purged with Ar for 10 minutes and then sealed. The reaction vessel was heated at 180°C and stirred for 36 hours under Ar. Upon cooling to room temperature, the reaction mixture was diluted with a 100 mL solution of DCM: AcOH (5:1). The organic extract was washed with deionized water (300 mL × 3) and evaporated to dryness to afford a blue oil. To remove the residual high boiling point hexanol remaining, the product was fully

dissolved in DCM (4 mL). 100 mL of petroleum ether was added to the solution to afford a precipitate, which was isolated via filtration and washing in 200 additionally mLs of petroleum ether. The blue crude product was then further purified via silica column (eluent: 2.5% MeOH in DCM) and recrystallized in 4 mL MeOH: DCM (3:1) mixture. Product **8** was isolated as a bright blue powder. ^{1}H NMR (600 MHz, 298K, Acetone- d_6) δ = 9.21 (b, 5H), 7.82 (s, 16H), 7.41 (s, 16H), 2.34 (s, 24H). HRMS (MALDI): m/z calculated for $C_{88}H_{73}CuN_{16}O_{16}S_8$: 1927.2374; found: 1928.2438, 1929.2543, 1930.2389, 1931.2336.

Synthesis of 2,3,9,10,16,17,23,24-octaamino-phthalocyanine copper (II) (CuPc(NH$_2$)$_8$) (9). To a 50 mL round bottom flask charged with 0.206 g of **8**, a mixture of 0.620 mL deionized water and 6.20 mL H_2SO_4 was added. The mixture was heated for 50 mins. The reaction was then cooled to room temperature, and the reaction mixture was poured into ice-water (25 mL). The dark blue precipitate was collected by centrifugation and the solid was washed thoroughly with deionized water (30 mL × 3), 10% sodium hydroxide (NaOH) (25 mL × 3), deionized water (25 mL × 3), hydrochloric acid (1M) (25 mL × 2), deionized water (25 mL × 3), and acetone (25 mL × 3) sequentially with the help of a vortex. The product **9** was obtained as a purple to black powder ^{1}H NMR (600 MHz, DMSO- d_6): δ = 9.42 (br, 8H), 5.94 (b, 16H). HRMS (ESI): m/z calculated for $C_{32}H_{25}CuN_{16}$: 695.1666; found: 695.1666, 696.1782, 697.1584.

	Solvent	Prec.: M^{n+}	M^{n+}	Additive	Prec.: additive	Results
1	Hexanol	1:1	$CuCl_2$	DBU	1:1.3 wt/wt	Orange-brown solution
2	Ethylene glycol	1:0.25	$CuCl_2$	$(NH_4)_6Mo_7O_{24}$, urea	---	Brown/black precipitate
3	Hexanol	1:3	$CuCl_2$	DBU	1:4	Orange-brown solution
4	Ethylene glycol	1:4	$CuCl_2$	None	---	Brown precipitate
5	Pentanol	1:0.25	CuCl	DBU	1:0.06	N.R.
6	TCB	1:0.25	CuCl	DBU	1:0.75	Orange-brown solution
7	Hexanol	1:0.30	$Cu(OAc)_2$	None (preheat)	---	CuPc(NHTs)$_8$
8	Hexanol	1:1	$Cu(OAc)_2$	None (preheat)	---	Brown precipitate
9	Hexanol	1:0.50	$Cu(OAc)_2$	None (preheat)	---	Brown precipitate
10	Hexanol	1:0.30	$Cu(OAc)_2$	AcOH	10 μM	Orange-brown solution

Table S1. Typical reaction conditions used to synthesize other metal variants of MPc(NHTs)$_8$ did not work for the Cu-derivative. Below are reaction conditions and results of attempts to synthesize CuPc(NHTs)$_8$. Entry 7 provided a successful synthesis of the desired Cu-derivative. Abbreviations: Prec.: precursor (phthalonitrile), TCB: trichlorobenzene, DBU: 1,8-

Diazabicyclo[5.4.0]undec-7-ene, N.R: no reaction. Preheat refers to preheating the oil bath so the reaction is rapidly brought to temperature.

III. Synthesis of M-COF-DC-8 (M=Fe, Co, Ni, Cu)

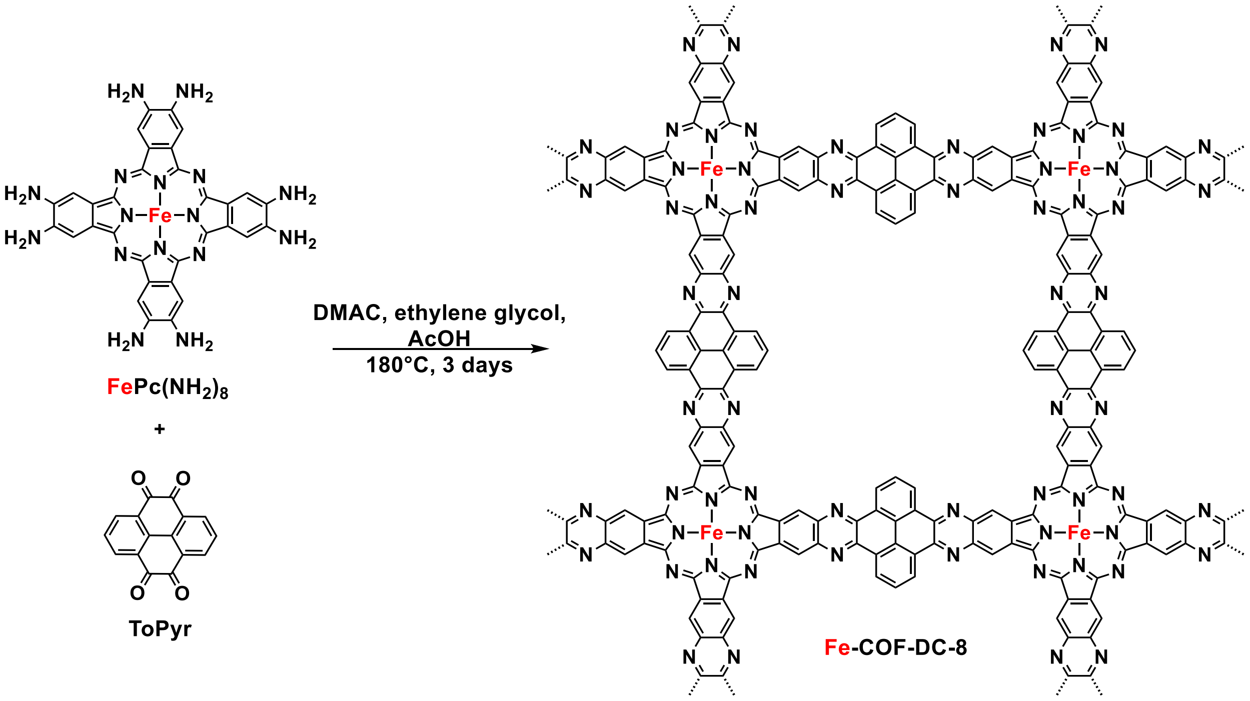

Scheme S6: Method of preparing Fe-COF-DC-8 from precursor components.

Optimized synthesis of Fe-COF-DC-8

To prepare Fe-COF-DC-8, a 15 mL pressure vessel was charged with 32.4 mg (0.033 mmol, 1 eq.) of $FePc(NH_2)_8$-8HCl. A solution of 8.48 mL NMP, and 0.097 mL of PTSA (3.5 M) was degassed using Ar. After degassing, the solution was added to the vessel followed by 17.5 mg (0.066 mmol, 2 eq.) of pyrenetetraone. The reaction was degassed with Ar for an additional 3 min before being sealed and sonicated for 30 mins. The reaction was heated to 150°C in an oil bath with stirring for 3 days. The reaction was allowed to cool to room temperature before being poured into acetone and centrifuged. The solids were washed with acetone (20 mL × 3), DI H_2O (25 mL × 3), and acetone (25 mL × 5). The solid was dried overnight on a high vacuum pump.

The above optimized synthetic procedure for Fe-COF-DC-8 was achieved by testing various solvent systems and acid promoters see in **Figure S1** and **Table S2**.

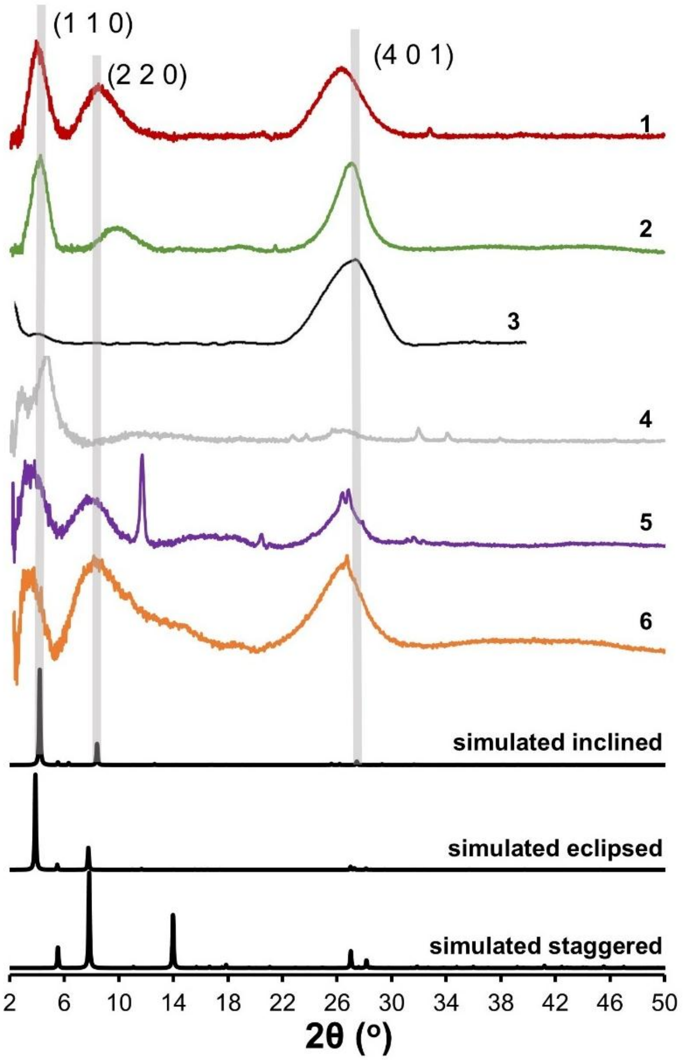

Figure S1. PXRD analysis of products from different reaction conditions for the synthesis of Fe-COF-DC-8. Powder patterns are plotted against the computationally modeled eclipsed stacking structure. Conditions are summarized in **Table S2**. Note: weak peaks above 30° in trial 1 are due to impurities in Vaseline used to adhere COF to the PXRD plate.

	Precursor Identity	[Prec.] mM	Prec. mass (mg)	Solvent	Additive	Additive: Prec.	Rxn Time (days)	Temp (°C)	Yield (%)
1	$FePc(NH_2)_8$ -HCl	30.3	100.9	DMAc: Ethylene glycol (1:1)	AcOH	116.7	3	180	100
2	$FePc(NH_2)_8$ -HCl	3.9	32.4	NMP	PTSA	21.2	3	150	31
3	$FePc(NH_2)_8$	14.5	20.0	NMP	PTSA	20	3	185	*
4	$FePc(NH_2)_8$ -HCl	*	10.0	PEG-400	none	N/A	3	170	*
5	$FePc(NH_2)_8$ -HCl	*	10.0	NMP	H_2SO_4	163.8	3	170	*
6	$FePc(NH_2)_8$ -HCl	*	10.0	NMP	none	N/A	3	170	*

Table S2. Conditions used to synthesize Fe-COF-DC-8.
Abbreviations: DMAc: Dimethylacetamide, NMP: N-methyl-2-pyrrolidone, PEG-400: polyethylene glycol, AcOH: glacial acetic acid, PTSA: *para*-toluenesulfonic acid, H_2SO_4: sulfuric acid. ***unknown**

Optimized synthesis of Co-COF-DC-8

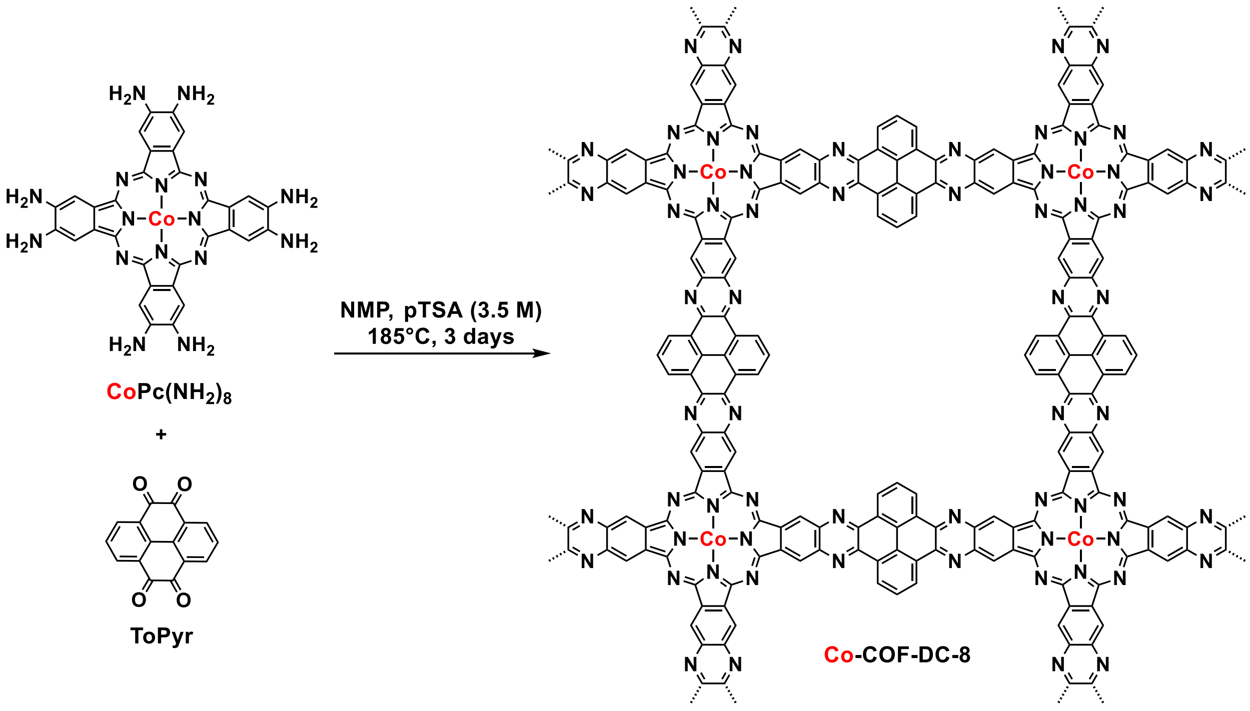

Scheme S7. Synthetic scheme detailing the reaction leading to the formation of Co-COF-DC-8 from the molecular precursors.

To prepare Co-COF-DC-8, a 15 mL pressure vessel was charged with 22.1 mg (0.023 mmol, 1 eq.) of $CoPc(NH_2)_8$-8HCl. 1.61 mL NMP was degassed using Ar. 0.45 mL pTSA (3.5 M) was degassed using Ar. After degassing, the NMP was added to the vessel followed by 11.8 mg (0.045 mmol, 2 eq.) of pyrenetetraone. The reaction was degassed with Ar for an additional 10 min before being sealed and sonicated for 10 mins. The reaction was heated to 185°C in an oil bath with stirring for 3 days. The reaction was allowed to cool to room temperature before being poured into acetone and centrifuged. The solids were washed with acetone (20 mL × 3), DI H_2O (25 mL × 3), and acetone (25 mL × 5). The solid was dried overnight on a high vacuum pump.

Optimized synthesis of Ni-COF-DC-8

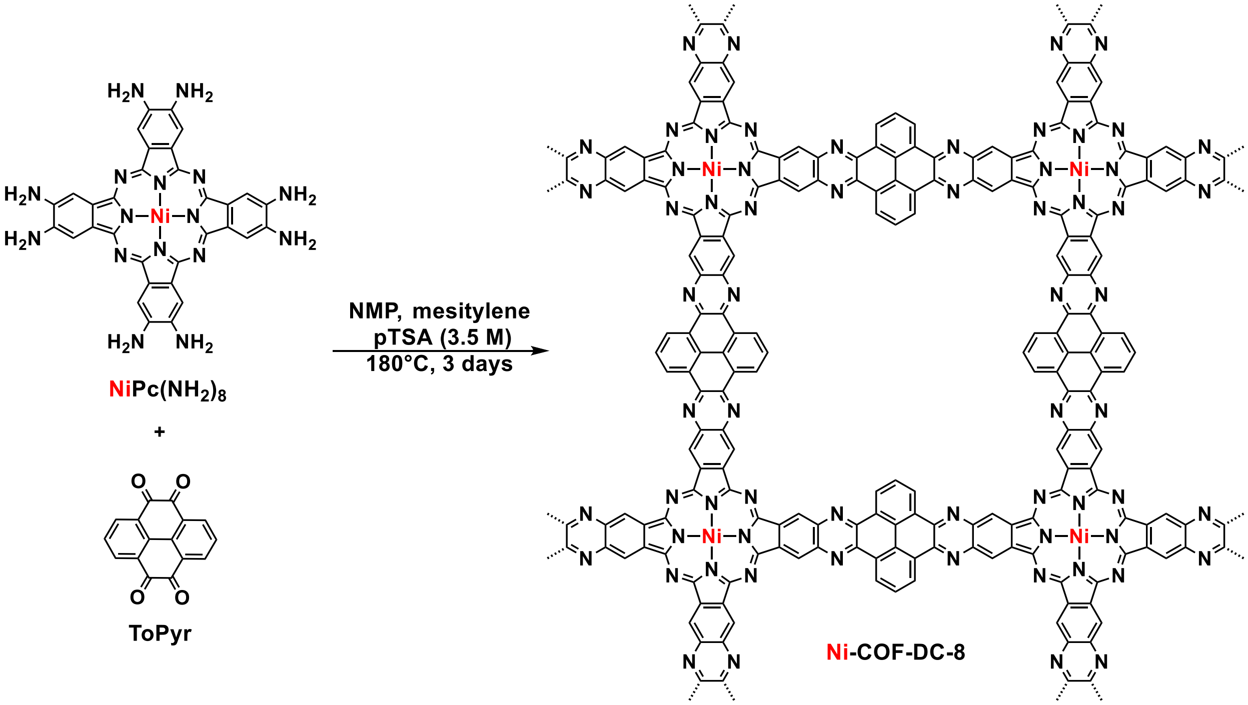

Scheme S8. Synthetic scheme detailing the reaction leading to the formation of Ni-COF-DC-8 from the molecular precursors.

To prepare Ni-COF-DC-8, a 20 mL Teflon liner for autoclave reactor vessel was charged with 60.2 mg (0.087 mmol, 1 eq.) of $NiPc(NH_2)_8$. A mixture of 4.36 mL NMP and 2.18 mL mesitylene was degassed using Ar. 0.87 mL pTSA (3.5 M) was degassed using Ar. After degassing, the NMP: mesitylene mixture was added to the vessel. The $NiPc(NH_2)_8$ solution was sonicated to dissolve the precursor. 45.7 mg (0.174 mmol, 2 eq.) of pyrenetetraone was added and further sonicated.

Degassed pTSA acid additive was added. The reaction was degassed with Ar for an additional 10 min before being sealed and sonicated for 30 mins. The reaction was heated to 180°C in an oven for 3 days. The reaction was allowed to cool to room temperature before being poured into acetone and centrifuged. The solids were washed with acetone (45 mL ×1), NMP (45 mL × 2), DI H_2O (45 mL × 1),and acetone (45 mL × 3). The solid was dried overnight on a high vacuum pump. Before further analysis and application, the materials were soaked in EtOH for 3 days at 65°C. The EtOH was changed every 8 h. After activation, the material was dried for 72 h under vacuum at room temperature.

The above optimized synthetic procedure for Ni-COF-DC-8 was achieved by testing various solvent systems and acid promoters see in **Figure S2** and **Table S3**.

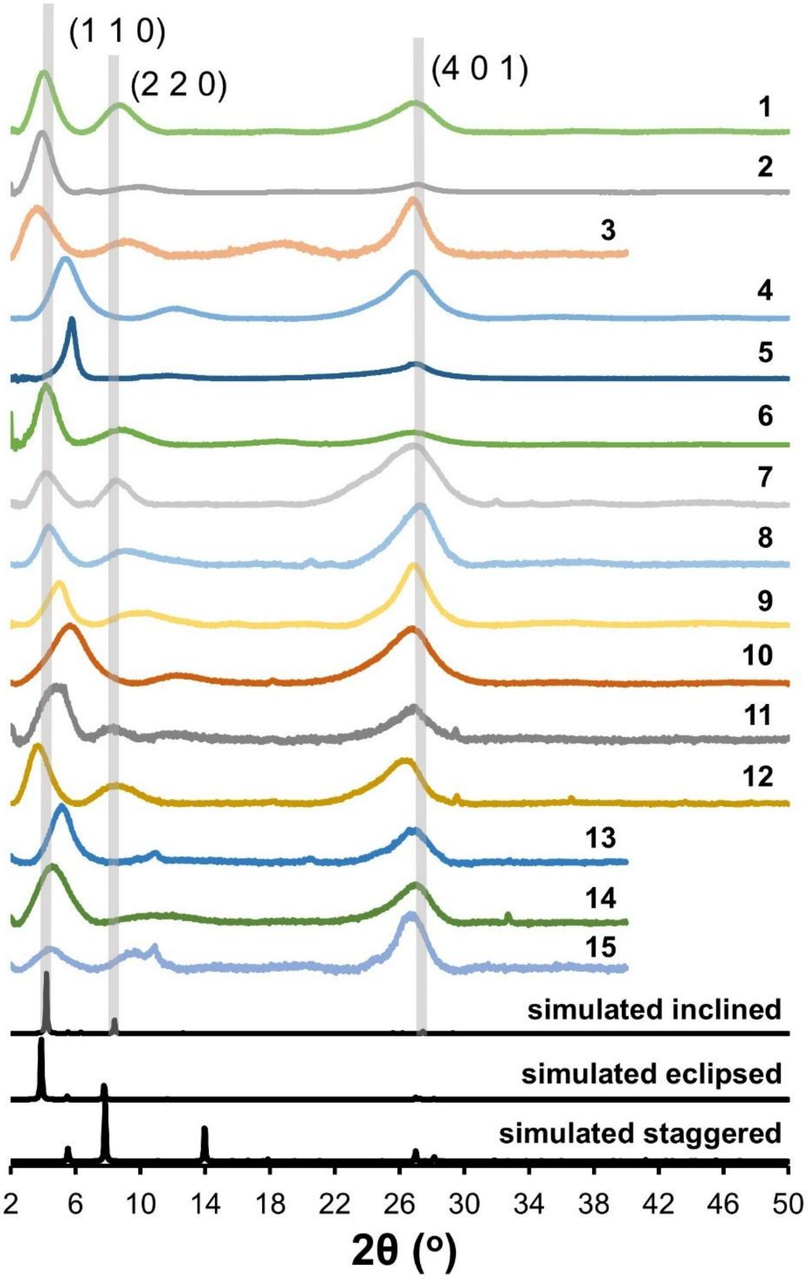

Figure S2. PXRD analysis of different reaction conditions for the synthesis of Ni-COF-DC-8. Powder patterns are plotted against computationally modeled structures. Conditions are summarized in **Table S3**.

	Precursor Identity	[Prec.] mM	Prec. mass (mg)	Solvent	Additive	Additive: Prec.	Rxn Time (days)	Temp (°C)	Yield (%)
1	$NiPc(NH_2)_8$	13.33	60.2	NMP: mesitylene (2:1)	PTSA	35.0	3	180	98
2	$NiPc(NH_2)_8$	13.33	20.7	NMP: mesitylene (2:1)	PTSA	35.0	3	180	78
3	$NiPc(NH_2)_8$	14.97	20.7	NMP: DCB (1:1)	PTSA	20	3	150	*
4	$NiPc(NH_2)_8$	1.50	12.0	DMAc: DCB (1:1)	H_2SO_4	9.2	10	202	32
5	$NiPc(NH_2)_8$	1.50	31.3	DMAc: DCB (1:1)	H_2SO_4	9.0	10	180	54
6	$NiPc(NH_2)_8$ -HCl	19.0	12.1	NMP: mesitylene (2:1)	PTSA	24.6	3	180	31
7	$NiPc(NH_2)_8$	13.33	20	NMP: mesitylene (2:1)	AcOH	35.0	3	180	72
8	$NiPc(NH_2)_8$	13.33	20	NMP: mesitylene (2:1)	H_2SO_4	35.0	3	180	97
9	$NiPc(NH_2)_8$	13.33	20	NMP: mesitylene (2:1)	MsOH	35.0	3	180	90
10	$NiPc(NH_2)_8$	14.97	20.7	DMAc: mesitylene (1:1)	H_2SO_4	17.8	10	180	*
11	$NiPc(NH_2)_8$	14.97	20.7	DMAc: DCB (1:1)	H_2SO_4	17.8	10	180	*
12	$NiPc(NH_2)_8$	14.97	20.7	DMSO	H_2SO_4	17.8	10	180	*
13	$NiPc(NH_2)_8$	14.97	20.7	NMP: DCB (1:1)	H_2SO_4	20.0	3	150	*
14	$NiPc(NH_2)_8$	14.97	20.7	NMP: DCB (1:1)	TCA	20.0	3	150	*
15	$NiPc(NH_2)_8$	14.97	20.7	NMP: DCB (1:1)	PPA	20.0	3	150	*

Table S3. Conditions used to synthesize Ni-COF-DC-8.
Abbreviations: NMP: N-methyl-2-pyrrolidone, DCB: 1,2-dichlorobenzene, DMAc: Dimethylacetamide, DMSO: Dimethyl sulfoxide, PTSA: *para*-toluenesulfonic acid, H_2SO_4: sulfuric acid, AcOH: glacial acetic acid, MsOH: methanesulfonic acid, TCA: trichloroacetic acid, PPA: polyphosphoric acid.

Optimized synthesis of Cu-COF-DC-8

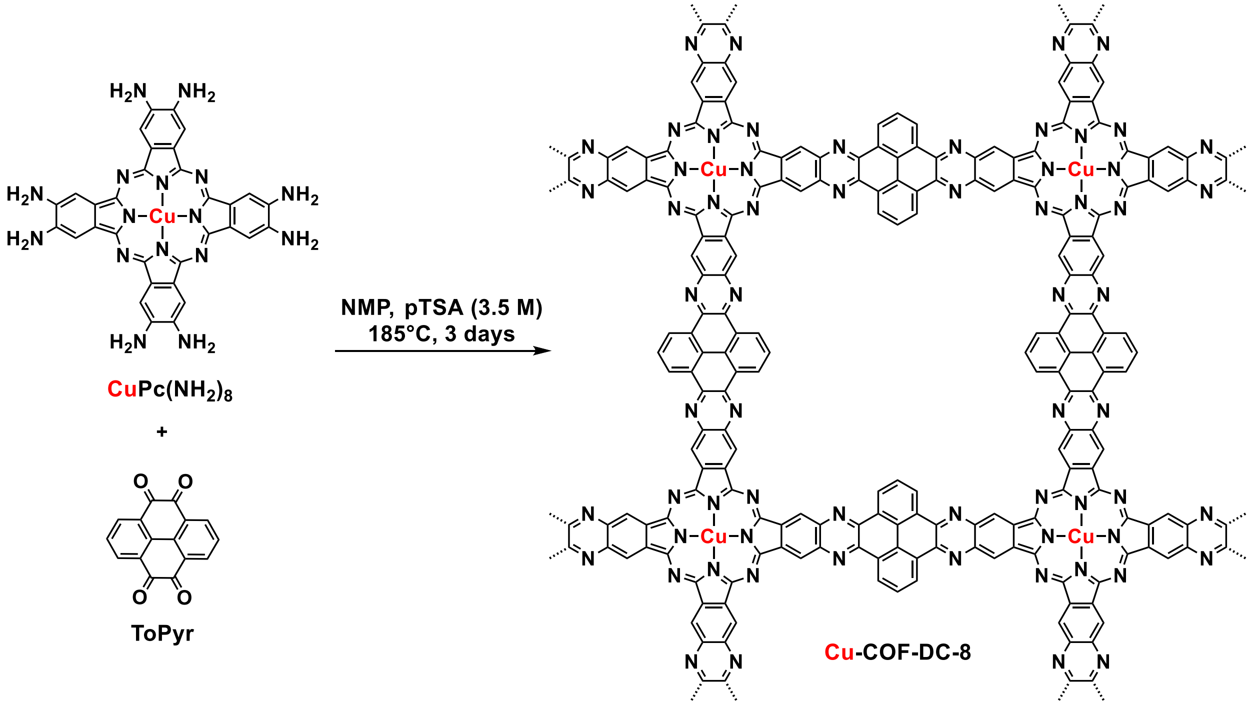

Scheme S9. Synthetic scheme detailing the reaction leading to the formation of Cu-COF-DC-8 from the molecular precursors.

To prepare Cu-COF-DC-8, a 15 mL pressure vessel was charged with 58.0 mg (0.059 mmol, 1 eq.) of $CuPc(NH_2)_8$-8HCl. 4.75 mL NMP was degassed using Ar. 1.16 mL pTSA (3.5 M) was degassed using Ar. After degassing, the NMP was added to the vessel followed by 30.8 mg (0.117 mmol, 2 eq.) of pyrenetetraone. The reaction was degassed with Ar for an additional 10 min before being sealed and sonicated for 10 mins. The reaction was heated to 180°C in an oil bath with stirring for 3 days. The reaction was allowed to cool to room temperature before being poured into acetone and centrifuged. The solids were washed with acetone (20 mL × 3), DI H_2O (20 mL × 2), and acetone (20 mL × 3). The solid was dried overnight on a high vacuum pump.

The above optimized synthetic procedure for Cu-COF-DC-8 was achieved by testing various solvent systems and acid promoters see in **Figure S3** and **Table S4**.

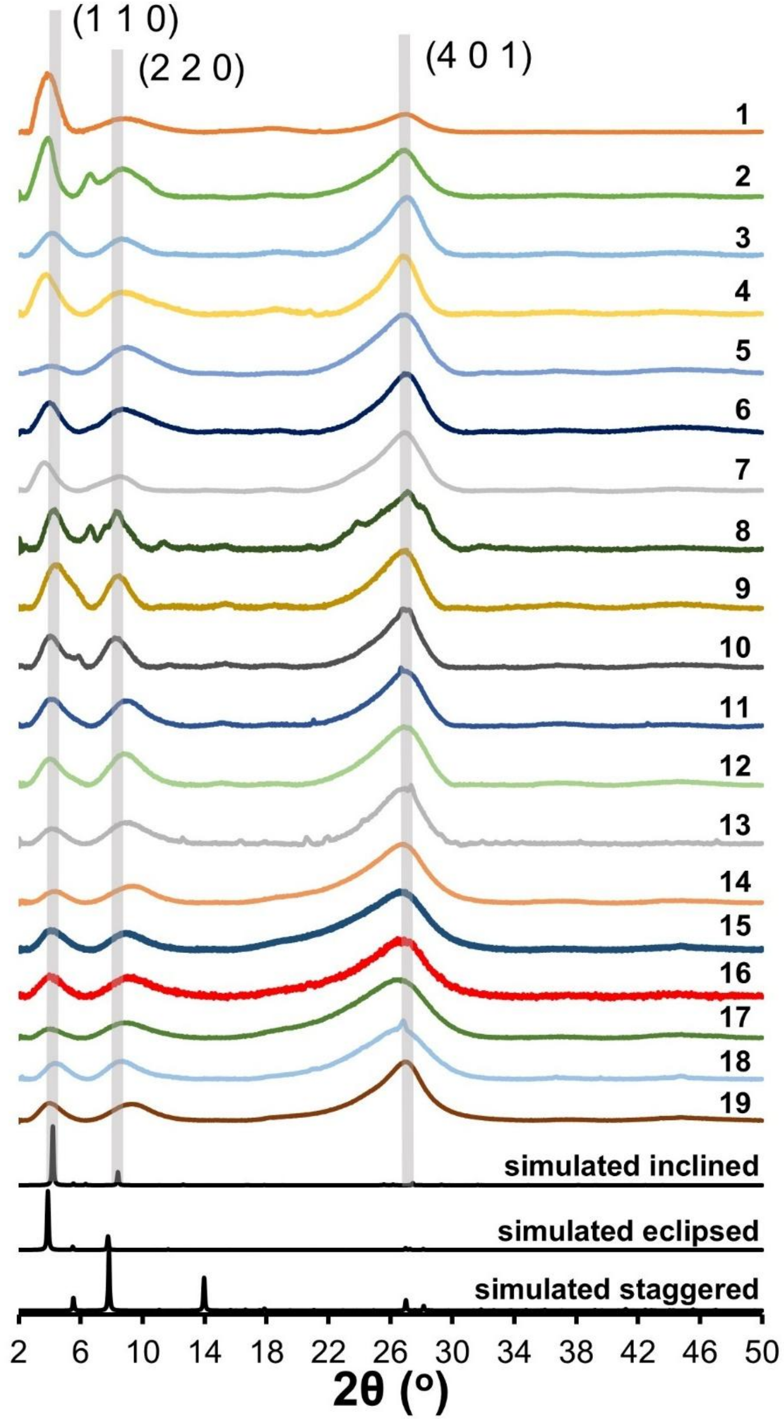

Figure S3. PXRD analysis of different reaction conditions for the synthesis of Cu-COF-DC-8. Powder patterns are plotted against computationally modeled structures. Conditions are summarized in **Table S4**.

	Precursor Identity	[Prec.] mM	Prec. mass (mg)	Solvent	Additive	Additive: Prec.	Rxn Time (days)	Temp (°C)	Yield (%)
1	$CuPc(NH_2)_8$ -HCl	12.4	58.0	NMP	PTSA	67.2	3	180	58
2	$CuPc(NH_2)_8$	17.4	21.5	NMP	PTSA	1.1	3	180	88
3	$CuPc(NH_2)_8$	17.4	25.0	NMP	PTSA	1.1	3	180	100
4	$CuPc(NH_2)_8$	17.4	10.0	NMP	PTSA	1.1	3	180	100
5	$CuPc(NH_2)_8$	17.4	10.0	NMP	PTSA	35.0	3	180	100
6	$CuPc(NH_2)_8$	17.4	10.0	NMP	PTSA	6.9	3	180	98
7	$CuPc(NH_2)_8$	17.4	10.0	NMP	PTSA	1.1	3	180	98
8	$CuPc(NH_2)_8$	17.4	10.0	NMP: mesitylene (2:1)	PTSA	1.1	3	180	87
9	$CuPc(NH_2)_8$	19.7	8.0	DMAc: ethylene glycol (5.1:1)	AcOH	116.8	3	180	51
10	$CuPc(NH_2)_8$	13.4	8.0	DMAc: ethylene glycol (5.1:1)	AcOH	116.8	3	180	71
11	$CuPc(NH_2)_8$	16.7	10.0	NMP: mesitylene (2:1)	PTSA	35.0	3	180	73
12	$CuPc(NH_2)_8$	17.4	10.0	NMP	PTSA	1.1	3	180	76
13	$CuPc(NH_2)_8$	17.4	13.3	NMP	PTSA	1.1	3	200	97
14	$CuPc(NH_2)_8$ -HCl	5.0	9.4	NMP	PTSA	1.1	3	200	98
15	$CuPc(NH_2)_8$	17.4	11.6	NMP: DCB (1:1)	PTSA	1.1	3	200	75
16	$CuPc(NH_2)_8$	17.4	11.1	NMP	PTSA	1.1	3	200	82
17	$CuPc(NH_2)_8$	17.4	17.7	NMP	PTSA	6.9	3	200	96
18	$CuPc(NH_2)_8$	17.4	20.0	NMP	AcOH	49.1	3	200	88
19	$CuPc(NH_2)_8$	17.4	20.0	NMP	PTSA	49.1	3	200	61

Table S4. Conditions used to synthesize Cu-COF-DC-8.
Abbreviations: NMP: N-methyl-2-pyrrolidone, DMAc: Dimethylacetamide, DCB: 1,2-dichlorobenzene, PTSA: *para*-toluenesulfonic acid, AcOH: glacial acetic acid.

Summarization of optimized syntheses of M-COF-DC-8 (M=Fe, Co, Ni, and Cu)

M-COF-DC-8	Precursor Identity	[Prec.] mM	Prec. mass (mg)	Solvent	Additive	Additive : Prec.	Rxn Time (days)	Temp (°C)	Yield (%)
Fe	$FePc(NH_2)_8$-HCl	3.9	32.4	NMP	PTSA	21.2	3	150	31
Co	$CoPc(NH_2)_8$-HCl	14.0	22.1	NMP	PTSA	70.0	3	185	N/A
Ni	$NiPc(NH_2)_8$	13.33	60.2	NMP: mesitylene (2:1)	PTSA	35.0	3	180	98
Cu	$CuPc(NH_2)_8$-HCl	12.4	58.0	NMP	PTSA	67.2	3	180	58

Table S5. Summary table of M-COF-DC-8 (M=Fe, Co, Ni, and Cu) synthetic conditions.

The synthetic condition for each COF was optimized through trials detailed in the above sections. There were a few key synthetic differences across the COF suite. First, different precursor forms were used. For instance, the Fe-, Co, and Cu-COF-DC-8 materials were formed from the HCl salt protected precursor, while Ni-COF-DC-8 was formed using the free base. It was found that $NiPc(NH_2)_8$ was less susceptible to degradation compared to the other three precursor derivatives, likely due to its more stable diamagnetic center. This stability allowed for the Ni-based precursor to be used in the free amine state while the other derivatives were used in the salt form to reduce amine reactivity. Second, different solvent systems (NMP v NMP/mesitylene) and precursor concentrations were used due to the different solubility requirements for optimal COF formation. It has been shown in literature that metallophthalocyanine monomers can exhibit varying solubility due to different dimerization constants, necessitating various conditions for optimal COF formation.[4]

(a) M-COF-DC-8	(110) 2θ peak (°)	(220) 2θ peak (°)	(401) 2θ peak (°)
Fe	4.26	9.90	27.02
Co	3.80	8.52	27.16
Ni	4.06	8.72	27.02
Cu	3.82	8.88	27.10

(b) Simulated Packing Pattern	(110) 2θ peak (°)	(220) 2θ peak (°)	(401) 2θ peak (°)
Inclined	4.20	8.40	27.44

(c) Simulated Packing Pattern	(100) 2θ peak (°)	(200) 2θ peak (°)	(001) 2θ peak (°)
Eclipsed	3.90	7.82	26.98
Staggered	N/A	7.82	27.00

Table S6. (a) Comparison of experimental PXRD peak positions for M-COF-DC-8 (M=Fe, Co, Ni, Cu) corresponding to the (110), (220), and (401) lattice planes. (**b**) Comparison of PXRD peak positions for the simulated inclined COF packing pattern. (**c**) Comparison of the PXRD peak positions for the simulated eclipsed and staggered COF packing patterns.

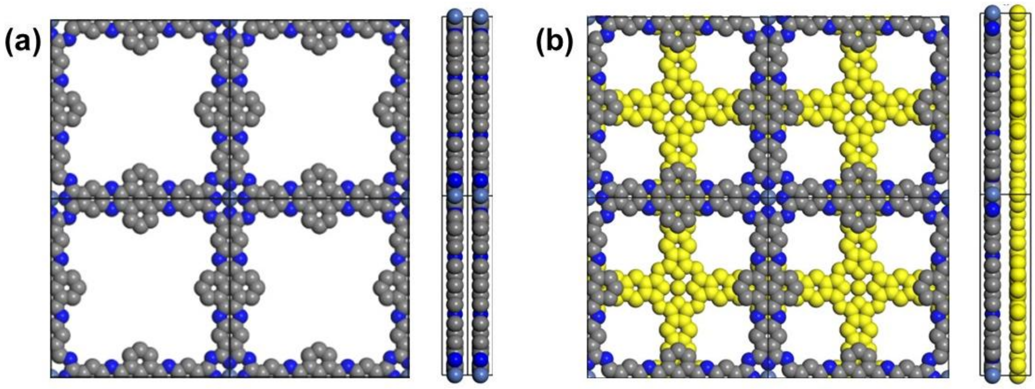

Figure S4. Simulated crystal structures of COF-DC-8 consisting of 2D sheets with (**a**) eclipsed and (**b**) staggered packing patterns.

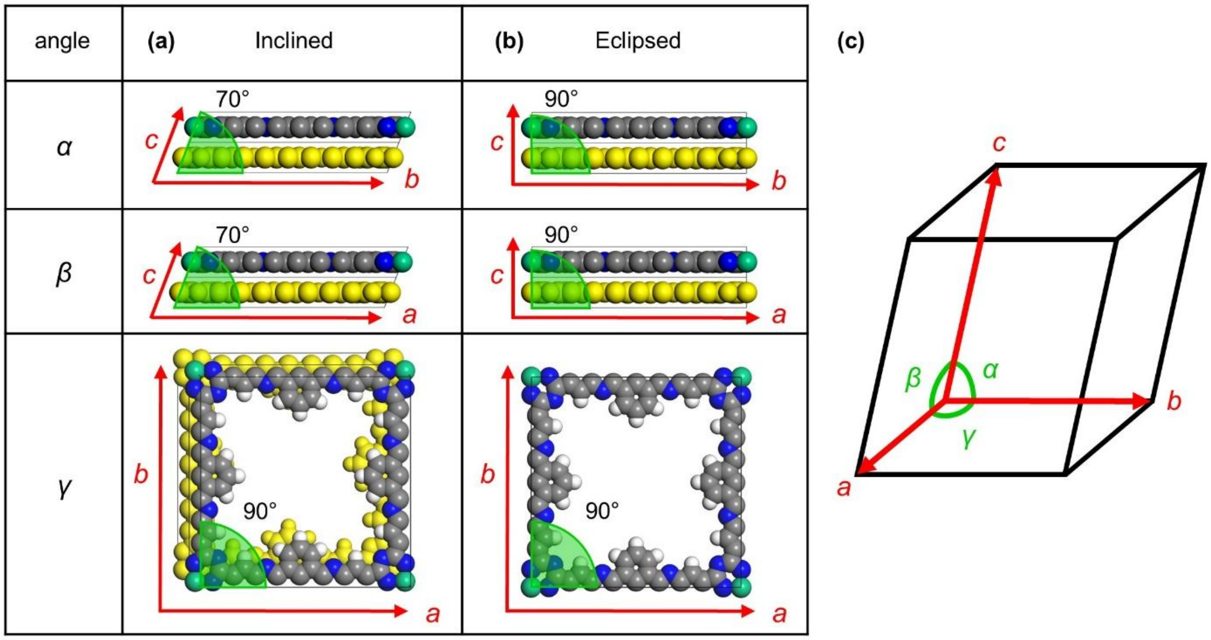

Figure S5. Simulated crystal structures of M-COF-DC-8 consisting of 2D sheets with (**a**) inclined and (**b**) eclipsed packing patterns from different viewpoints to better visualize the *α, β,* and *γ* angles resulting in different crystal symmetries. (**c**) Unit cell in three dimensions with labels for edges and angles.

Table S7. Unit cell parameters (distances for a, b, and c lattice parameters) for inclined and eclipsed packing pattern.

Simulated Packing Pattern	a (nm)	b (nm)	c (Å)
Inclined	2.26	2.26	3.3
Eclipsed	2.27	2.27	3.3

IV. Elemental analysis of M-COF-DC-8 materials

Metal content was determined by ICP-MS. Samples were dissolved in a weak piranha solution (H_2SO_4 and H_2O_2, 10:1 vol.) and the resulting brown solution was further diluted 10:1 in DI H_2O. These samples were analyzed in-house by ICP-MS. Analysis of the light elements H, N, and C was accomplished using combustion analysis from Atlantic Microlabs. Duplicate values indicate a measurement performed twice on the same sample.

Element	Theory	Found	Found	Average	Diff.
C	71.7	58.1	57.9	58.0	13.7
H	1.1	2.9	2.8	2.9	-1.8
N	20.9	15.7	15.6	15.7	5.2
Fe	6.3	6.9	-	-	-0.6

Table S8. Elemental analysis of Fe-COF-DC-8 showed that the composition of the synthesized material was in good agreement with theoretical values.

Element	Theory	Found	Found	Average	Diff.
C	71.8	61.6	61.4	61.5	10.3
H	1.1	3.6	3.5	3.5	-2.5
N	20.9	16.0	16.1	16.0	4.9
Co	6.3	5.1	-	-	1.2

Table S9. Elemental analysis of Co-COF-DC-8 showed that the composition of the synthesized material was in good agreement with theoretical values.

Element	Theory	Found	Found	Average	Diff.
C	71.7	64.6	64.7	64.6	7.1
H	1.1	3.2	3.3	3.2	-2.1
N	20.9	17.3	17.4	17.3	3.6
Ni	6.3	8.4	-	-	-2.1

Table S10. Elemental analysis of Ni-COF-DC-8 showed that the composition of the synthesized material was in good agreement with theoretical values and the previous report of this material.[5]

Element	Theory	Found	Found	Average	Diff.
C	71.8	61.5	61.4	61.4	10.3
H	1.1	3.4	3.3	3.4	-2.3
N	20.9	17.7	17.6	17.7	3.2
Cu	6.3	4.7	-	-	1.6

Table S11. Elemental analysis of Cu-COF-DC-8 showed that the composition of the synthesized material was in good agreement with theoretical values.

V. X-Ray Photoelectron Spectroscopy Analysis of Pristine M-COF-DC-8 Materials

XPS Survey Spectra

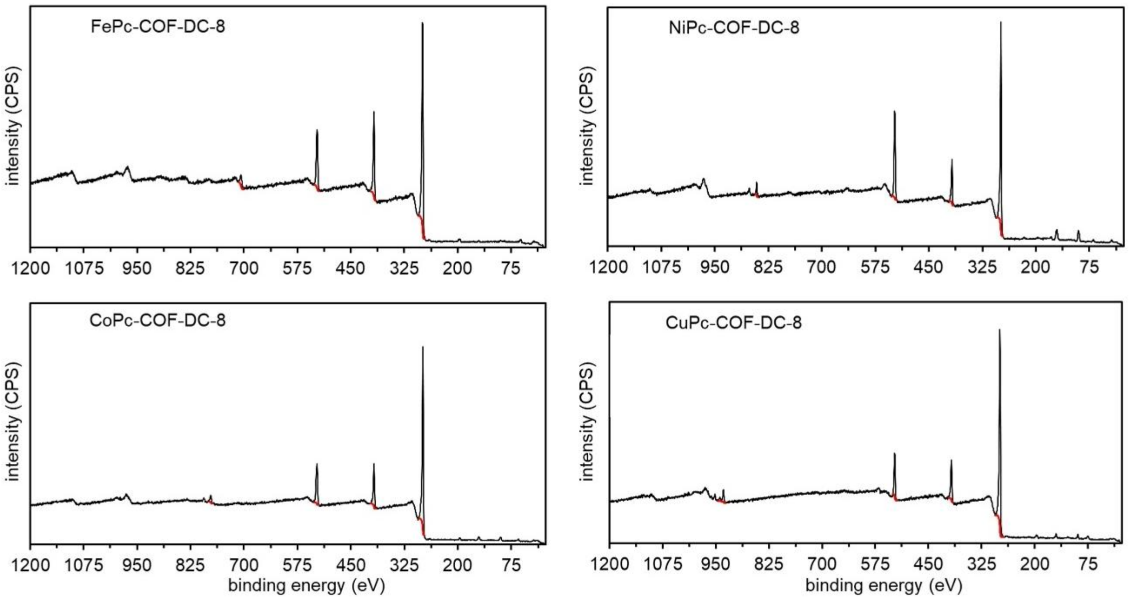

Figure S6. Survey spectra of the four M-COF-DC-8 characterized as pristine powders.

XPS Elemental Regions for Fe-COF-DC-8

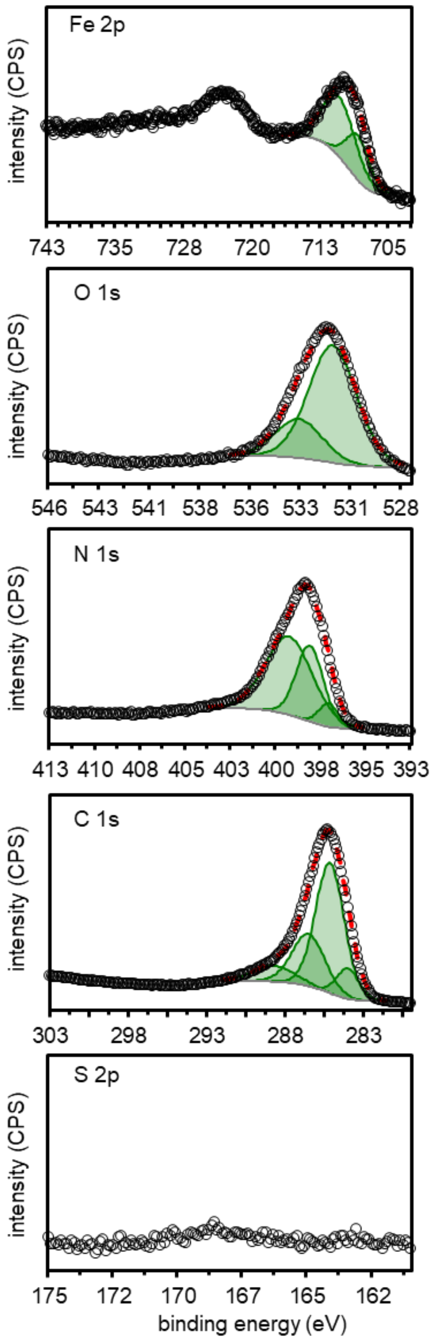

Figure S7. Elemental regions of Fe-COF-DC-8 in its pristine state. The emission regions matched the expected elemental content of the framework (Fe, N, C) and also included O species potentially coming from defects involving pyrene quinones or adsorbed water. The Fe 2p region was fit with two peaks which we assigned to mixed valency (Fe^{2+}/Fe^{3+}) within the framework.

XPS Elemental Regions for Co-COF-DC-8

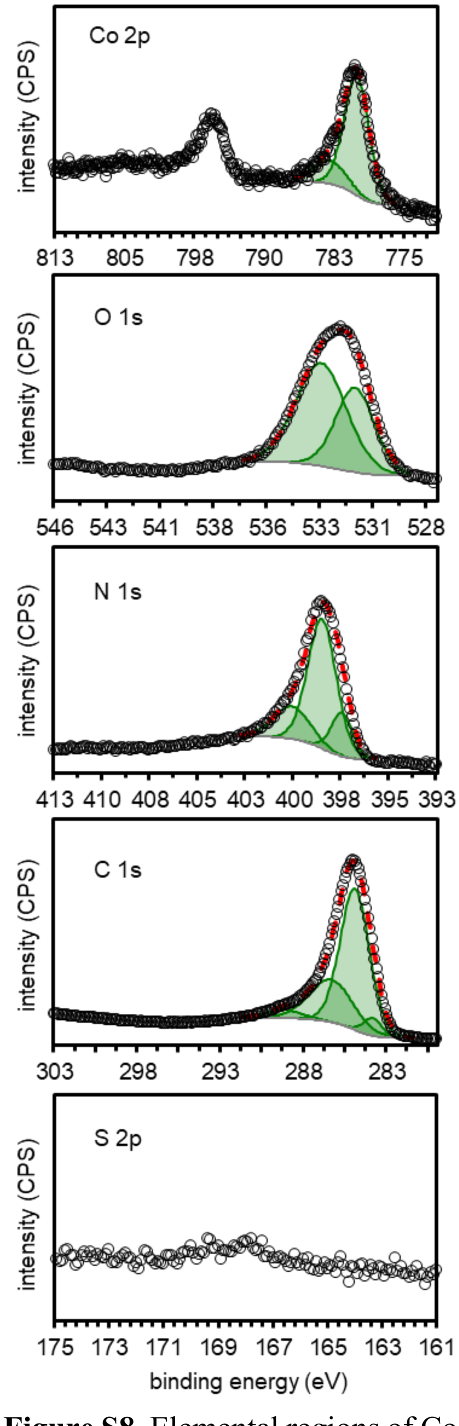

Figure S8. Elemental regions of Co-COF-DC-8 in its pristine state. The emission regions matched the expected elemental content of the framework (Co, N, C) and also included O species potentially coming from defects involving pyrene quinones or adsorbed water.

XPS Elemental Regions for Ni-COF-DC-8

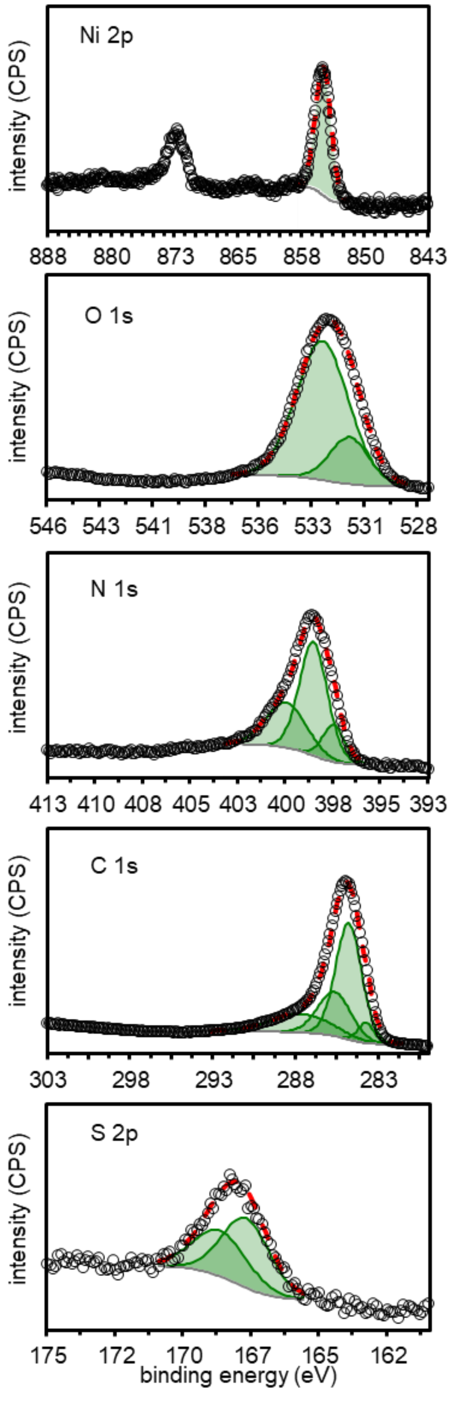

Figure S9. Elemental regions of Ni-COF-DC-8 in its pristine state. The emission regions matched the expected elemental content of the framework (Ni, N, C) and also included O species potentially coming from defects involving pyrene quinones or adsorbed water. The Ni-COF-DC-8 derivative showed a small quantity of S impurity.

XPS Elemental Regions for Cu-COF-DC-8

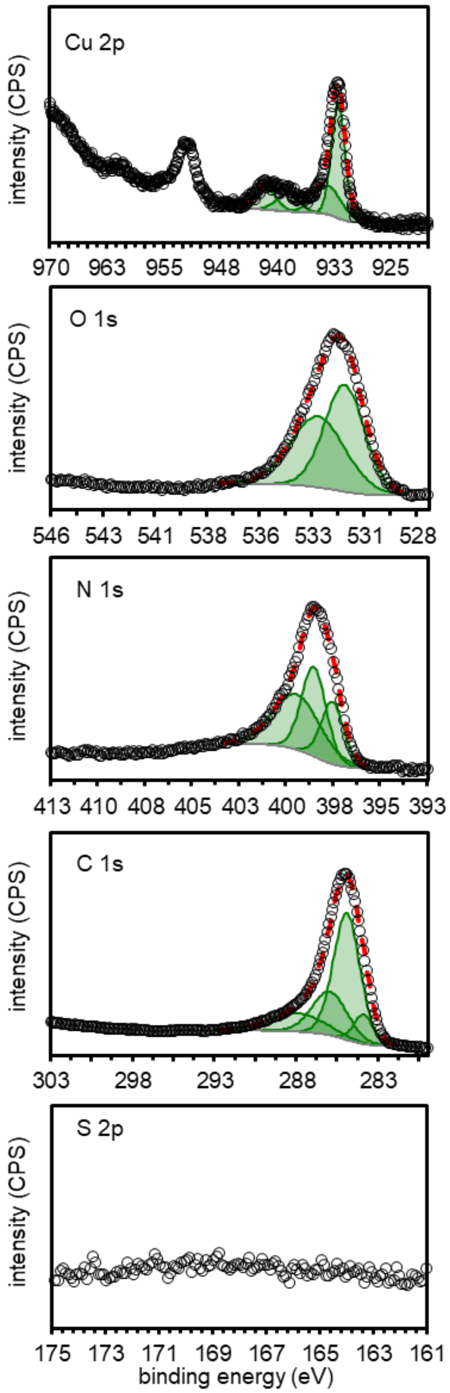

Figure S10. Elemental regions of Cu-COF-DC-8 in its pristine state. The emission regions matched the expected elemental content of the framework (Cu, N, C) and also included O species potentially coming from defects involving pyrene quinones or adsorbed water.

VI. DRIFTS Characterization of Pristine M-COF-DC-8 Materials

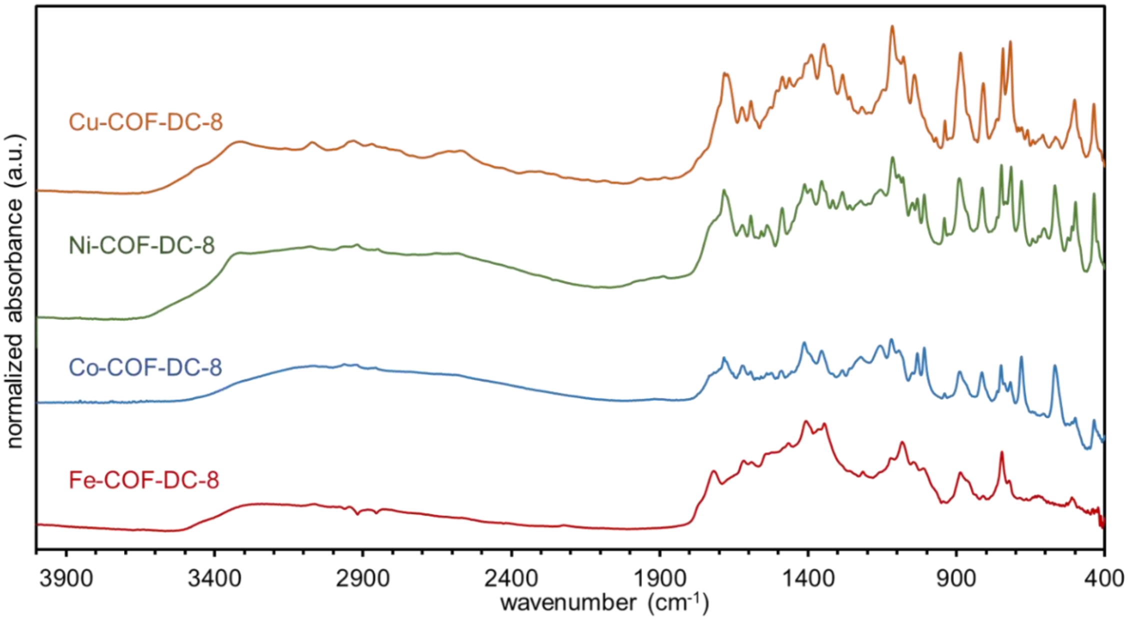

Figure S11. DRIFTS spectra of the pristine COF powders blended with KBr. Spectra were measured under an atmosphere of N_2 at 27°C. Before measurement, samples were dried at 100°C under N_2 for a minimum of 20 min until IR absorption profiles remained unchanged in subsequent spectra (taken 5 minutes apart).

VII. Electron Microscopy of M-COF-DC-8

SEM micrograph of M-COF-DC-8

Samples were prepared for SEM by mounting dried activated COF samples on double-sided carbon tape and gently pressing the powders into the tape with a glass microscope slide. The excess powder was blown off with a stream of N_2.

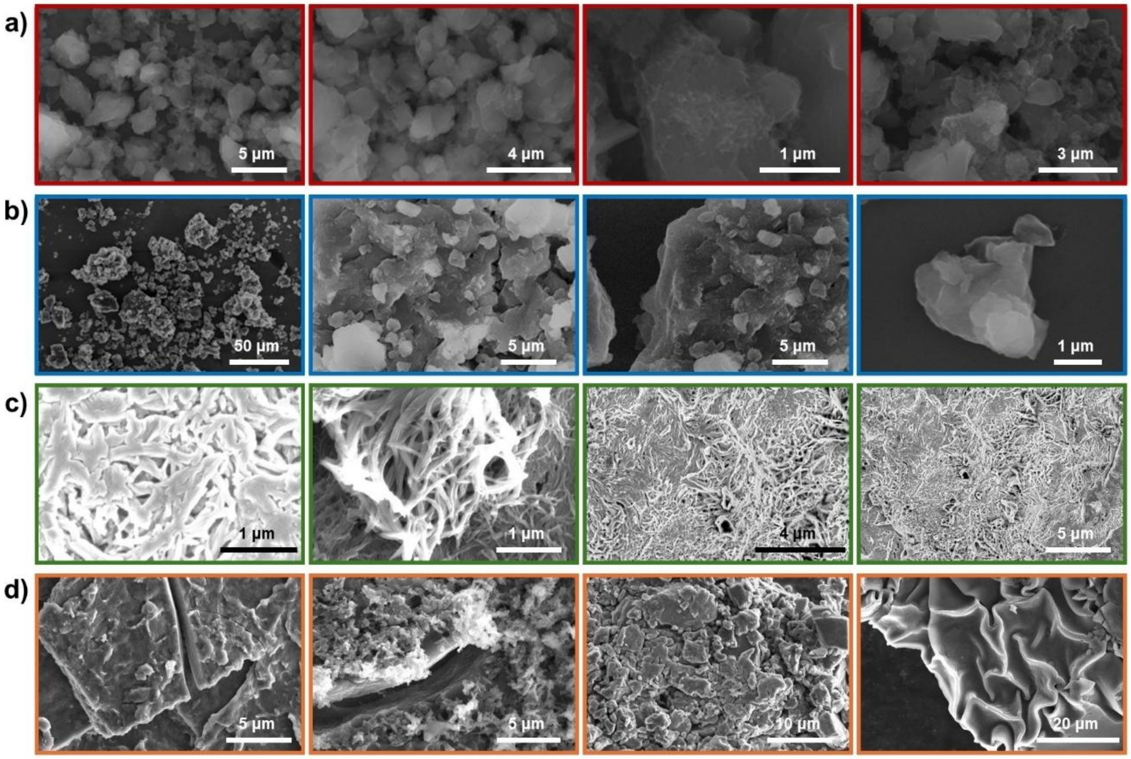

Figure S12. SEM images of (**a**) Fe-COF-DC-8, (**b**) Co-COF-DC-8, (**c**) Ni-COF-DC-8, (**d**) Cu-COF-DC-8. Images were obtained using an acceleration voltage of about 15.00, 10.00, 2.00, and 5.00 kV and a working distance of 4.1, 4.1, 4.0, and 4.1 mm for each respective COF.

Transmission Electron Microscopy of COF Materials

The limited stability of COFs towards the electron beam prevented further analysis by HRTEM and SAED to resolve specific stacking arrangements (eclipsed versus inclined).

a)

b)

c)

d)

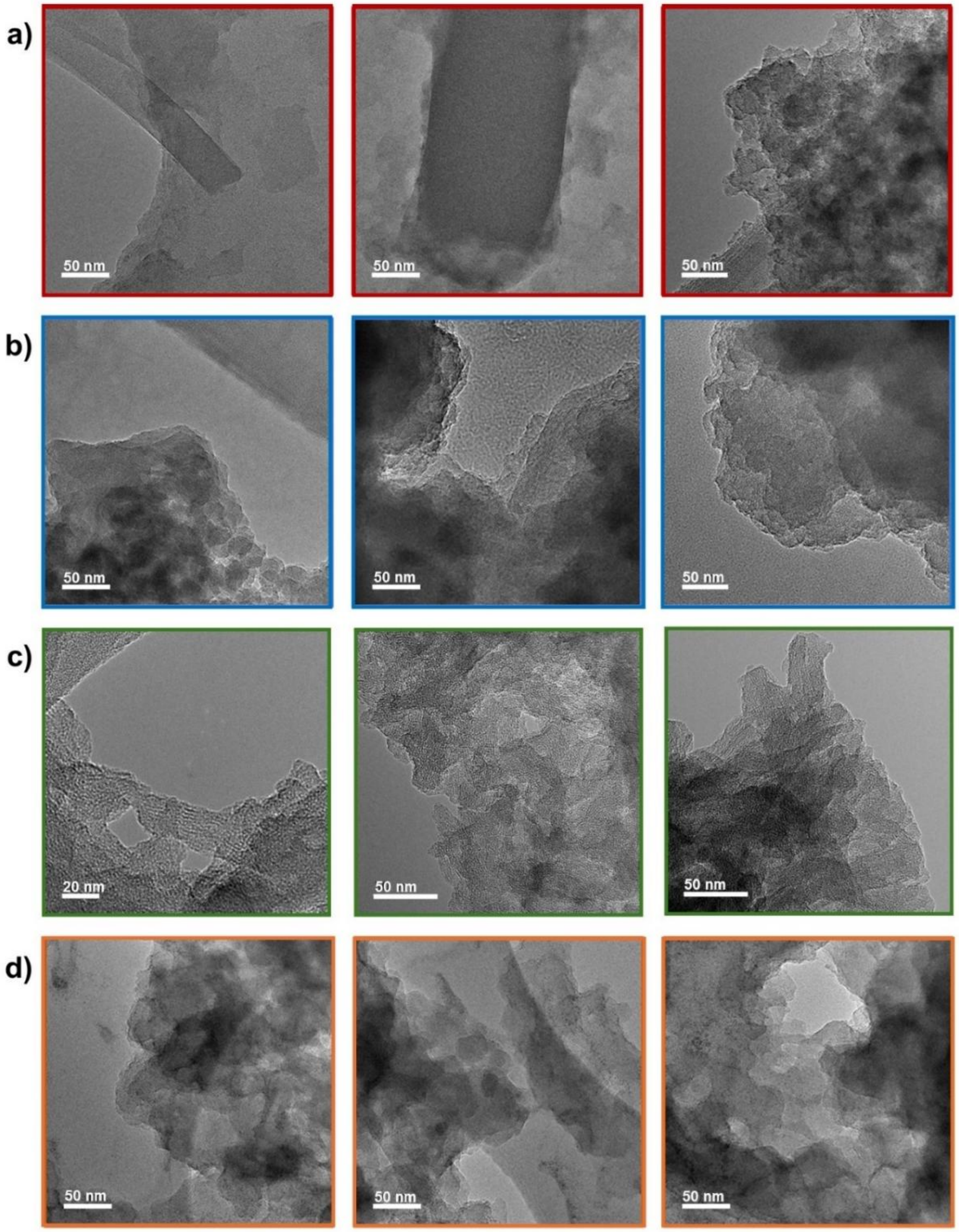

Figure S13. TEM micrographs of M-COF-DC-8 powders dropcast from a sonicated suspension in water. (**a**) Fe-COF-DC-8, (**a**) Co-COF-DC-8, (**a**) Ni-COF-DC-8, (**d**) Cu-COF-DC-8.

TEM micrographs of Fe-COF-DC-8 with a depiction of viewers' perspective on materials.

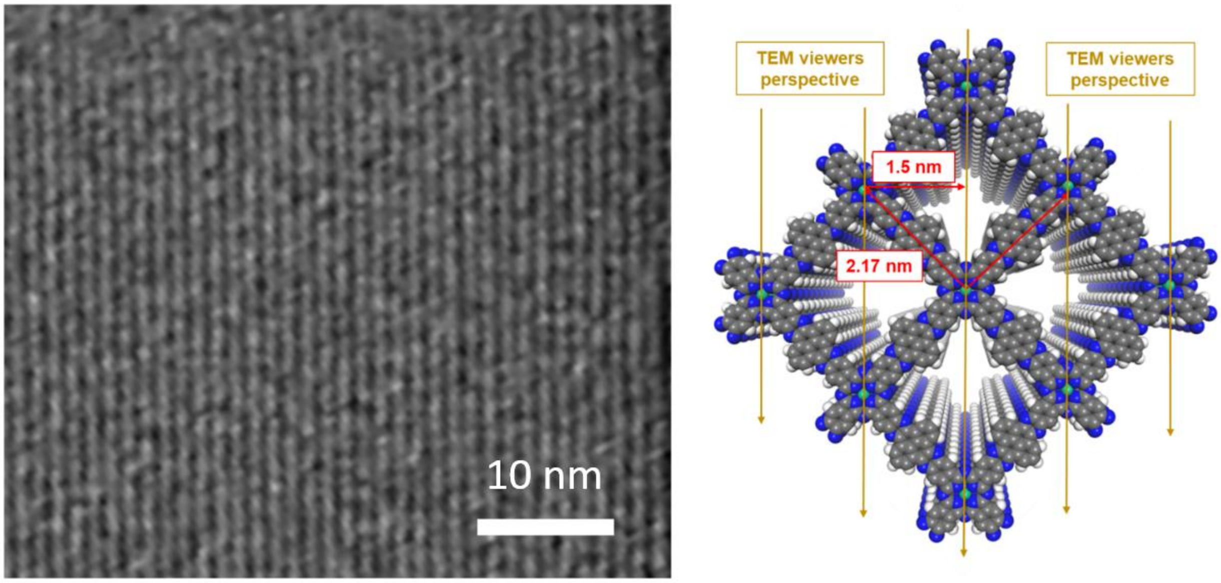

Figure S14. TEM image of a nanorod of Fe-COF-DC-8 (**left**). Interpretation of the striations observed (**right).**

VIII. Thermal Gravimetric Analysis of Pristine M-COF-DC-8 Materials

Thermal gravimetric analysis was conducted using a TGA Q5000 V3.17 Build 265 instrument with a 20°C/min ramping rate from 40°C to 840°C under N_2 atmosphere. The TGA profile of the Fe-, Co-, Ni-, and Cu-COF-DC-8 materials revealed 7, 2, 5, and 2% weight loss, respectively, before 180°C, likely due to the loss of small-molecules in the pores. By 540°C, 29, 21, 16, and 14% weight loss, respectively, was observed indicating the good thermal stability of the materials.

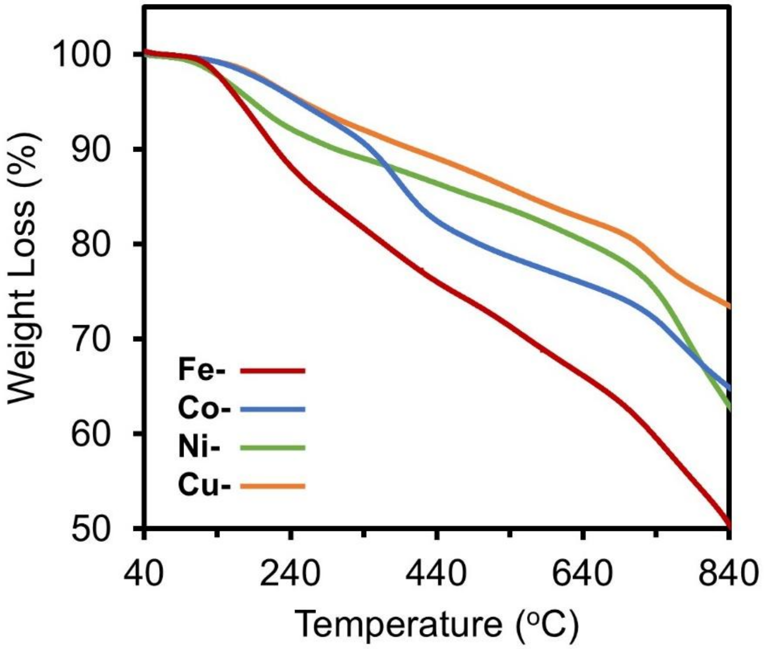

Figure S15. TGA curves of pristine M-COF-DC-8 materials under N_2 atmosphere.

IX. EPR of Pristine M-COF-DC-8 Materials

We performed EPR analysis to help elucidate the electronic structure of the metal centers and any radical character of the frameworks. To obtain the spectra, 1–2 mg of M-COF-DC-8 was placed into a quartz EPR tube and flushed with N_2 overnight. A spectrum was then obtained at 4.2 K by integrating over four filed sweeps from 600 G to 5000 G for Co-COF-DC-8, Ni-COF-DC-8, and Cu-COF-DC-8, and from 9.0–1.5 g for Fe-COF-DC-8.

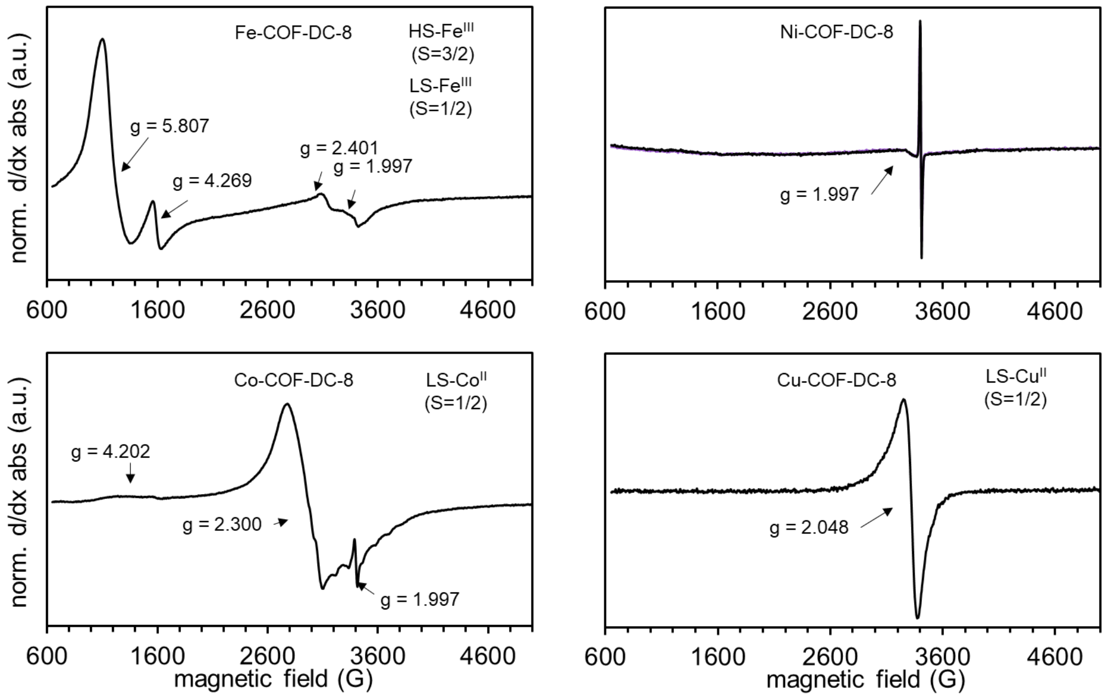

Figure S16. EPR spectra of the pristine M-COF-DC-8 materials at 4.2 K after degassing for 1 h with N_2.

X. Specific Surface Area of the M-COF-DC-8 Materials

Gas adsorption isotherms were recorded using N_2 as the adsorbate at 77 K. Before measurement, M-COF-DC-8 (M=Fe, Co) materials were activated by solvent exchange with EtOH at 65°C changing the solvent every 2–12 h. M-COF-DC-8 (M=Ni, Cu) materials were activated by solvent exchange with NMP at 65°C changing the solvent twice within 24 h followed by solvent exchange with acetone at 65°C changing the solvent every 2–12 h for 3 days. After solvent exchange, the materials were dried under vacuum at room temperature for 12 h. Once dry, samples were loaded into sample tubes (tubes were pre-dried in an oven at 220°C) and degassed at 75 mTorr and various temperatures: 120°C for an additional 48 h for M-COF-DC-8 (M=Fe, Co) and 160°C for an additional 18 h for M-COF-DC-8 (M=Ni, Cu).

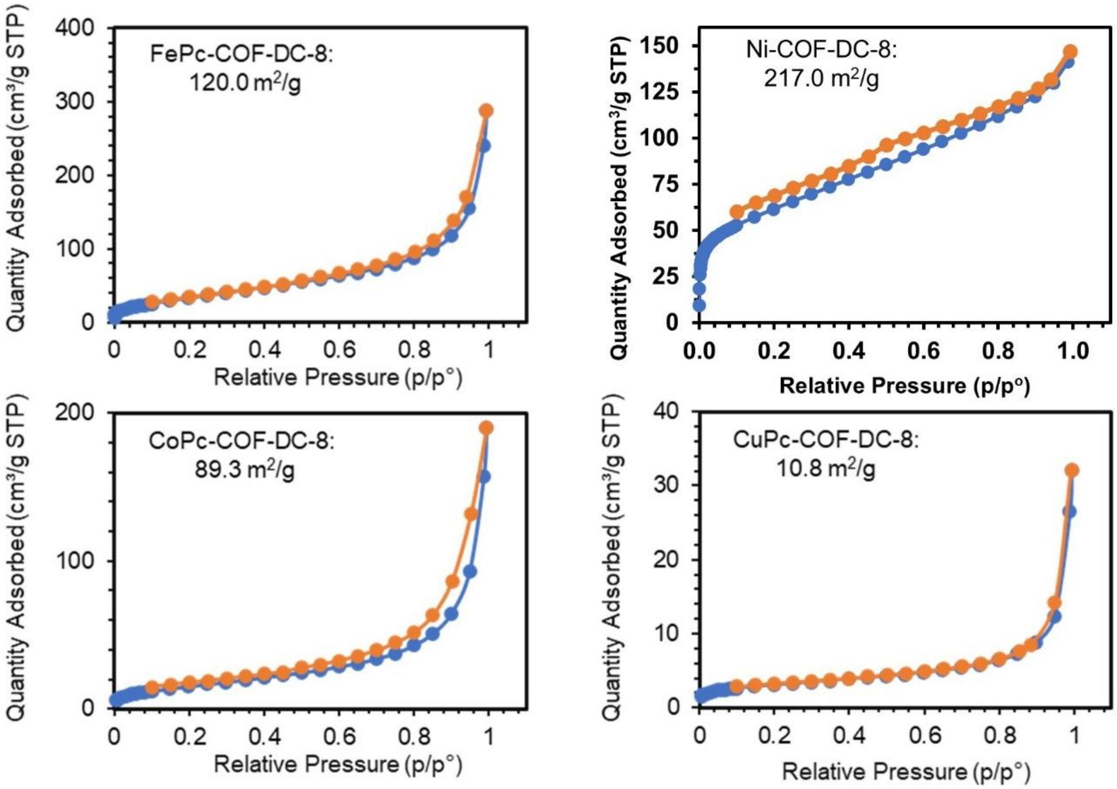

Figure S17. Adsorption isotherms for N_2 are shown for each M-COF-DC-8 as well as their respective specific surface areas (SSA), measured using the BET approach, (insets) expressed in units of m^2/g.

Our studies on specific surface area (SSA) analysis observed lower values for BET surface area for the COF materials under two important metrics: (1) The theoretical values for M-COF-DC-8 ~830 m^2/gram (estimated from the solvent-accessible surface area with a solvent radius of

2.1 Å, assuming fully accessible (001) crystallographic plane), and (2) SSA values previously reported for structurally similar COFs (CoPc-PDQ: 754 m^2/g, Ni- COF-DC-8: 420 m^2/g). We attributed the low SSA of the four materials we report to one of two possible issues. *First*, our activation procedures could be insufficient for proving accessible pores of the COFs by not removing impurities (e.g., oligomeric, unreacted organic components, or adsorbed solvent). The *second* possible issue is related to the degree of crystallinity of the COFs. A low degree of crystallinity could lead to low SSA by limiting the formation of pores or preventing access to pores within a framework due to stacking errors. These problems could be addressed by refining the synthetic procedures to obtain more crystalline compounds. The highest reported surface area for this class of materials, CoPc-PDQ:754 m^2/g, was synthesized from a 50:50 mixture of DMAc: ethylene glycol with acetic acid as a reaction promoter.[5, 6] Adopting the previously reported use of a mixed-solvent system, and the use of a milder promotor (AcOH compared to H_2SO_4 and pTSA), could help slow the reaction, solubilize reaction intermediates and oligomers, and help yield more crystalline materials.

The Cu-COF-DC-8 exhibited a micropore area of 2 m^2/g indicating a low micropore region. While minimal, it is possibly accessible to analytes used in the sensing studies.

XI. Conductivity of M-COF-DC-8 Materials

We assessed the bulk conductivity of the M-COF-DC-8 (M=Fe, Co) materials using a linear 4-point probe on a pressed pellet. The system we used consisted of a voltage source (typically outputting 23 V) and two voltmeters. The second voltmeter (usually a Milliampmeter in high-quality instruments) was placed used with a 1 kΩ resistor to allow calculation of the current. The tips spacing was 1.2 mm and the tip material was tungsten carbide. The pellets we tested were formed by compressing 20–50 mg of COF powder to 500 psi (as limited by our pellet press).

We assessed the bulk conductivity of the M-COF-DC-8 (M=Ni, Cu) materials using a Lucas Signatrone Pro4 equipped with a Keithley 2635 source meter. A pellet of ~23 mg of COF powder was compressed for 10 mins under a pressure of ~1000 psi using a 6 mm inner-diameter split sleeve pressing die. The pellet was used to calculate the bulk conductivity using **Equation S1** in which I (A) is the current, V is the voltage across the probes, s (cm) is the distance between the probes (1.25 mm), F (unitless) is the correction factor accounting for the diameter and thickness of the pellet. The calculated electrical conductivity was averaged from at least three measurements taken from different locations of the same pellet.

$$\sigma = \frac{I}{V}\frac{1}{2\pi sF} \qquad \textbf{Eq. S1}$$

Two different instruments were used for conductivity measurements as the access to the 4-point probe apparatus became available after the data collection for the M-COF-DC-8 (M=Fe, and Co) materials was completed.

XII. Chemiresistive Gas Sensing

To evaluate the COFs in chemiresistive applications each M-COF-DC-8 was sonicated into a suspension in H_2O. Devices were fabricated by drop casting 10 μL of COF suspension on 5 μm interdigitated microelectrodes (Au on glass) followed by air drying. While the suspensions were not homogeneous, similar device resistance ranges among the same COF devices indicated consistent material coverage. Electrodes were sourced from Metrohm AG.

The devices were then connected to a 44-position card connector (also referred to as an edge connector) and the headspace surrounding the devices was sealed in a custom-made Teflon chamber with inlet and outlet gas ports to create an encloser with gas inlet and outlet ports. To determine sensing parameters such as reversibility, rate of response, the magnitude of response, and limit of detection, we performed saturation experiments for each analyte and material combination. Each sensing experiment consisted of three steps. First, the resistance of the devices was brought to a constant value under a (0.5 L/min) flow of nitrogen. Second, the devices were exposed to a flow of a known analyte concentration for 30 min. Third, flow to the chamber was returned to N_2 to allow the devices to recover. At least three ($n \geq 3$) devices fabricated using material from the same COF synthetic batch were used to obtain statistically significant data for each sensing experiment. The reported sensing performance metrics were calculated by (i) taking the average of the devices in each individual sensing experiment and (2) reporting the standard deviation of the value.

Flow rates of dry N_2 and air were established with SmartTrack high flow mass flow controllers and the concentration of the analyte in the 0.5 L/min N_2 flow was adjusted by changing the flow rate of the 1% analyte gas using the Microtrack low flow mass flow controller. For sensing in humid environments, a humidified stream (5000 ppm H_2O) of either N_2 or air was generated using the Kintek Flexstream Base Model. Flow rates of humidified N_2 and air were established using the vapor generator (flow rate: 0.438 L/min, oven temperature: 80°C) after calibration of the stream to generate the correct 5000 ppm concentration. The concentration of the analyte in the 0.438 L/min humidified flow was adjusted by changing the flow rate of the 1% analyte gas using the Microtrack low flow mass flow controller. Sensing traces are shown in **Figures S19−S22**. Sensing experiments were performed at a two-electrode potential of 1000 mV in a series

multiplexer with each device being sampled every 0.5 seconds. Sensing experiments in humid environments were performed using one synthetic batch of COF.

The conductance change of the devices measured as current changes at the supplied voltage of 1000 mV, was normalized using **Equation S2** the reported response values as a percentage (-$\Delta G/G_0$):

$$\frac{-\Delta G}{G_0} = \frac{-(A_f - A_i)}{A_i} \times 100\% \qquad \textbf{Eq. S2}$$

Where A_f is the final current and A_i is the initial baseline current in units of Ampere. This method of normalization minimizes the impact of some device fabrication features in the final sensing characterization.

Theoretical LOD Calculations

Theoretical LOD values reported in the main text **Table 1** were calculated following reported methods.[7] The noise-based deviation in the $-\Delta G/G_0$ was calculated as the root mean squared (RMS) value of the baseline trace before exposure to analyte. 600–1200 data points (N) on the baseline were selected and fitted using a 5th order polynomial. The sum of the squared residuals (V_{x^2}) was calculated from **Equation S3**, where Y_i is the measured $-\Delta G/G_0$ of the baseline and Y is the value calculated from the polynomial fit. The root mean squared deviation (rms_{noise}) was calculated using **Equation S4**. To find value m (slope), we plotted the response ($-\Delta G/G_0$) after 30 minutes analyte exposure vs the concentration of analyte and used linear regression to generate an equation of best fit. The calculation of the slope of the linear regime of the $-\Delta G/G_0$ versus concentration plots contained at least three data points as precedented in literature.[5] With both rms_{noise} and m, the theoretical LOD was calculated using **Equation S5**.

$$V_{x^2} = \Sigma(Y_i - Y)^2 \qquad \textbf{Eq. S3}$$

$$rms_{noise} = \sqrt{(V_{x^2}/N)} \qquad \textbf{Eq. S4}$$

$$LOD = 3 * (rms_{noise}/m) \qquad \textbf{Eq. S5}$$

Based on literature precedent for calculating the estimated theoretical LOD, the order of magnitude different between experimental gas concentration range and theoretical LOD can range from 2−5.[5, 8, 9] In our work this range is 2−3 orders of magnitude in difference between the experimental and theoretical LOD depending on the specific gas. Based on the assumption of a linear relationship of signal versus concentration extending into the ppb range, we rely on established calculations to extrapolate theoretical LODs. Future investigations of the experimental sensing limit would provide meaningful understanding of the material−analyte interactions.

Concentration Dependence

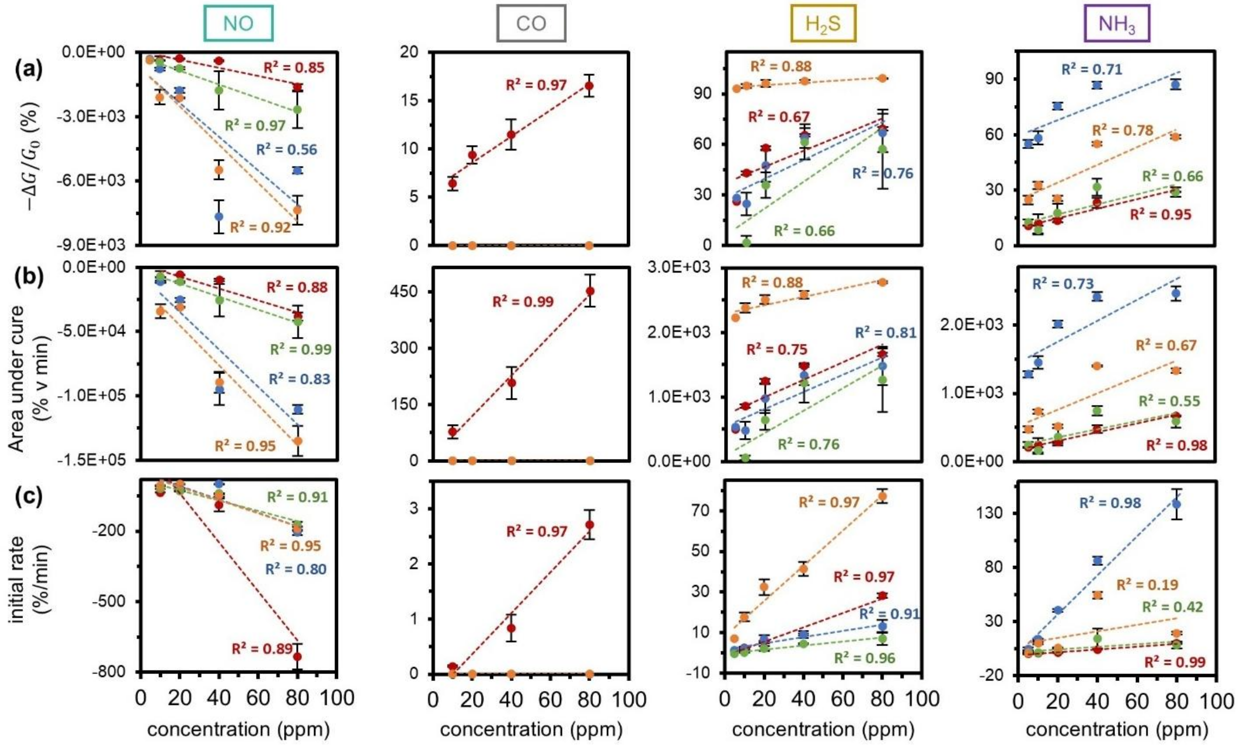

Figure S18. Linear relationships between performance metrics and analyte concentrations. (**a**) The response at saturation during 30 minute exposure, (**b**) area under the curve, and (**c**) initial rate of response of M-COF-DC-8 (M=Fe, Co, Ni, Cu) to gaseous analytes within the first 1–2 min of exposure plotted to show linear relationships. The values at a range of concentrations were then plotted to determine the initial rate of response to the gases (left–right) NO, CO, H_2S, and NH_3. Note: M-COF-DC-8 (M=Fe, Co, Ni, Cu) is represented by red, blue, green, and orange traces, respectively.

Challenging Sensor Array with Potential Interferants

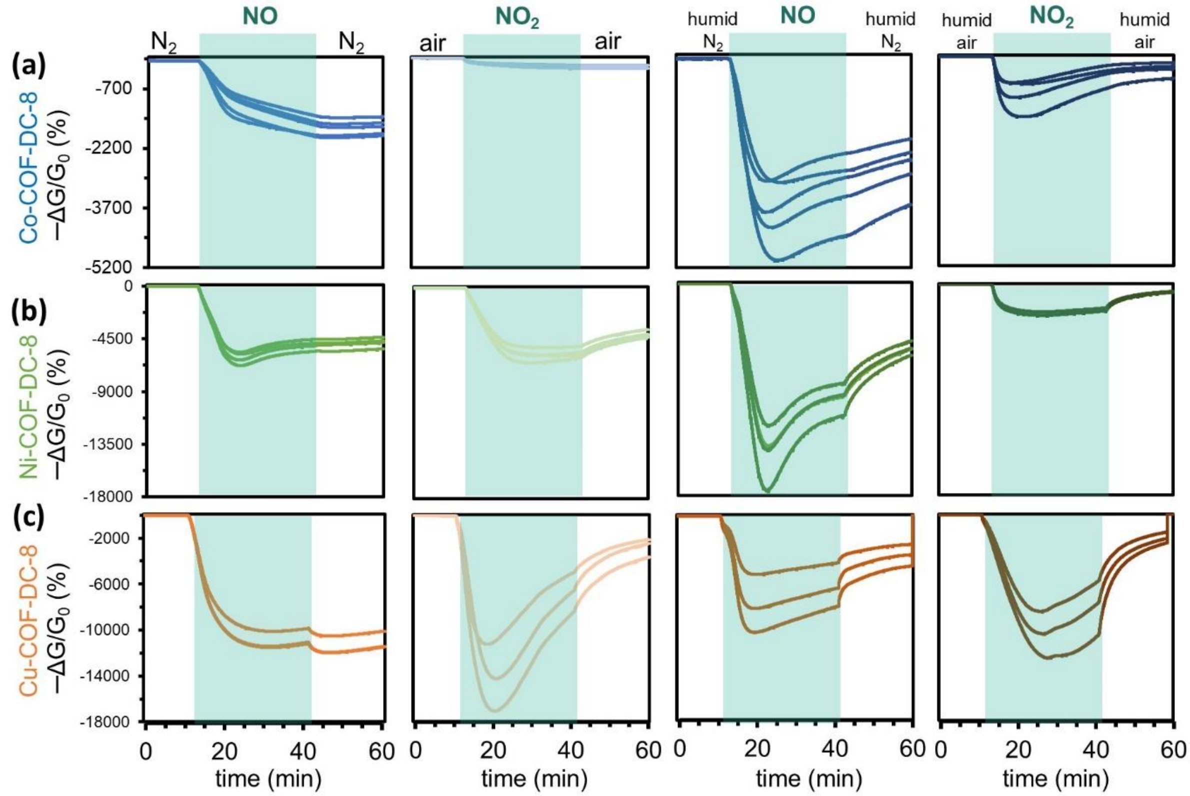

Figure S19. Chemiresistive measurements for the M-COF-DC-8 (**a–c**: M=Co, Ni, Cu) materials exposed to concentrations of 80 ppm of NO in various environments of (left to right) dry N_2, dry air, humidified N_2, and humidified air. Exposures of the devices to H_2S (shaded blue regions) was maintained for 30 minutes before the flow was returned to 100% background environments. Measurements were made using an applied potential of 1.0 V and a measurement interval of 0.5 s. (Note: NO_2 was used in air environments since NO will oxidize to NO_2.

As seen in **Figure S19**, both COFs' chemiresistive response shows a change in direction of response before the end of the 30-minute NO exposure in humid N_2 environments. This response could be due to a concentration dependent response. The decrease in conductance after the first couple minutes of exposure could be due to a secondary analyte generated by a reaction between NO_x and a competing analyte (oxygen or water)[10, 11] or a secondary mode of NO interaction with the humidified COF. This result requires further literature search and future testing before proposing a possible mechanistic explanation.

A similar phenomenon of change in direction of response is seen when sensing NH_3 using Co-COF-DC-8. It is possible that a secondary interaction due to the humidity results in another

preliminary host-guest interaction than what is observed in dry environments. However, after time, the dominant interaction results in a decrease in conductance, which we hypothesize is the reducing NH_3 gas removing charge carriers from the COF. This change in the direction of response before the end of the 30 minute exposure to NH_3 as seen in **Figure S22** could be attributed to a concentration dependence.[12] Due to the presence of water vapor, during the introduction of NH_3, a secondary interaction either due to NH_3 interacting with the COF material in a different manner. This deviation in chemiresistive response could also arise due to a secondary species generated by a reaction between NH_3 and a competing analyte. For instance, in atmospheric science, NH_3 has shown to oxidize to NO_x or other nitrogenous compounds when in the presence of OH radicals.[13, 14] It is possible that a oxidizing byproduct could result in the deviation in initial ROR, however further literature searches are required to validate what interactions/reactions are possible in the sensing headspace/on the surface of the COF device to generate competing analytes capable of resulting in chemiresistive deviations.

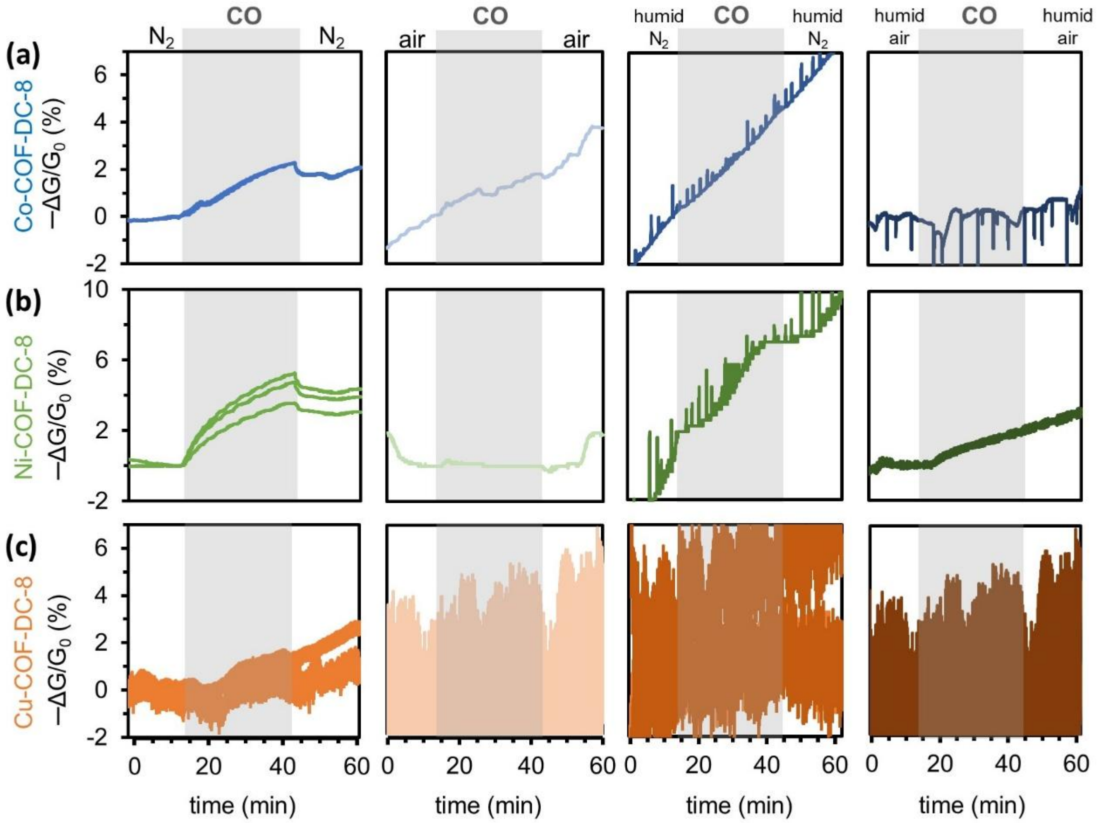

Figure S20. Chemiresistive measurements for the M-COF-DC-8 (**a**–**c**: M=Co, Ni, Cu) materials exposed to concentrations of 80 ppm of CO in various environments of (left to right) dry N_2, dry air, humidified N_2, and humidified air. Exposures of the devices to CO (shaded grey regions) was maintained for 30 minutes before the flow was returned to 100% background environments. Measurements were made using an applied potential of 1.0 V and a measurement interval of 0.5 s.

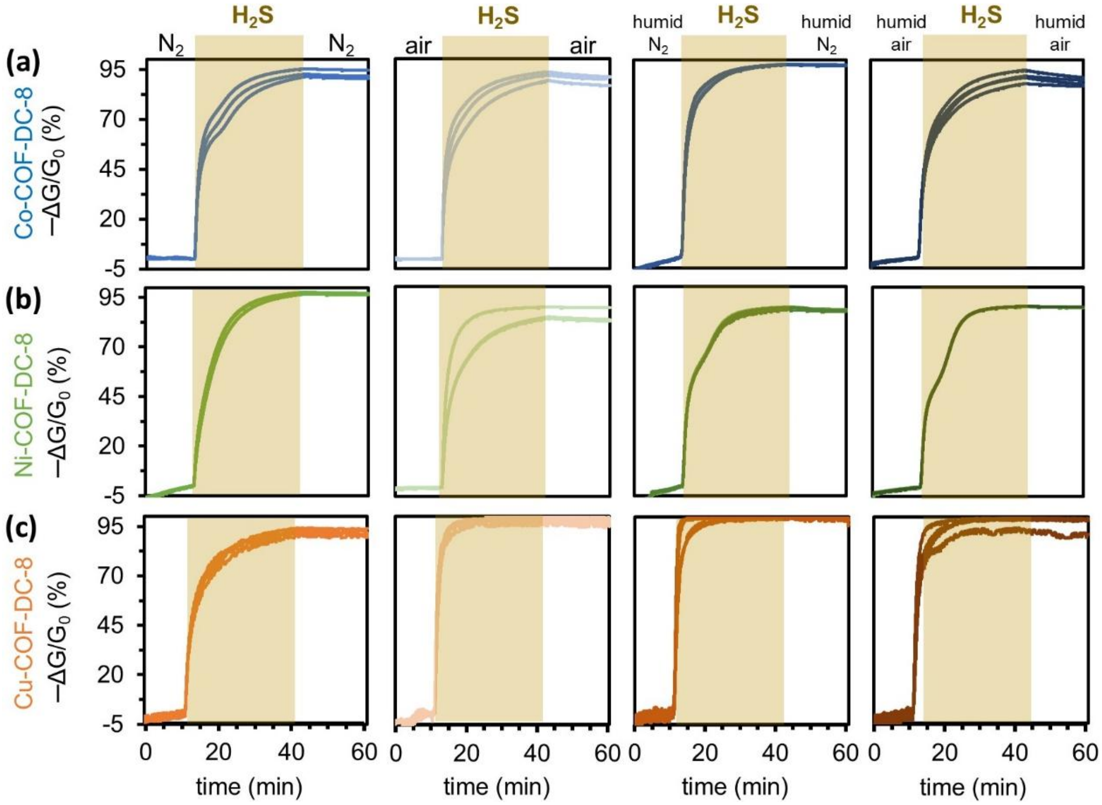

Figure S21. Chemiresistive measurements for the M-COF-DC-8 (**a**–**c**: M=Co, Ni, Cu) materials exposed to concentrations of 80 ppm of H_2S in various environments of (left to right) dry N_2, dry air, humidified N_2, and humidified air. Exposures of the devices to H_2S (shaded yellow regions) was maintained for 30 minutes before the flow was returned to 100% background environments. Measurements were made using an applied potential of 1.0 V and a measurement interval of 0.5 s.

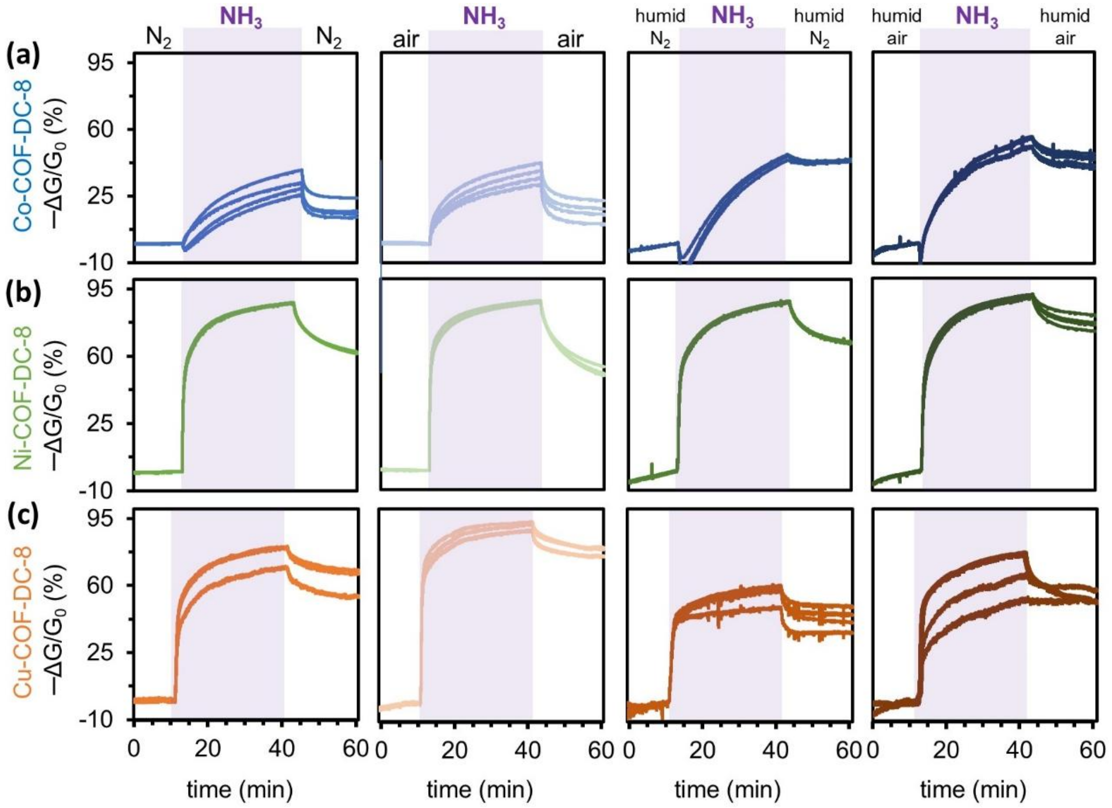

Figure S22. Chemiresistive measurements for the M-COF-DC-8 (**a**–**c**: M=Co, Ni, Cu) materials exposed to concentrations of 80 ppm of NH_3 in various environments of (left to right) dry N_2, dry air, humidified N_2, and humidified air. Exposures of the devices to NH_3 (shaded purple regions) was maintained for 30 minutes before the flow was returned to 100% background environments. Measurements were made using an applied potential of 1.0 V and a measurement interval of 0.5 s.

Despite initial success using Fe-COF-DC-8 devices to sense CO, it was difficult to replicate the sensing results due to the inability to reproducibly synthesize highly crystalline, conductive Fe-COF-DC-8. The sensing trace of **Figure S23a** was generated using the synthetic condition described in trial 2 of **Table S2** and exhibited a response of 16.5% ± 1.1 upon exposure to 80 ppm CO. However, material of similar quality could not be reproduced using the previous method. As such, trial 3 of **Table S2** was used to synthesize Fe-COF-DC-8 with lower crystallinity. This material was used to generate the sensing traces in **Figure S23b**. While the response of the lower crystalline Fe-COF-DC-8 was half in magnitude compared to the crystalline material, this result highlights the ability of material containing FePc subunits to outperform the other MPc (M=Co, Ni, and Cu) containing materials. Coupled with the spectroscopic evidence seen in DRIFTs, FePc centered materials merit future study to investigate CO−material interaction and CO detection.

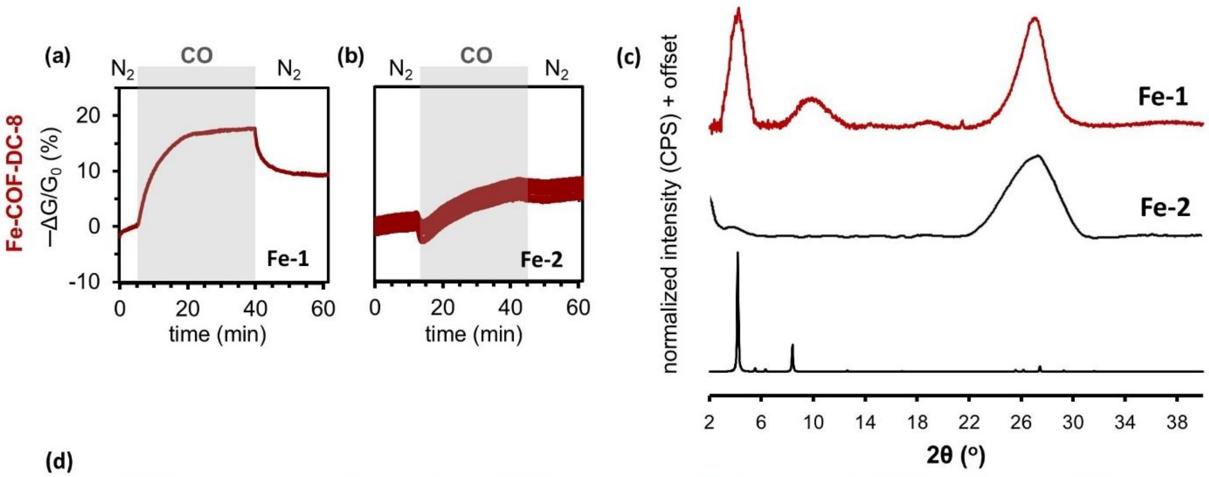

(d)

	Precursor Identity	[Prec.] mM	Prec. mass (mg)	Solvent	Additive	Additive : Prec.	Rxn Time (days)	Temp (°C)
Fe-1	$FePc(NH_2)_8$-HCl	3.9	32.4	NMP	PTSA	21.2	3	150
Fe-2	$FePc(NH_2)_8$	14.5	20.0	NMP	PTSA	20	3	185

Figure S23. Sensing traces of Fe-COF-DC-8 exposed to 80 ppm CO using different batches of devices synthesized from COF samples from synthetic (**a**) trial 2 and (**b**) trial 3 from **Table S2**. (**c**) pXRD of different Fe-COF-DC-8 samples used for sensing CO. (**d**) Table describing the synthetic conditions used to form the two Fe-COF-DC-8 samples described above.

	(a)	(b)
COF batch (trial from **Table S2**)	2	3
Applied Voltage (V)	1.0	3.0
$-\Delta G/G_0$ (%)	16.5 ± 1.1	7.0 ± 1.1
Initial RoR (%/min)	2.7 ± 0.3	−1.7 ± 0.4
Device Fabrication	Unknown	3 rounds of dropcasting & dry in 85°C oven<br>3 rounds of dropcasting & dry overnight
Device Resistance (MΩ)	Unknown	10.8−16.0

Table S12. Details on experiments used to generate sensing traces (**a−b**) in **Figure S23.**

The ability to detect CO was dependent upon effective material deposition on the IDE as evidenced by **Figure S24** in which the same COF synthetic batch was used in three different drop casting procedures. These results demonstrates that device fabrication method highly impacts the device resistance and ability to reliably detect CO.

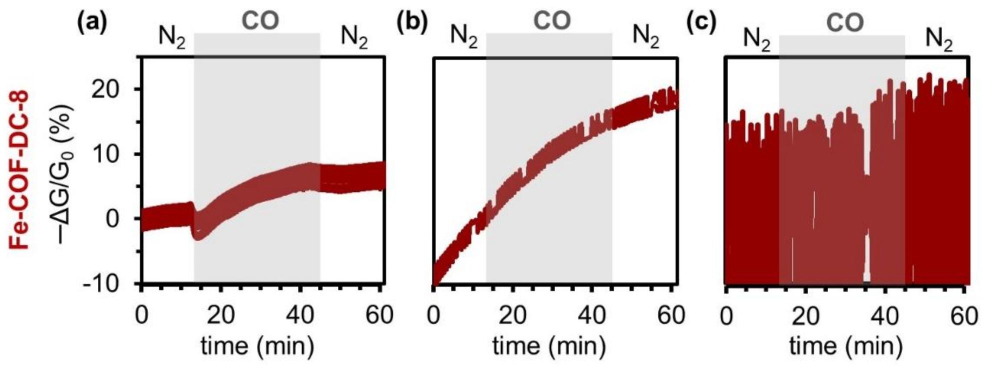

Figure S24. Sensing traces of Fe-COF-DC-8 exposed to 80 ppm CO using different batches of devices synthesized the same COF synthetic batch using different device fabrication methods (**a−c**).

	(a)	(b)	(c)
COF batch (trial from **Table S2**)	3	3	3
Applied Voltage (V)	3.0	3.0	3.0
$-\Delta G/G_0$ (%)	7.0 ± 1.1	No response	No response
Initial RoR (%/min)	−1.7 ± 0.4	No response	No response
Device Fabrication	3 rounds of dropcasting & dry in 85°C oven<br>3 rounds of dropcasting & dry overnight	1 round of dropcasting, dry overnight	1 round of dropcasting, dry in 85°C oven
Device Resistance (MΩ)	10.8−16.0	35.0−55.0	10.0−20.0

Table S13. Details on experiments used to generate sensing traces (**a−c**) in **Figure S24.**

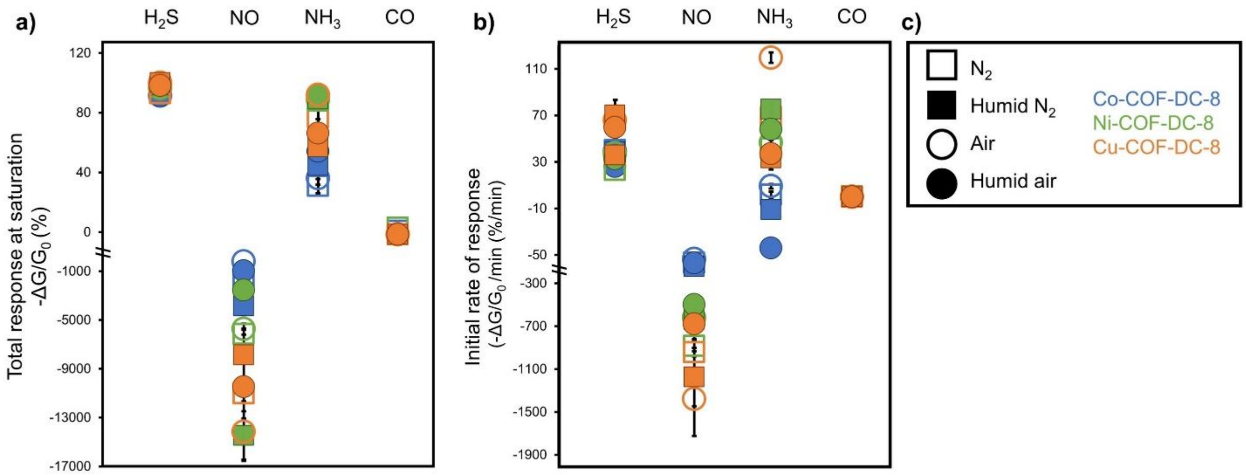

Figure S25. a) Responses at saturation, and **b)** initial RoR of M-COF-DC-8 (M=Co. Ni, Cu) devices for sensing 80 ppm H_2S, NO, NH_3 , and CO in dry N_2, dry air, humid N_2 (18% RH), and humid air (18% RH) environments. **c)** legend for graphs **a–b**. Note: the positive and negative y-axes are different scales to appropriately incorporate the range of data.

Batch-to-batch Reproducibility

To evaluate batch-to-batch reproducibility, sensing data were compared between devices fabricated using COF materials synthesized from two distinct batches. **Figure S26** provides side-by-side comparisons of the response of COF devices exposed to 80 ppm analyte (NO, CO, H_2S, or NH_3) in dry N_2 environments. Data from batch 1 corresponds to the main text **Figure 3**, while data from batch 2 are presented in **Figures S19−S22**.

Despite minor deviation observed in the maximum response and the initial rate of response, all devices of the same COF material exhibited responses within the same order of magnitude and consistent directionality. These batch-to-batch variations highlight opportunities for further refinement and optimization, particularly in COF synthesis (e.g. crystallinity and morphology), device preparation (e.g. suspension concentration and sonication time), and the aging of COF materials and suspensions.

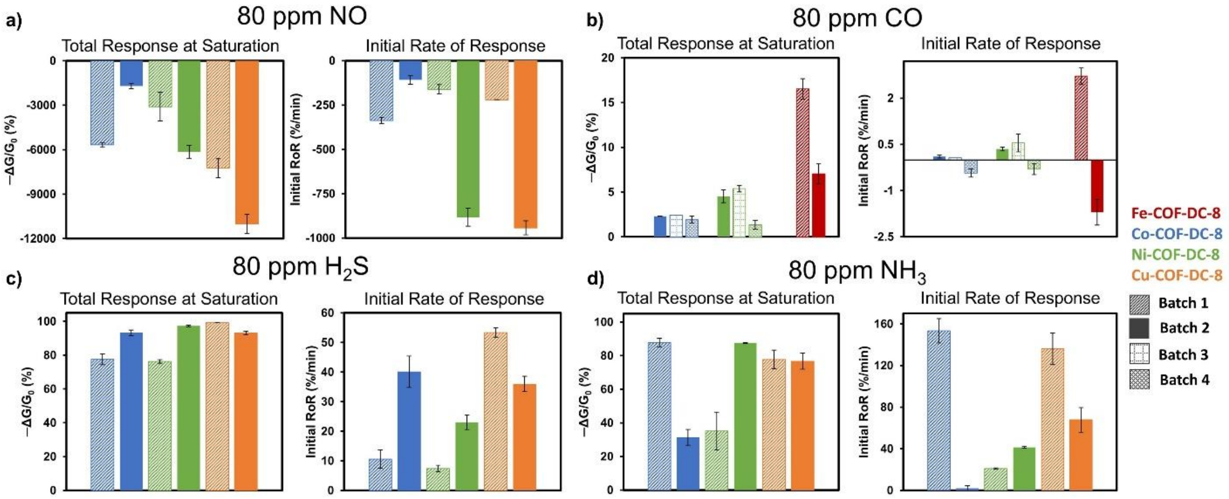

Figure S26. Batch-to-batch reproducibility testing of two different COF device batches used to sense 80 ppm of the following analytes in dry N_2: (a) NO, (b) CO, (c) H_2S, and (d) NH_3. Note: Fe-COF-DC-8 devices were not used for NO, H_2S, and NH_3. Note: where there appears to be no bar present, that is due to a value of 0.

XIII. Spectroscopic Characterization of M-COF-DC-8 with Probe Gases by DRIFTS, XPS, and EPR

Future studies could aim to use time-dependent density functional theory (TDDFT) to iteratively probe the correlation between the presence of redox-active defect sites and the spectral changes induced by gas exposure.

DRIFTS of M-COF-DC-8 powders exposed to NO

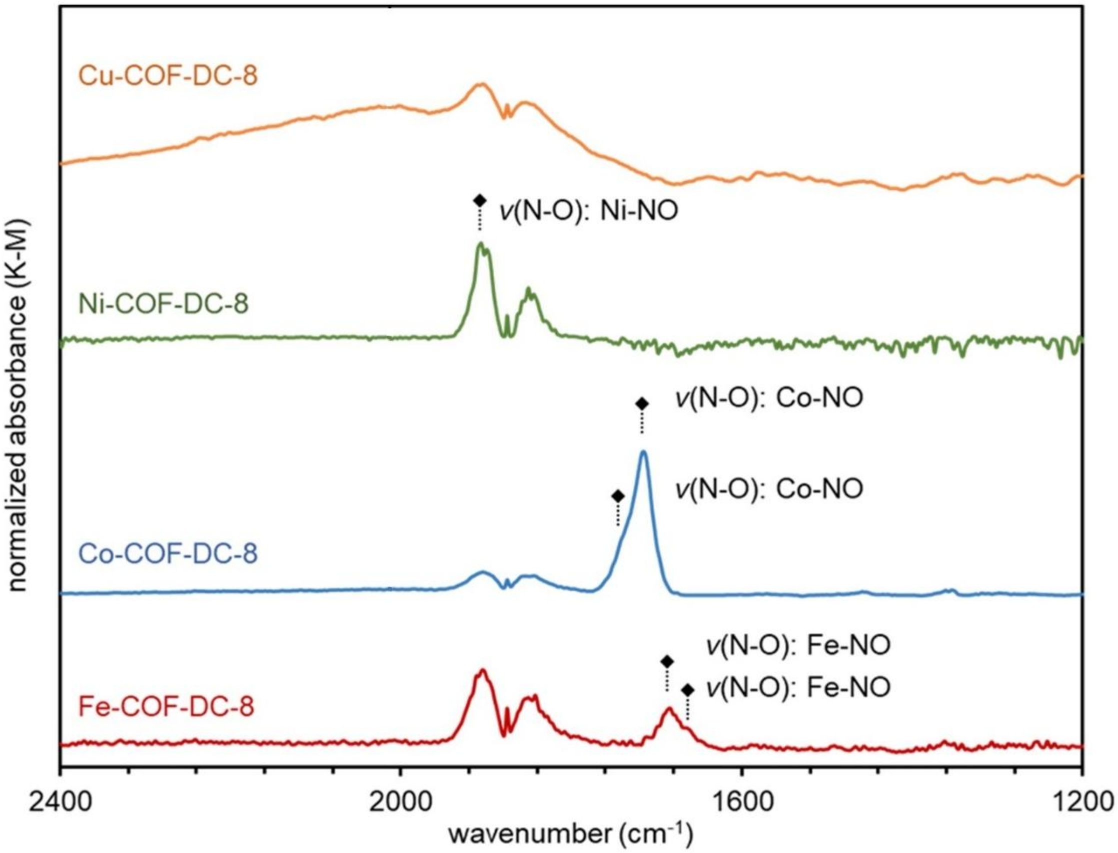

Fe-COF-DC-8	Co-COF-DC-8	Ni-COF-DC-8	Cu-COF-DC-8	Assignment
1694	1715	1900	x	ν(NO): M-NO

Figure S27. DRIFTS difference spectra of M-COF-DC-8 samples after 10 min of exposure to NO gas (1 % in N_2).

DRIFTS of M-COF-DC-8 powders exposed to CO

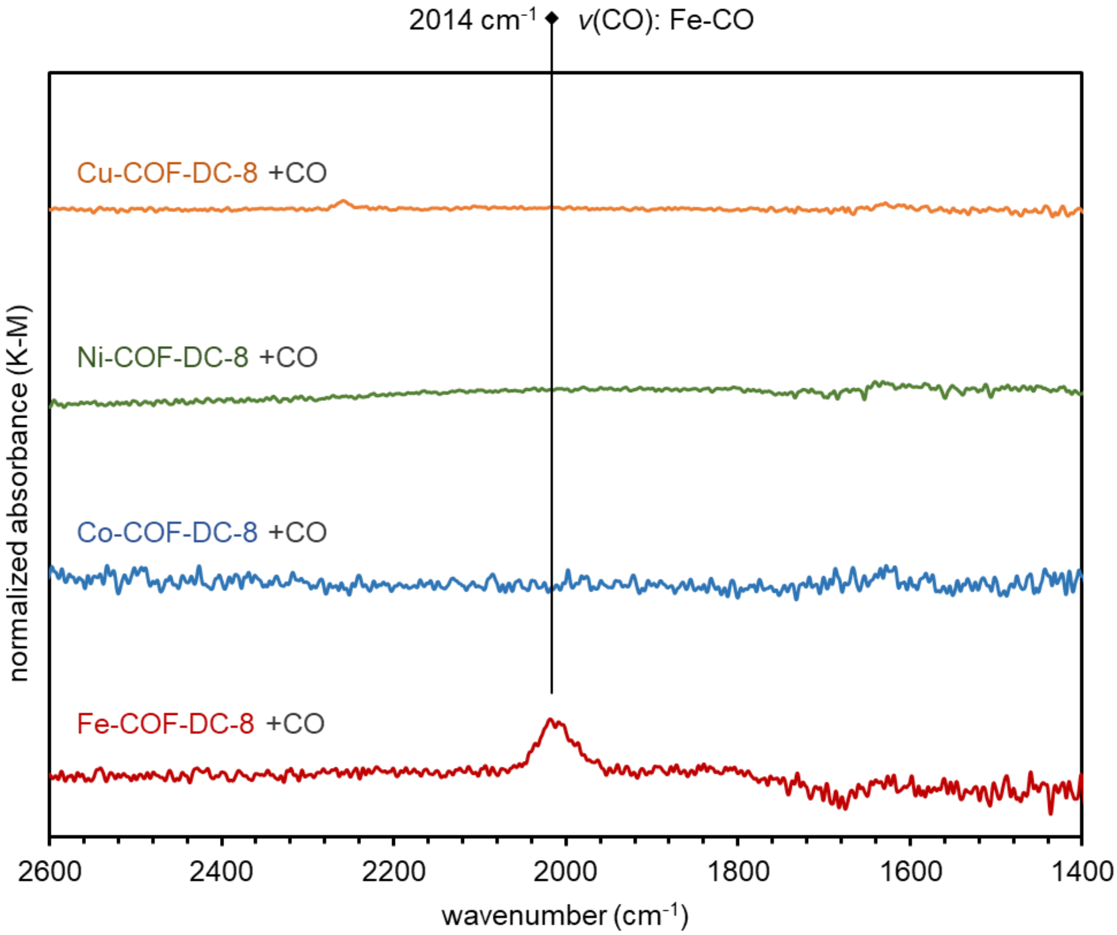

Figure S28. DRIFTS difference spectra were plotted as the difference before and after exposure of M-COF-DC-8 samples to CO gas (1 % in N_2). Exposure time for the included spectra was 20 min followed by 4 min purge with N_2.

DRIFTS of M-COF-DC-8 powders exposed to H_2S

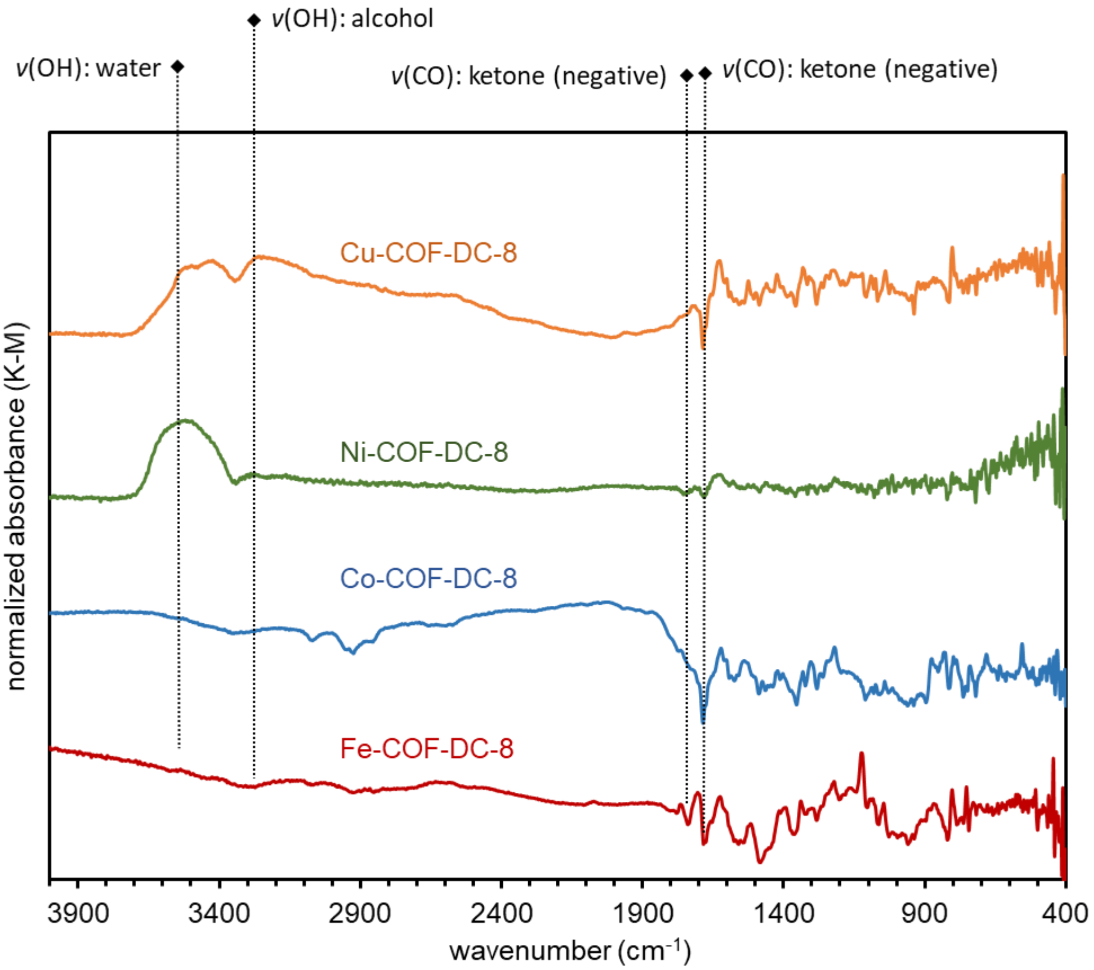

Fe-COF-DC-8	Co-COF-DC-8	Ni-COF-DC-8	Cu-COF-DC-8	Assignment
3501 (weak)	x	3561	3511	v(OH): H_2O
	3293	3292	3292	v(OH): NH
1742<br>1675	1756<br>1685	1743<br>1680	1737<br>1685	v(C=O) ketone
broad weak bands (2981–1972 cm^{-1})				aromatic C-H<br>v(N-H)

Figure S29. DRIFTS spectra were plotted as the difference before and after exposure of M-COF-DC-8 samples to H_2S gas (1 % in N_2). Exposure time for the displayed spectra was 20 min under NH_3 before flushing with N_2 for 4 min.

DRIFTS of M-COF-DC-8 powders exposed to NH_3

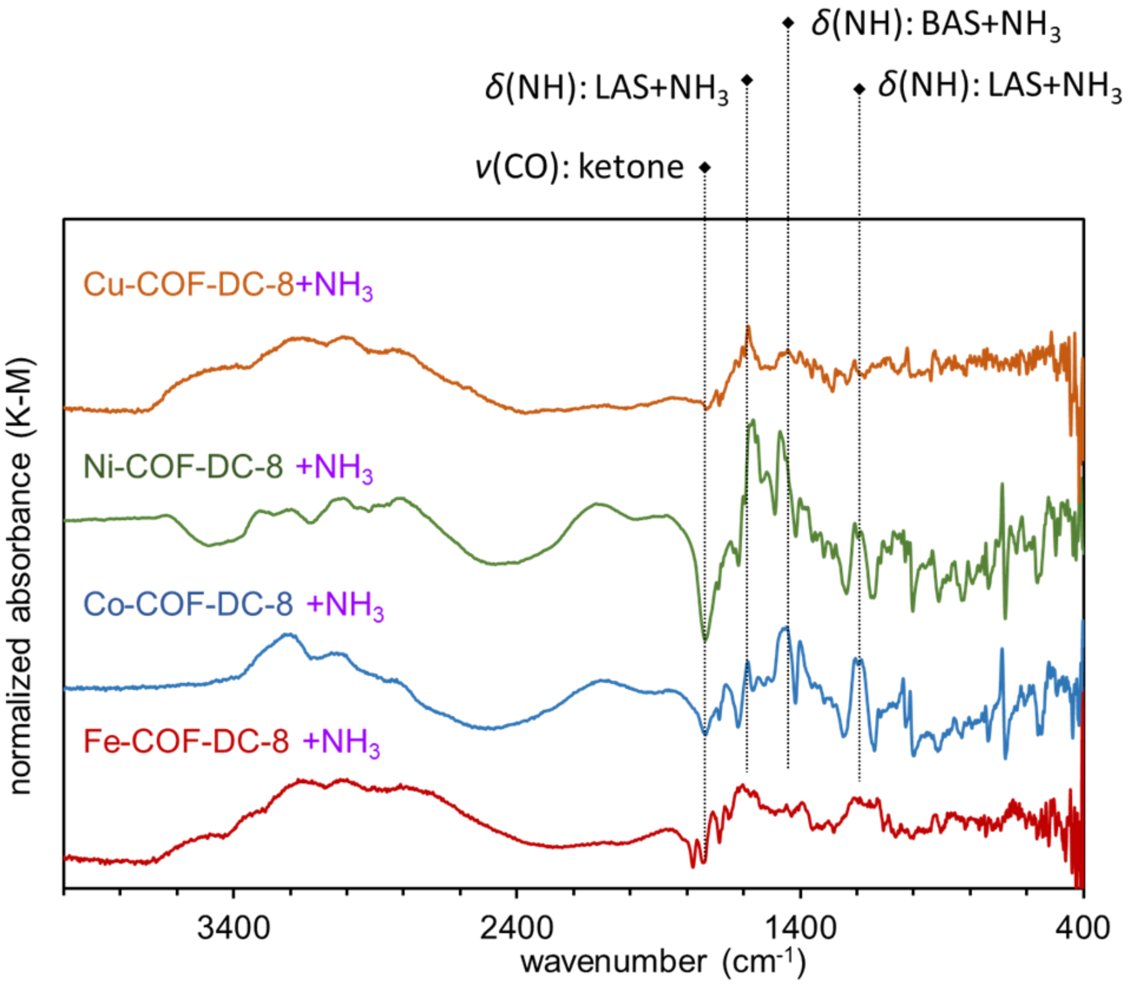

Figure S30. DRIFTS spectra were plotted as the difference before and after exposure of M-COF-DC-8 samples to NH_3 gas (1 % in N_2). Specific interactions for each material are labeled as either Lewis acid (LAS) type interactions or Brønsted acid (BAS) type interactions. These interactions are summarized below. The exposure time for the displayed spectra was 20 min under NH_3 before flushing with N_2 for 4 min.

Fe-COF-DC-8	Co-COF-DC-8	Ni-COF-DC-8	Cu-COF-DC-8	Assignment
3510<br>3336<br>3144<br>2995	x<br>x<br>3194<br>3029	*neg* 3496<br>3314<br>3182<br>2985	3519<br>x<br>3177<br>2974	*v*(OH): H_2O $_{ads}$<br>*v*(NH): NH_3 $_{ads}$<br>*v*(NH): NH_3 $_{ads}$<br>*v*(NH): NH_3 $_{ads}$
2814	2810	2810	2820	*v*(C-H): pyr/Pc
2091<br>1846	2097<br>1846	2098<br>1856	x<br>1846	overtones: Ar
neg 1738	*neg* 1736	*neg* 1735	*neg* 1719	v(CO): ketone
1580	*x*	1571	1578	$\delta(NH_3)$: LAS
x	1445	1442	1475	$\delta(NH_4^+)$: BAS
1192	1194	1193	x	$\delta(NH_3)$: LAS

Table S14. Summary of absorption bands and their assignments observed in the difference spectra (**Figure S30**) for each COF after exposure to NH_3.

DRIFTS of unsubstituted MPc analogs exposed to analytes

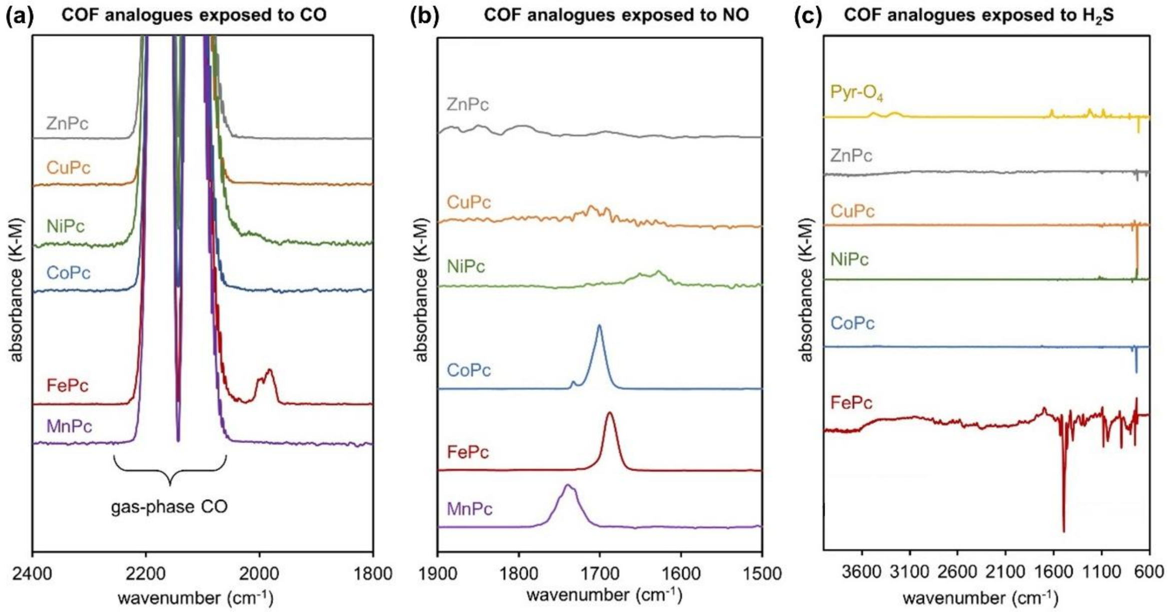

Figure S31. DRIFTS spectra of COF analogs (a) under 1% CO atmosphere (balance N_2), (b) after dosing 1% NO atmosphere (balance N_2) and purging with N_2, and (c) under 1% H_2S atmosphere (balance N_2). COF analogs used are unsubstituted MPc (M=Mn, Fe, Co, Ni, Cu, Zn) monomers and pyrene-tetraone.

XPS of M-COF-DC-8 After Exposure to Probe Gases

XPS after exposure to NO. While NO did not contain any unique elements that could aid in the interpretation of XPS analysis, the propensity for NO to be oxidized to NO_2 or NO_3 compounds having higher oxidation states of N allowed the identification of additional species adsorbed to the surface of the COFs by examining the N 1s region.

Fe-COF-DC-8 + NO

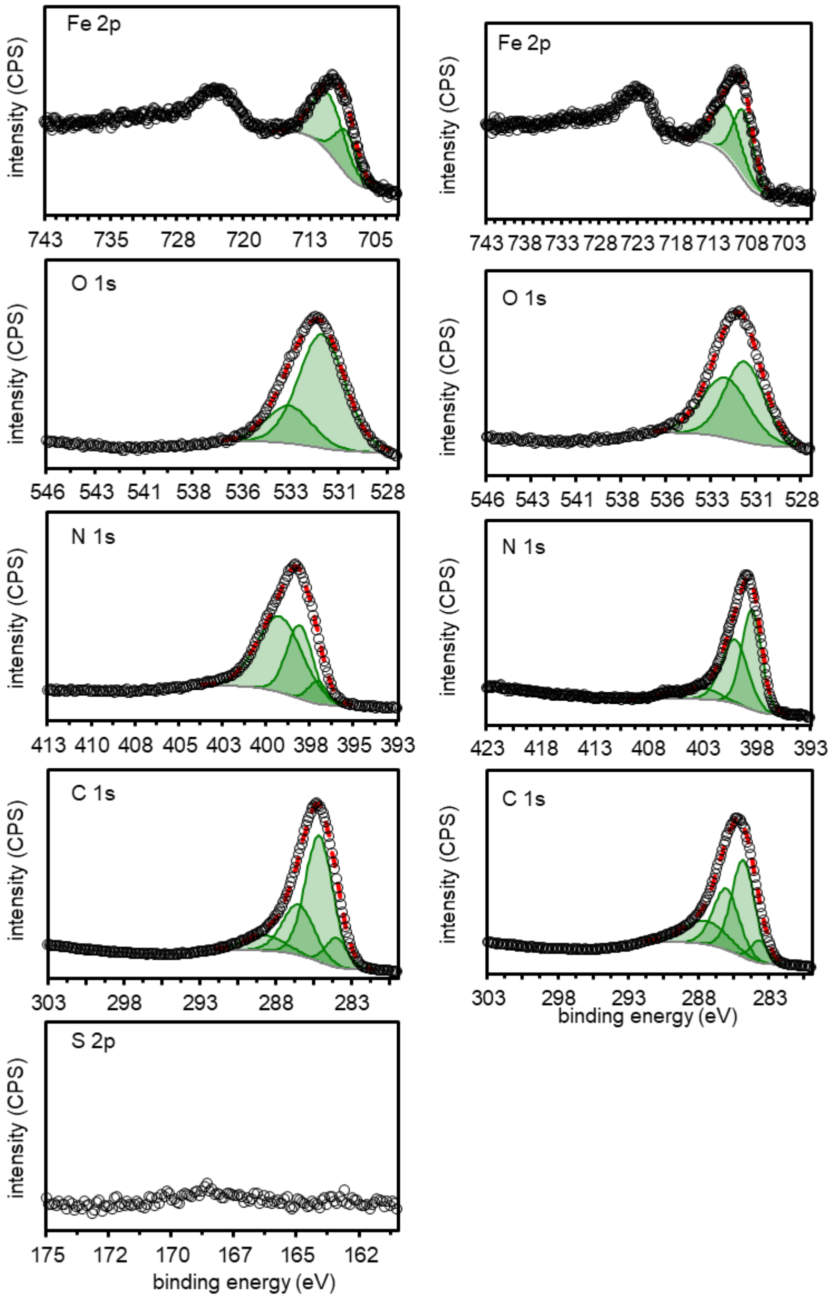

Figure S32. XPS analysis of the elemental regions of pristine FePc-COF-DC-8 (left) and material after exposure to NO (right). Two key changes were observed in the spectrum above compared to the pristine material. We observed a new component in the N 1s region at 405 eV which we assigned to NO_x^- species bound to the framework.

Co-COF-DC-8 + NO

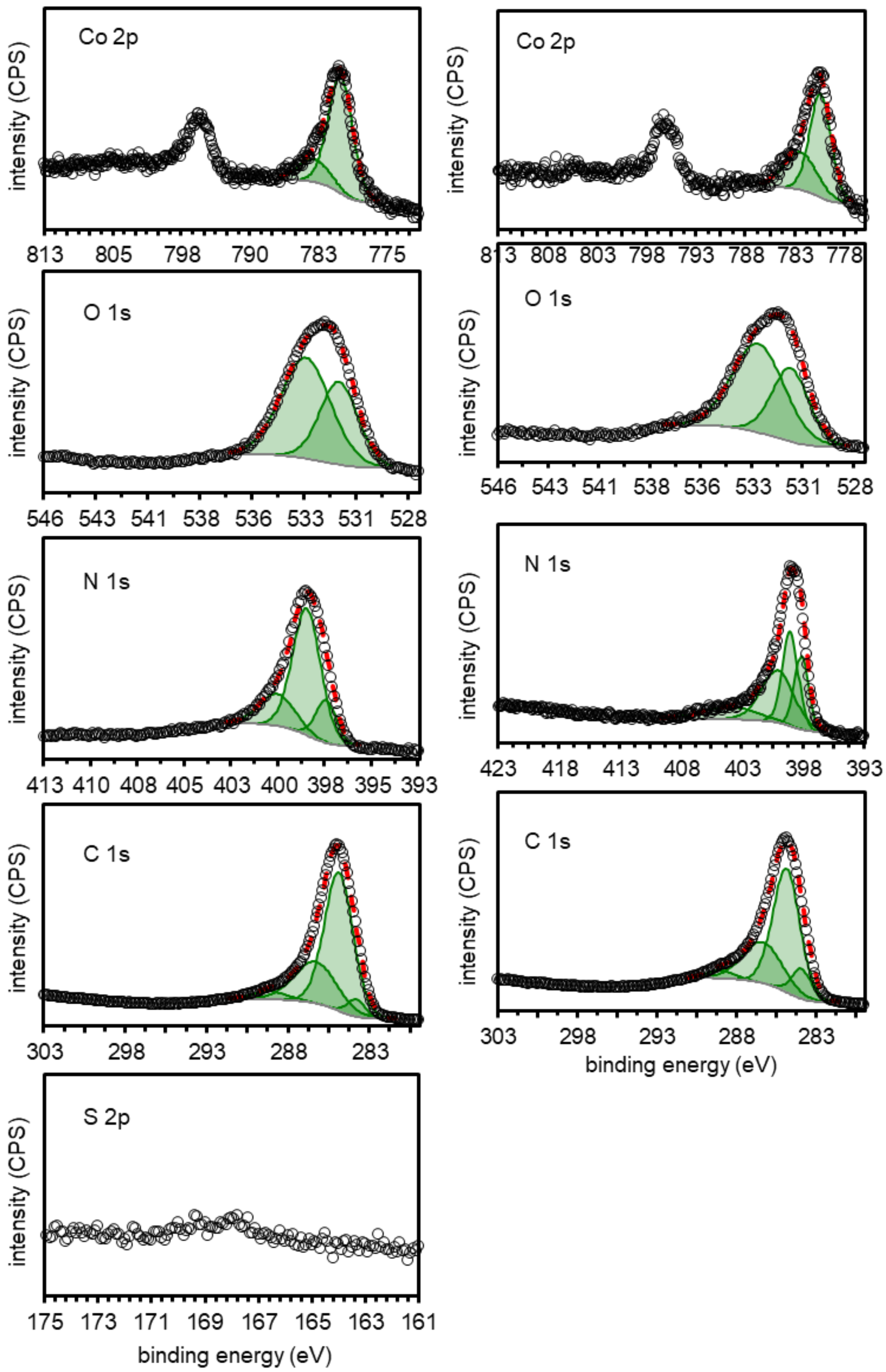

Figure S33. XPS analysis of the elemental regions of pristine CoPc-COF-DC-8 (left) and material after exposure to NO (right). We observed a new component in the N 1s region at 405 eV which we assigned to NO_x^- species bound to the framework. No changes to the metal oxidation state were observed.

Ni-COF-DC-8 + NO

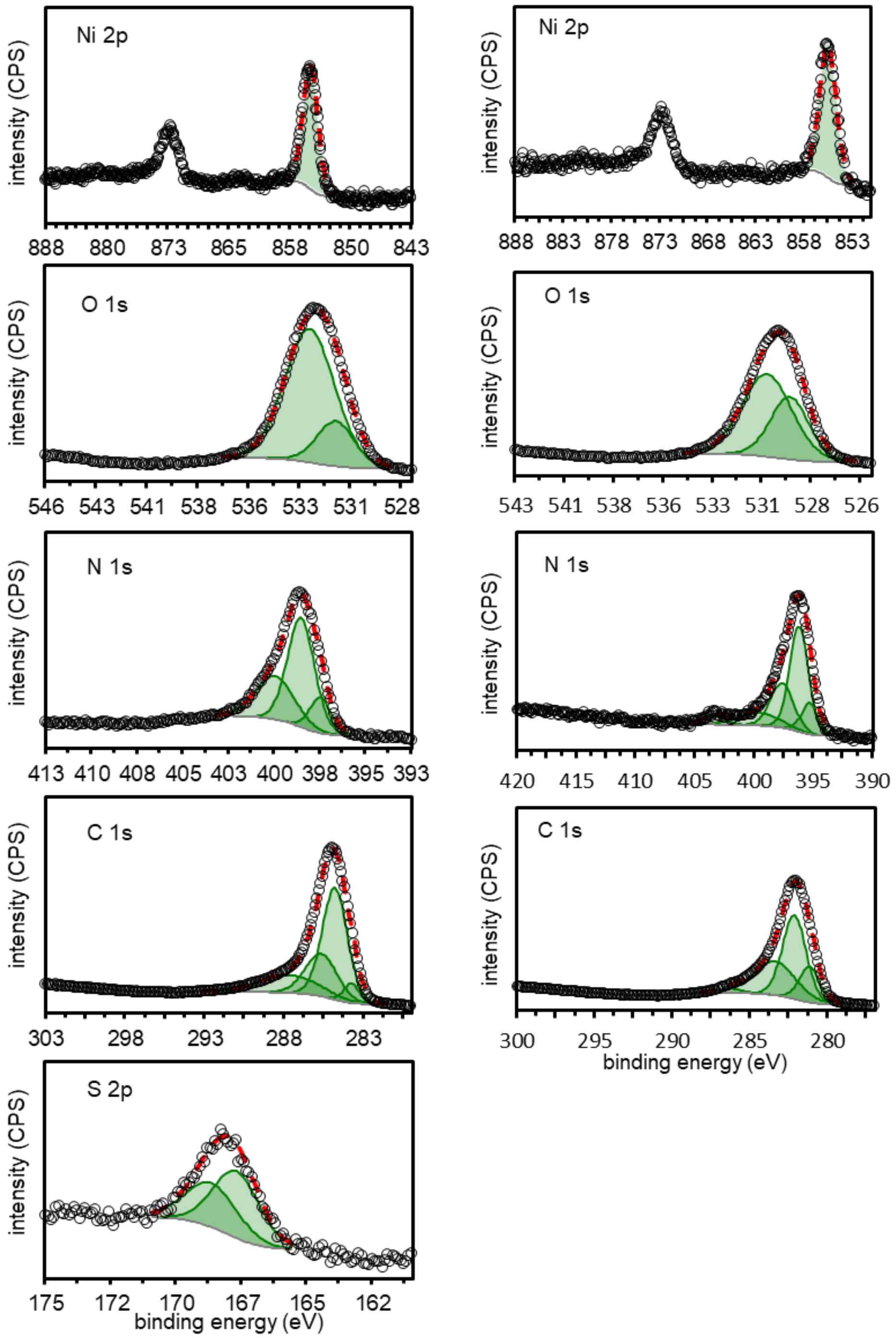

Figure S34. XPS analysis of the elemental regions of pristine NiPc-COF-DC-8 (left) and material after exposure to NO (right). We observed a new component in the N 1s region at 405 eV which we assigned to NO_x^- species bound to the framework. No changes to the metal oxidation state were observed.

Cu-COF-DC-8 + NO

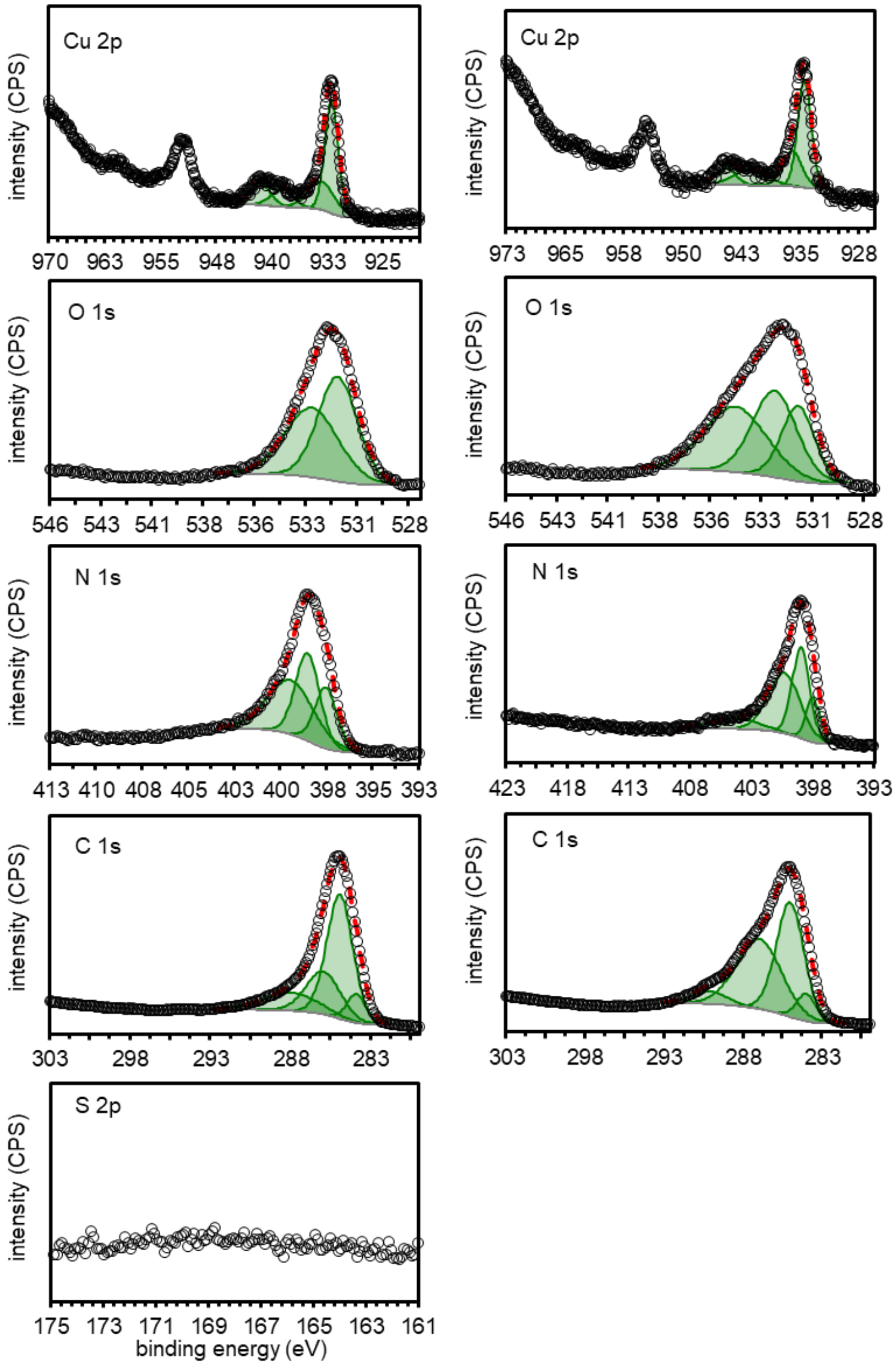

Figure S35. XPS analysis of the elemental regions of pristine CuPc-COF-DC-8 (left) and material after exposure to NO (right). We observed a new component in the N 1s region at 405 eV which we assigned to NO_x^- species bound to the framework. No changes to the metal oxidation state were observed. However, carbon-based portions of the framework appeared to be oxidized (component at 288 eV increased after exposure to NO).

XPS after exposure to CO. The elemental regions anticipated for CO did not present a fully orthogonal method of characterizing the adsorbed probe gas. Instead, comparisons were drawn between the emission spectra of the elemental regions of the pristine material and after exposure to CO.

Fe-COF-DC-8 +CO

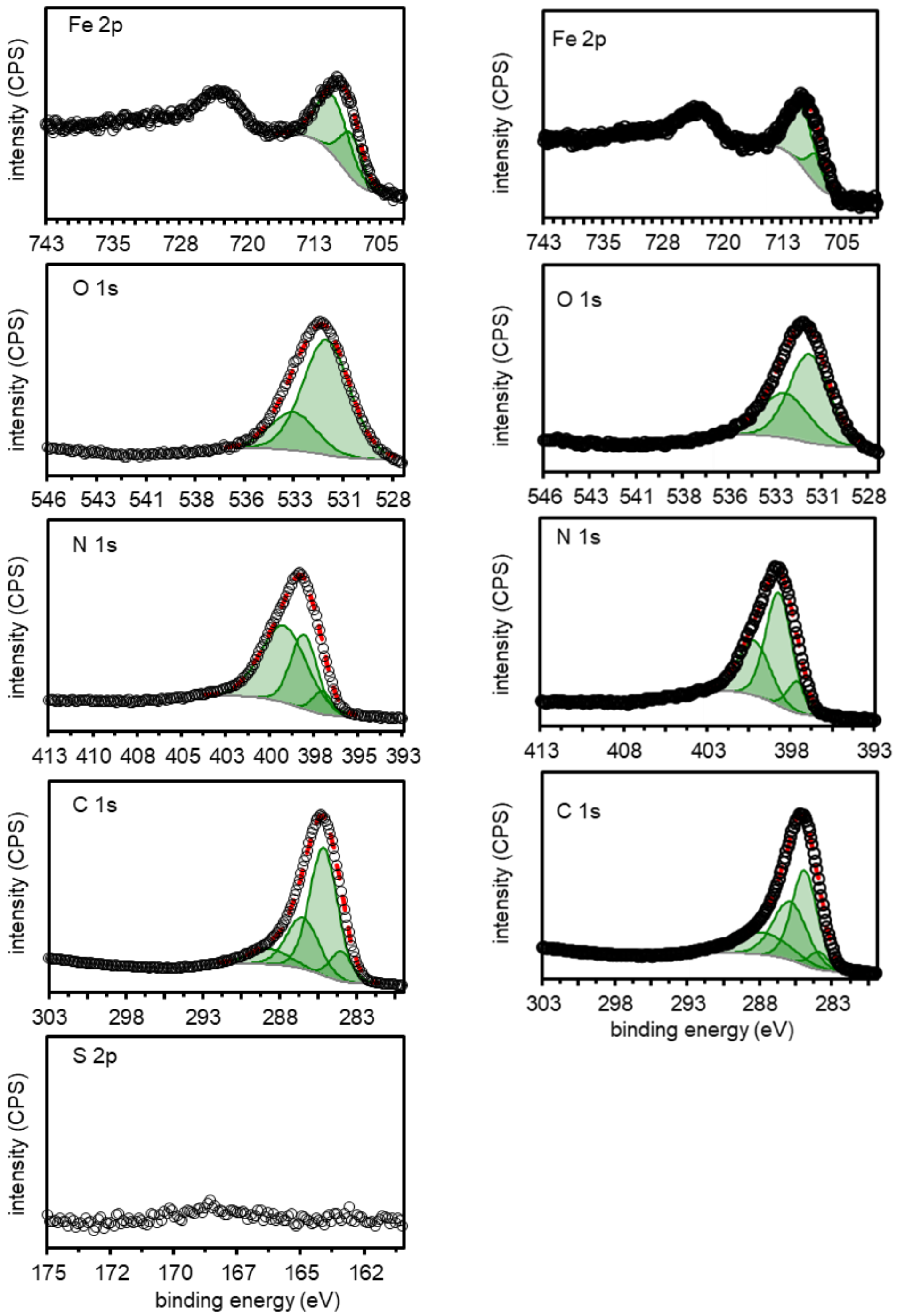

Figure S36. XPS analysis of the elemental regions of pristine FePc-COF-DC-8 (left) and material after exposure to CO (right). No key differences were observed in the spectra obtained from the pristine material and the one obtained for the material exposed to CO.

Co-COF-DC-8 +CO

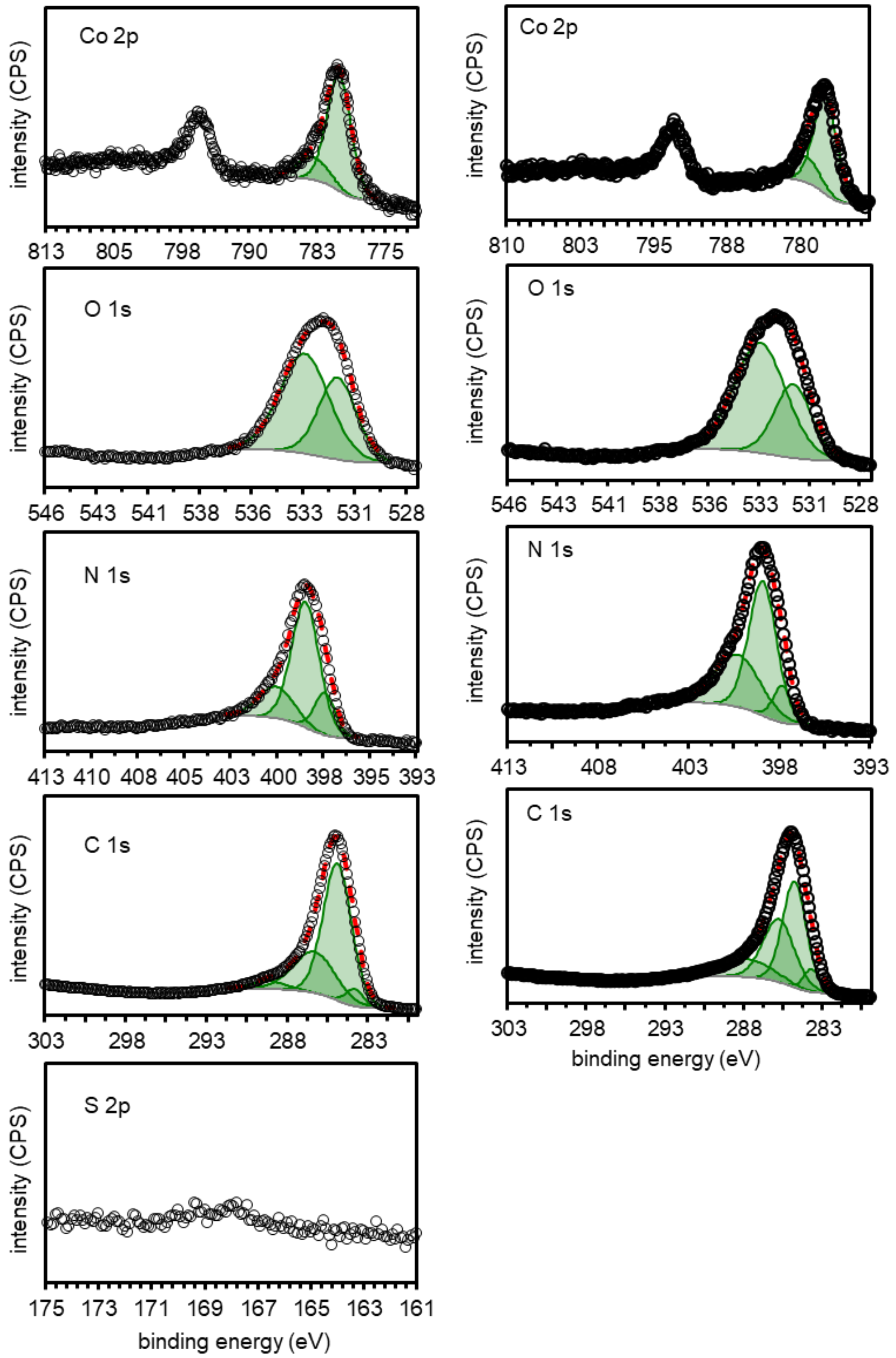

Figure S37. XPS analysis of the elemental regions of pristine CoPc-COF-DC-8 (left) and material after exposure to CO (right). No key differences were observed in the spectra obtained from the pristine material and the one obtained for the material exposed to CO.

Ni-COF-DC-8 + CO

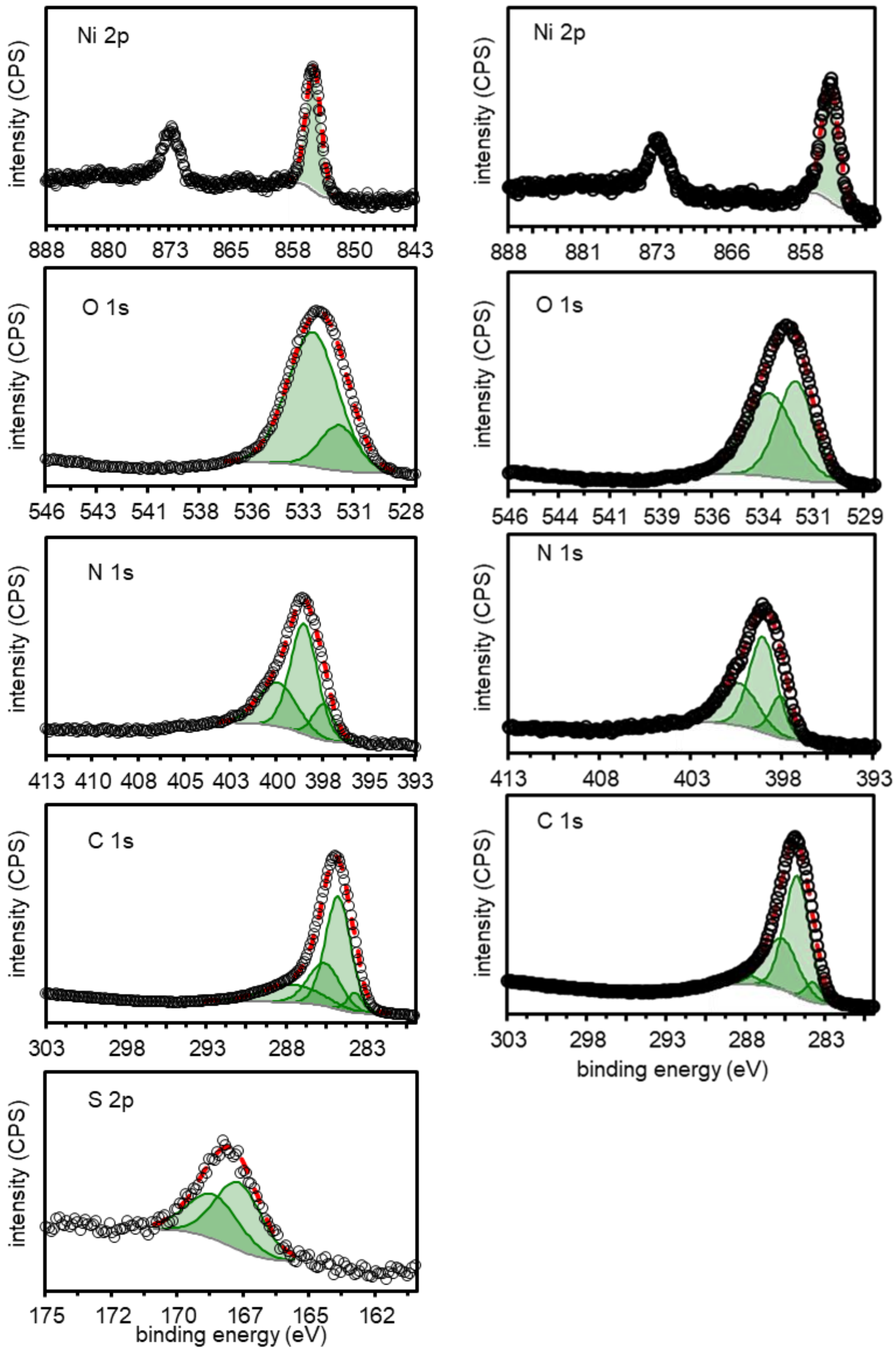

Figure S38. XPS analysis of the elemental regions of pristine NiPc-COF-DC-8 (left) and material after exposure to CO (right). No key differences were observed in the spectra obtained from the pristine material and the one obtained for the material exposed to CO.

Cu-COF-DC-8 + CO

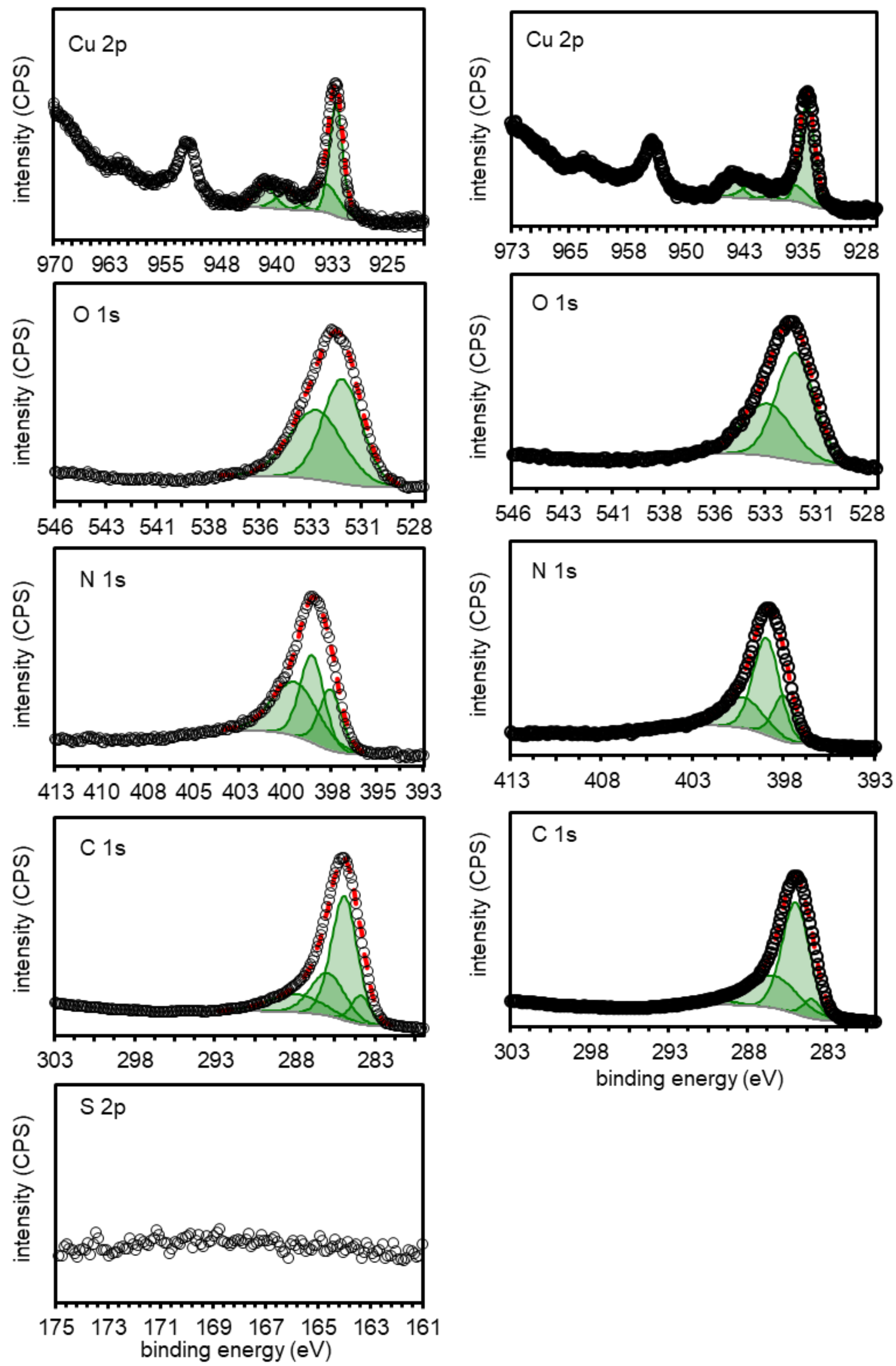

Figure S39. XPS analysis of the elemental regions of pristine CuPc-COF-DC-8 (left) and material after exposure to CO (right). No key differences were observed in the spectra obtained from the pristine material and the one obtained for the material exposed to CO.

XPS analysis after exposure to H_2S. The primary region of interest was the region containing the S $2p_{3/2}$ and S $2p_{1/2}$ emission lines of S-species.

Fe-COF-DC-8 +H_2S

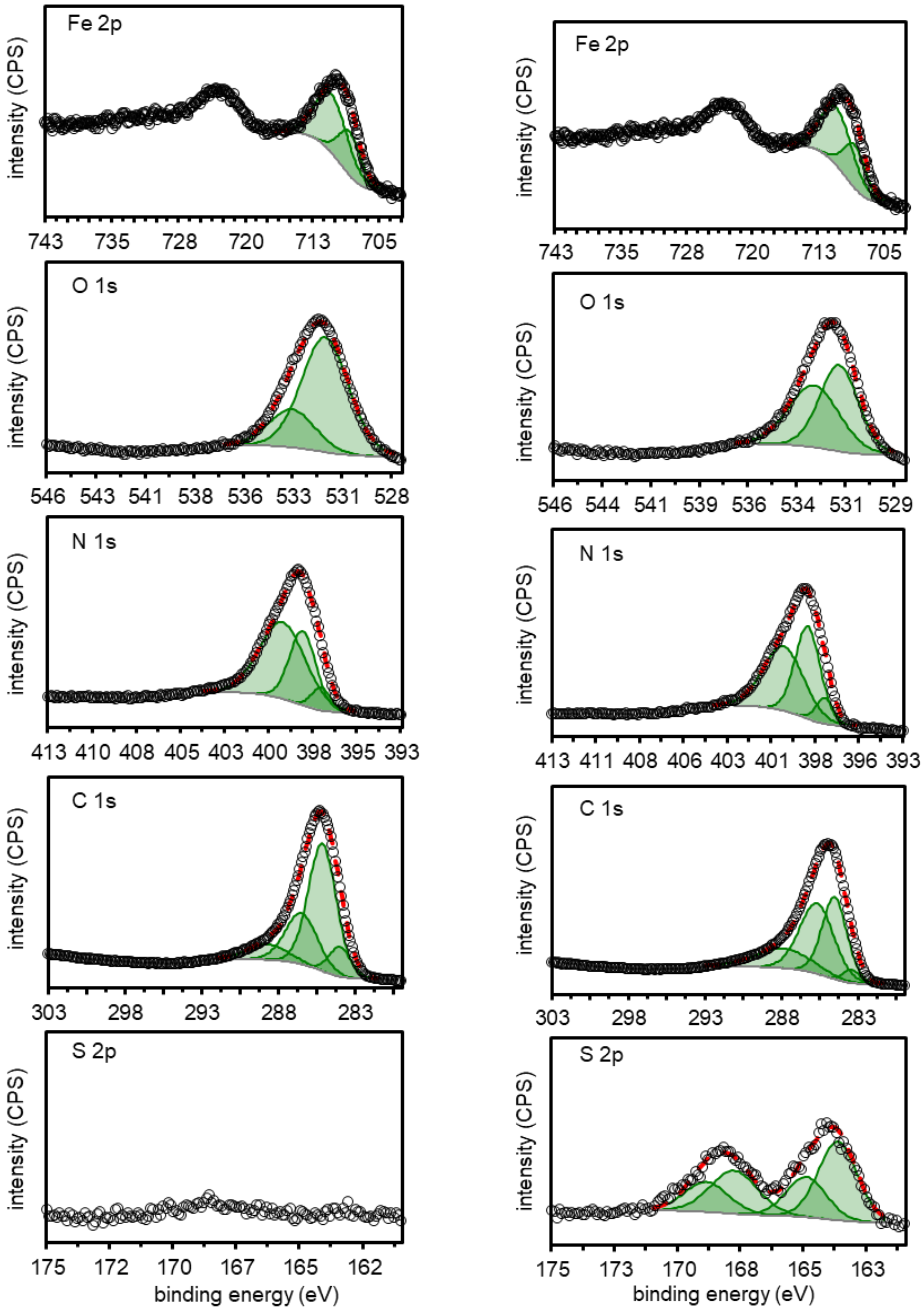

Figure S40. XPS analysis of the elemental regions of pristine FePc-COF-DC-8 (left) and material after exposure to H_2S (right). Two sets of emission lines were observed in the region 160–175 eV.

Co- COF-DC-8 +H_2S

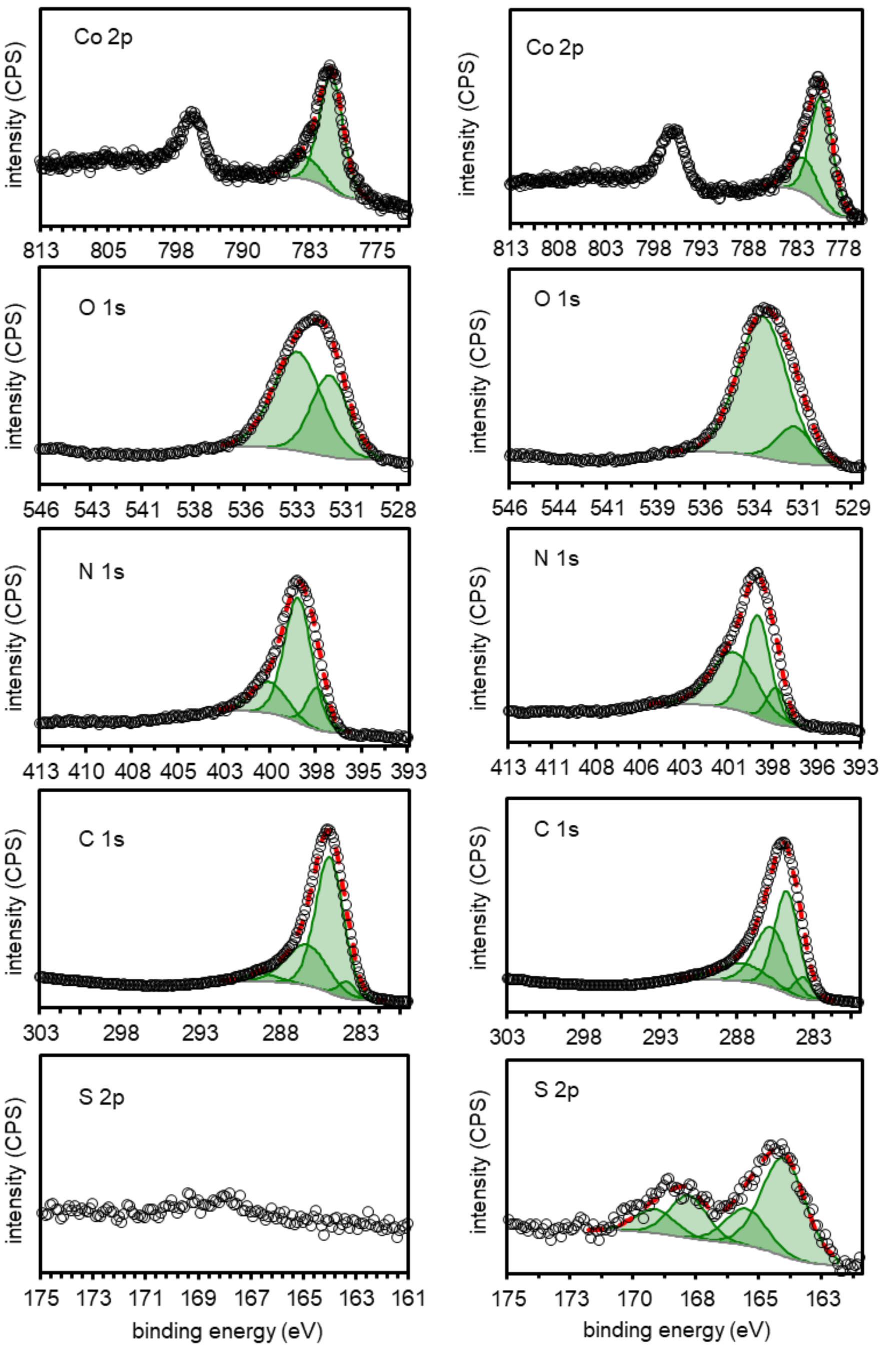

Figure S41. XPS analysis of the elemental regions of pristine CoPc-COF-DC-8 (left) and material after exposure to H_2S (right). Two sets of emission lines were observed in the region 160–175 eV.

Ni- COF-DC-8 +H_2S

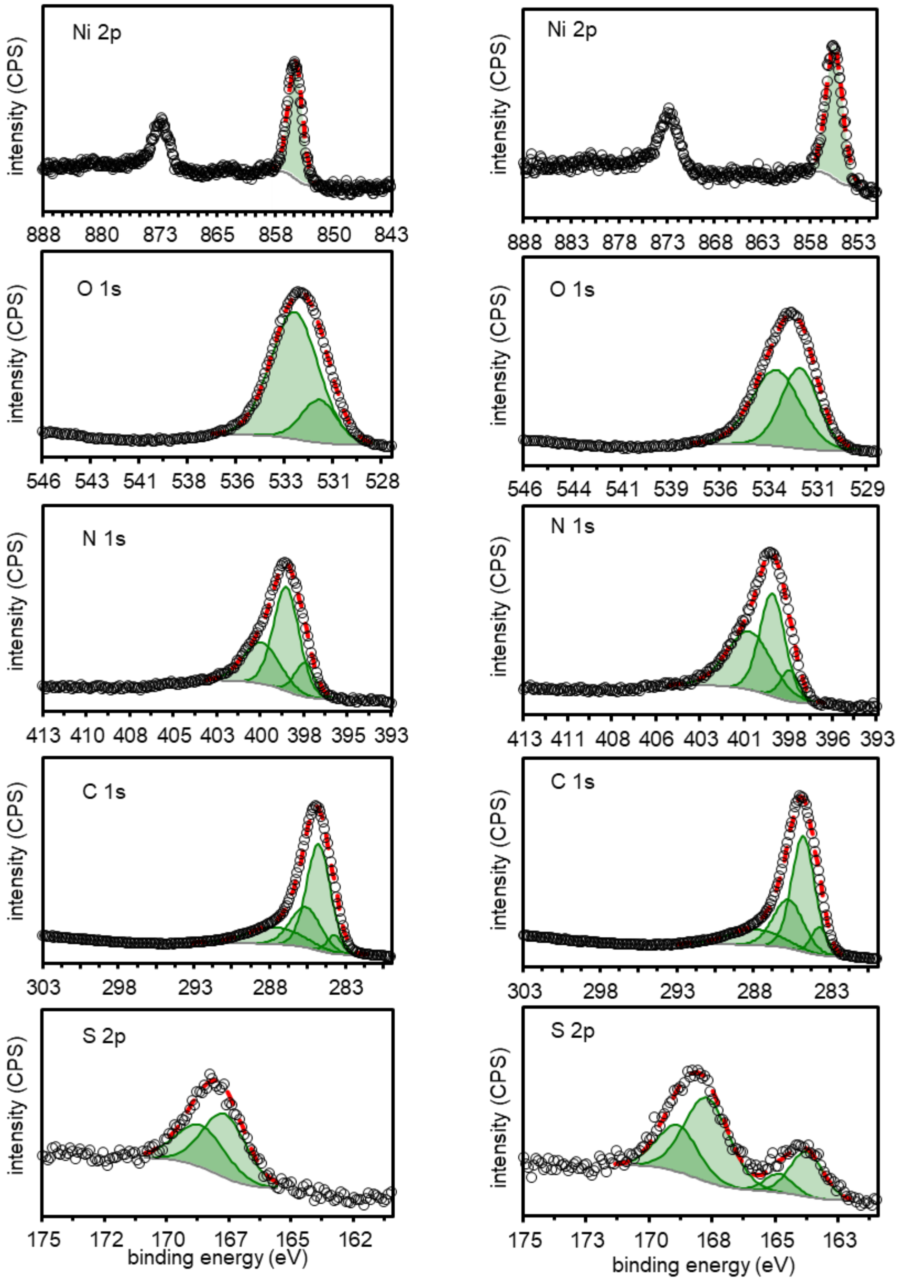

Figure S42. XPS analysis of the elemental regions of pristine NiPc-COF-DC-8 (left) and material after exposure to H_2S (right). Two sets of emission lines were observed in the region 160–175 eV.

Cu-COF-DC-8 +H_2S

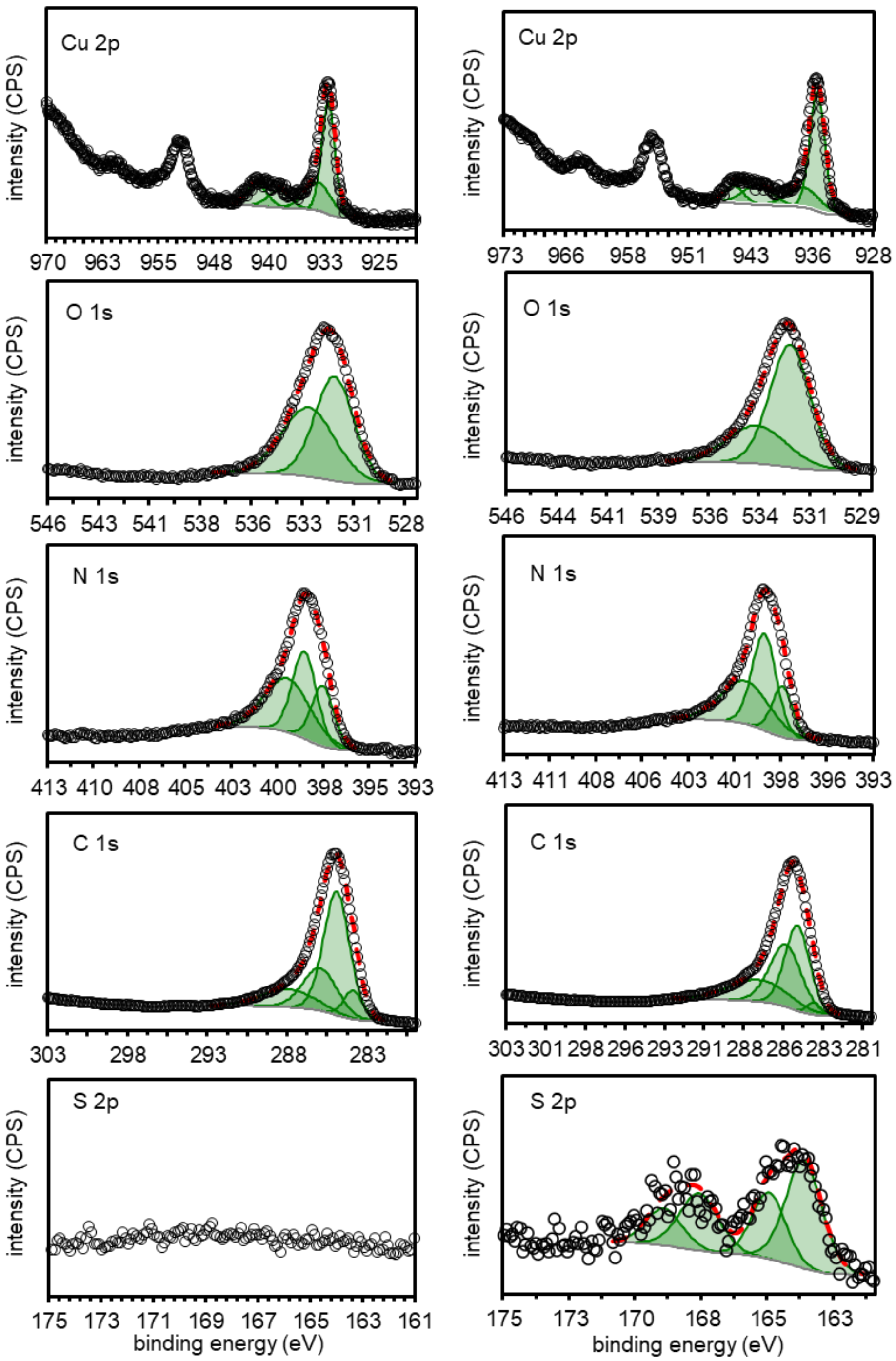

Figure S43. XPS analysis of the elemental regions of pristine CuPc-COF-DC-8 (left) and material after exposure to H_2S (right). Two sets of emission lines were observed in the region 160–175 eV.

XPS analysis after exposure to NH_3. Similar to CO, the elemental regions anticipated for NH_3 (N 1s) did not present a fully orthogonal region for characterizing the adsorbed probe gas. Instead, comparisons were drawn between the emission spectra of the elemental regions of the pristine material and after exposure to NH_3.

Fe-COF-DC-8 + NH_3

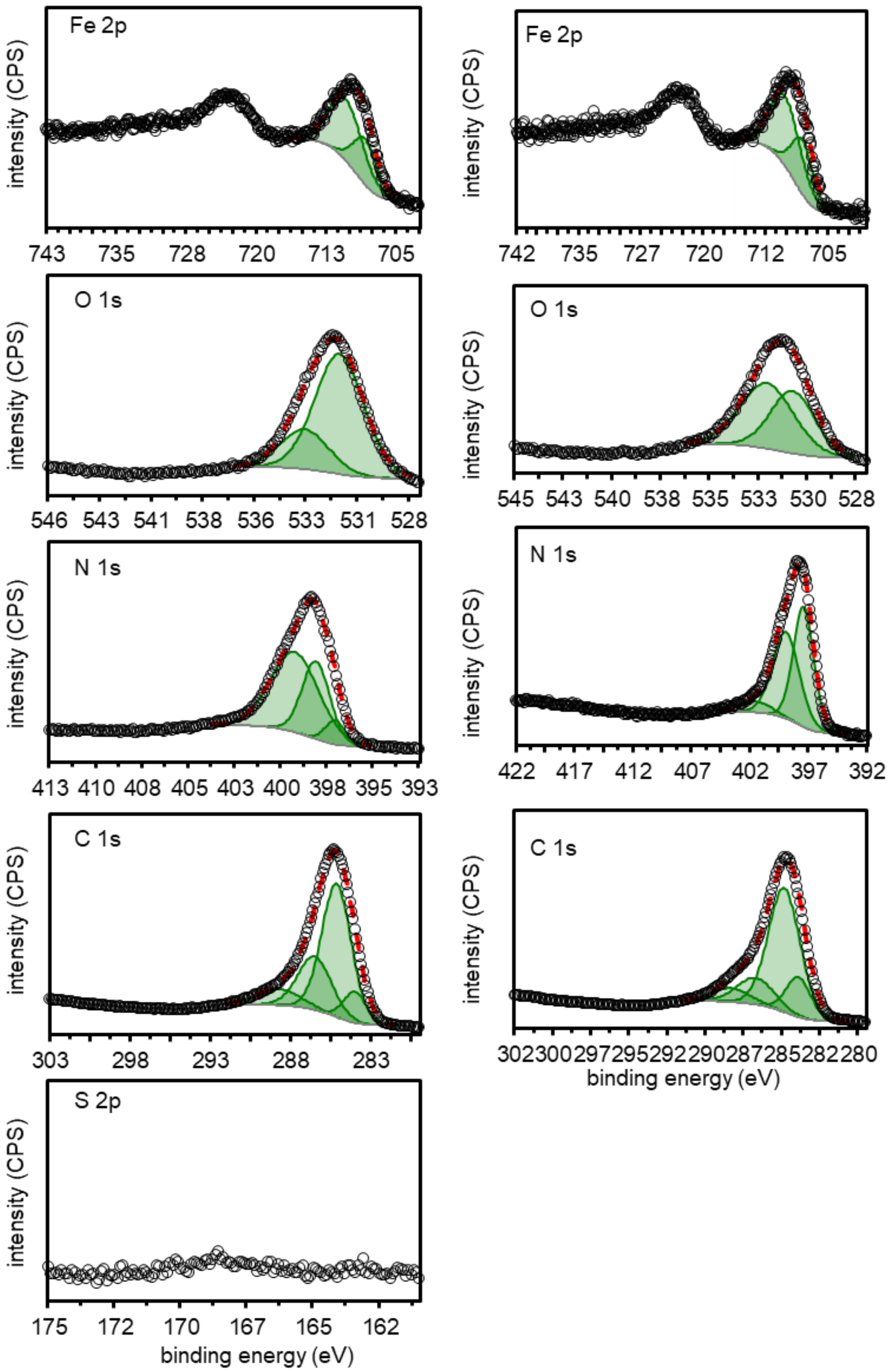

Figure S44. XPS analysis of the elemental regions of pristine FePc-COF-DC-8 (left) and material after exposure to NH_3 (right). We were not able to distinguish any new components in the N1s region corresponding to the adsorption of NH_3.

Co-COF-DC-8 + NH_3

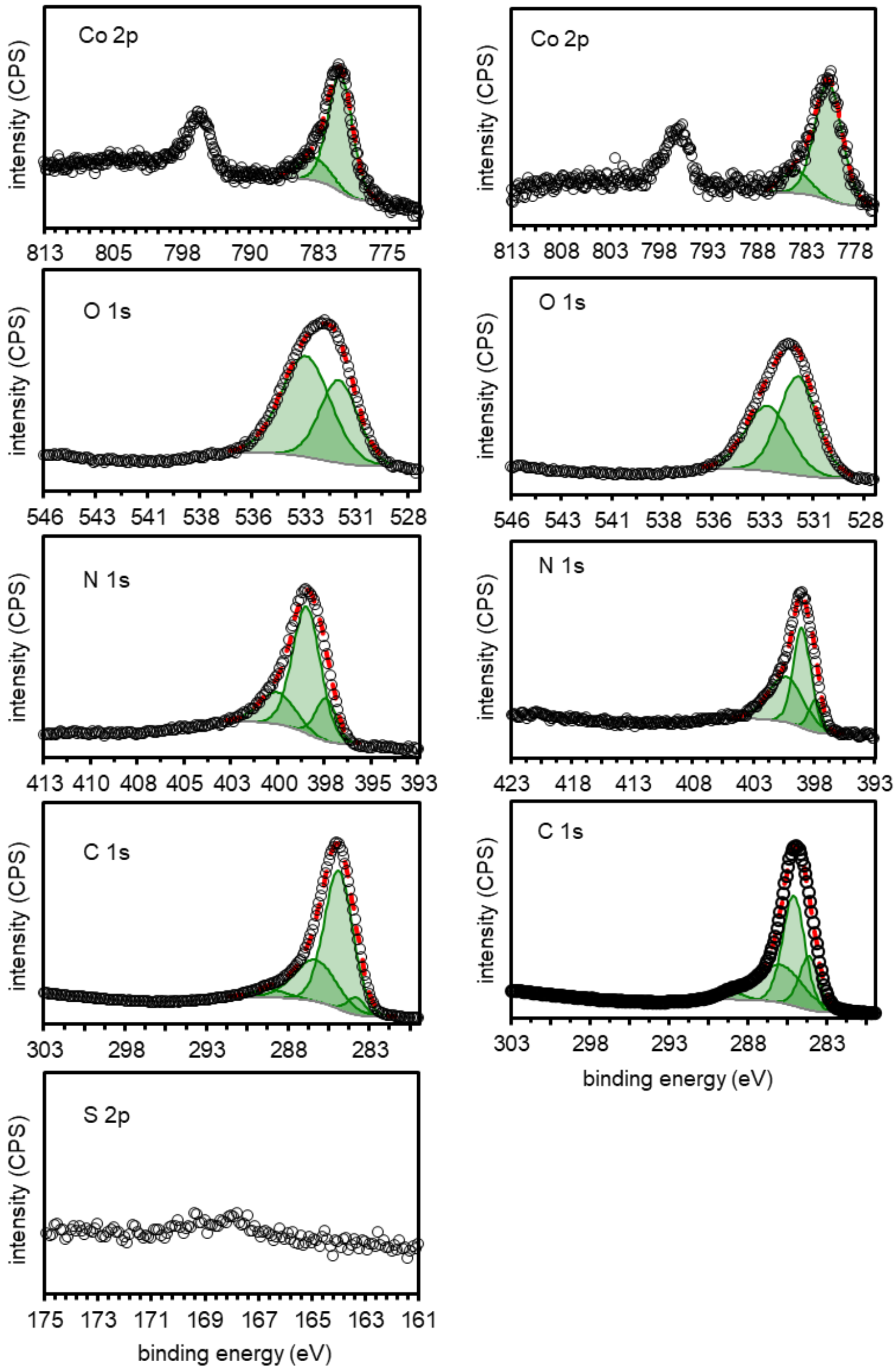

Figure S45. XPS analysis of the elemental regions of pristine CoPc-COF-DC-8 (left) and material after exposure to NH_3 (right). We were not able to distinguish any new components in the N1s region corresponding to the adsorption of NH_3. The Co $2p_{3/2}$ did not reveal any changes in oxidation state with adsorption of NH_3.

Ni-COF-DC-8 + NH_3

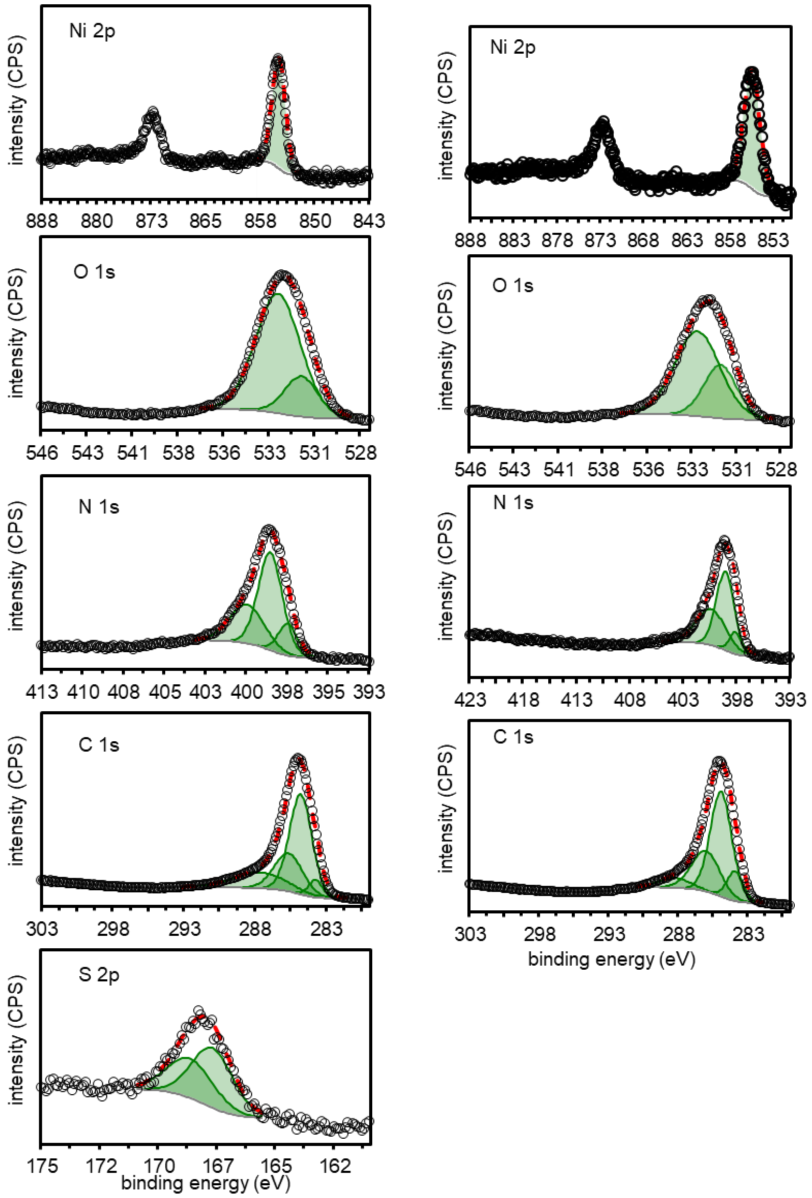

Figure S46. XPS analysis of the elemental regions of pristine NiPc-COF-DC-8 (left) and material after exposure to NH_3 (right). We observed a key difference in the O 1s region when comparing results to that of the pristine Ni-COF-DC-8 material. Specifically, the component at higher binding energies 533 eV, decreased relative to the lower energy component 532 eV. We cautiously attributed this removal of surface adsorbed water with exposure to NH_3, which we observed by DRIFTS spectroscopy. We were not able to distinguish any new components in the N1s region corresponding to the adsorption of NH_3. Specific differences in hydration level were difficult to quantify due to instrumental limitations in controlling the time needed for each sample that lead to different amounts of time subjected to the vacuum conditions of the XPS instrument (~10^{-7} torr).

Cu-COF-DC-8 + NH_3

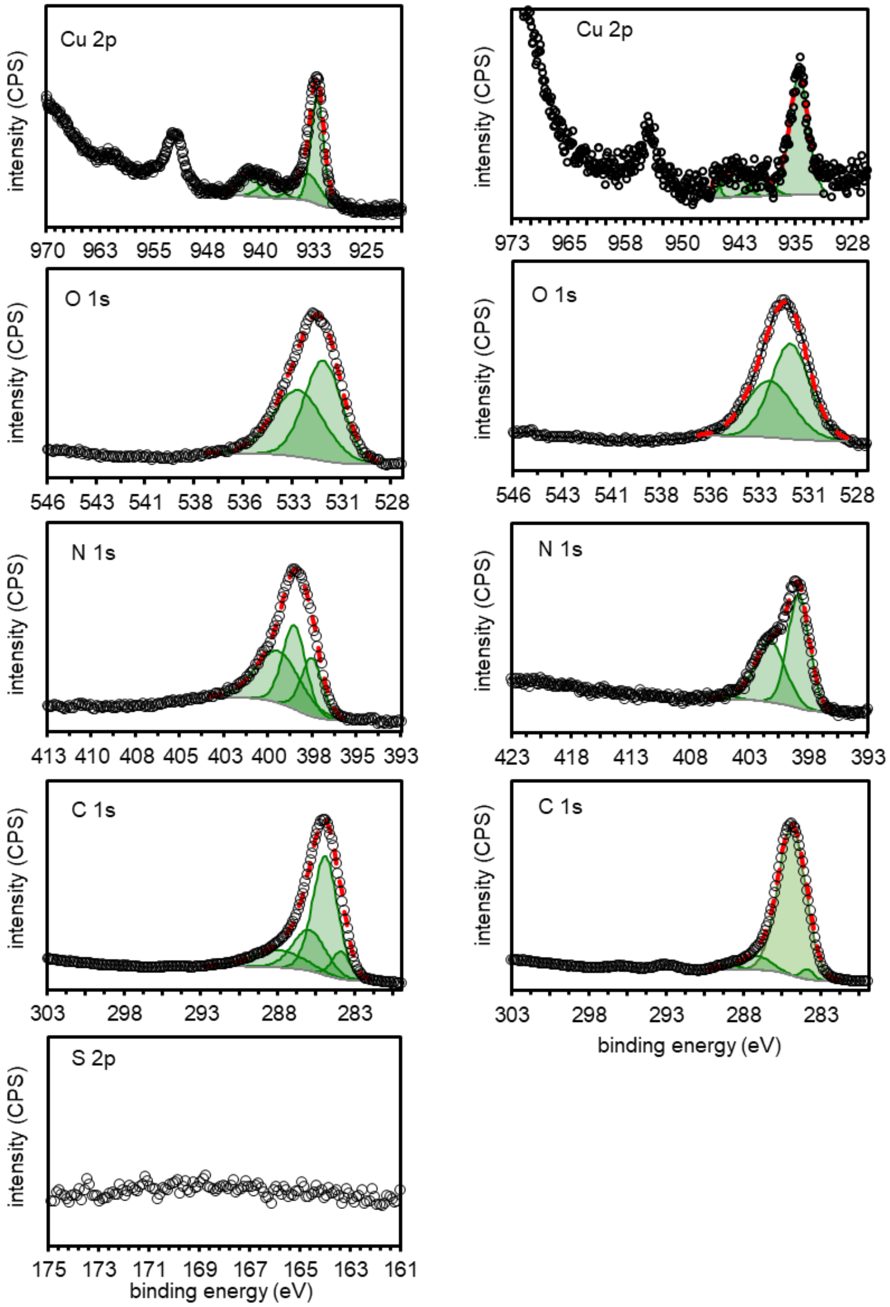

Figure S47. XPS analysis of the elemental regions of pristine CuPc-COF-DC-8 (left) and material after exposure to NH_3 (right). The N 1s and C 1s regions showed differences compared to the pristine material which suggested that NH_3 was possibly causing transformations within the framework. The N 1s region could be expected for a strong adsorbing NH_3 species. However, the C 1s region was interesting because it may indicate that edge functionality (altered by NH_3) was contributing a large portion of the observed photoelectrons.

EPR after gas exposure

After obtaining spectra of each pristine and degassed sample. Pristine samples were exposed to each analyte for 20 min before being degassed briefly (10 s) to remove any analyte in the headspace and reanalyzed under similar EPR experimental parameters (X-band, 4.2 K, etc.).

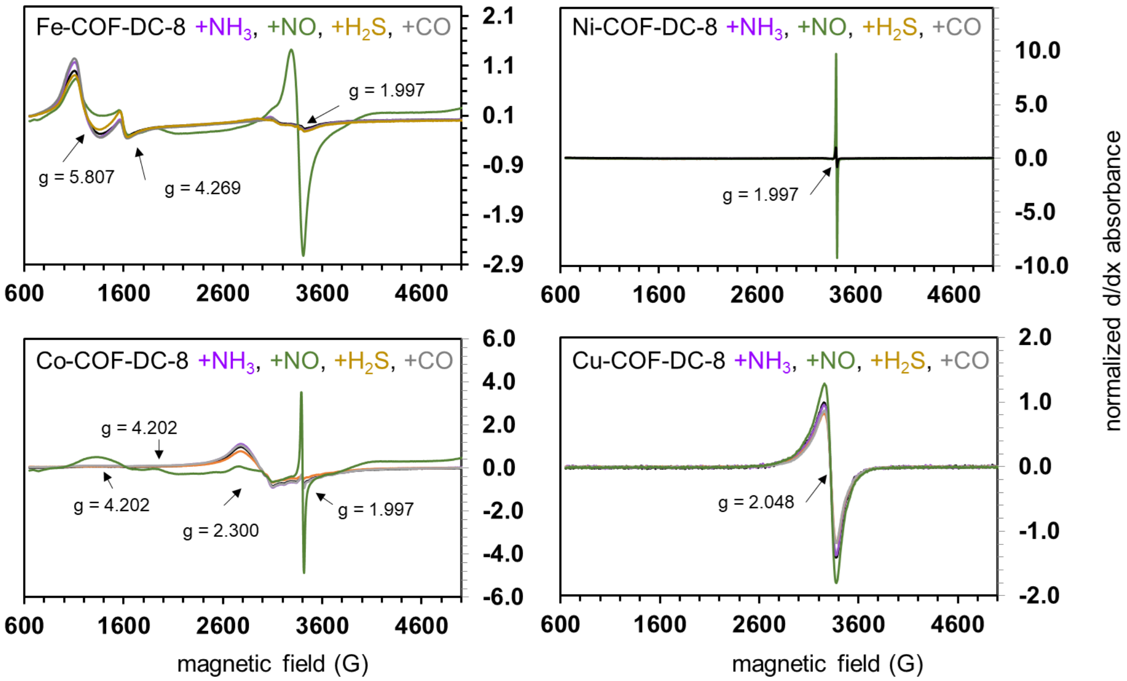

Figure S48. EPR spectra of the materials before and after exposure to analytes. Analytes are color-coded NH_3: purple, NO: green, H_2S orange, and CO: grey.

XIV. Methodology for PCA of the Sensor Array Response Data

We constructed a vector $\boldsymbol{x} \in \mathbb{R}^{3N}$ to represent each step response of the *N=4* [Fe, Co, Ni, Cu]-COF sensor array by concatenating (1) the initial slope, (2) the extremum, and (3) the area under the response curve (ie., $-\Delta G/G_0$ (%) vs. time) of each sensor. Note, each feature could be positive or negative. This gave a set of vectors $\{\boldsymbol{x}_i\}_{i=1}^{G}$ representing the responses of the sensor array to G=47 different gas exposure experiments, either 5, 10, 20, 40, or 80 ppm of NO, CO, H_2S, or NH_3 in dry N_2. Because the response vectors lie in a high-dimensional space ($\mathbb{R}^{3N=12}$), we resorted to principal component analysis (PCA), a method for unsupervised dimensionality reduction, to embed them into a 2D space for visualization.

Preparing the response data for PCA, we (1) transformed each response feature with (a) the Yeo-Johnson power transformation[15] followed by (b) z-score standardization, to endow the feature with a Gaussian-like distribution with mean zero and unit variance, then (2) constructed the data matrix $\boldsymbol{X} \in \mathbb{R}^{3N\times G}$ whose columns contain the transformed response vectors $\{\hat{\boldsymbol{x}}_i\}_{i=1}^{G}$.

We employ PCA to compress each response vector $\hat{\boldsymbol{x}}_i$ in a column of $\boldsymbol{X}$ into an encoding/latent vector $\mathbf{z}_i \in \mathbb{R}^2$ that approximates it. Particularly, PCA finds an orthogonal projection $\tilde{\boldsymbol{x}}_i = \boldsymbol{W}\boldsymbol{z}_i$ of each response vector $\hat{\boldsymbol{x}}_i$ by minimizing the reconstruction loss $\ell(\mathbf{W}, \mathbf{Z}) = ||\boldsymbol{X} - \boldsymbol{W}\boldsymbol{Z}||_F^2$ (F: Frobenius norm) under the constraint that $\mathbf{W} \in \mathbb{R}^{3N\times 2}$ has orthonormal columns. The two key outputs of PCA are (i) the encodings $\{\boldsymbol{z}_i\}_{i=1}^{G}$ of the response vectors contained in the columns of $\boldsymbol{Z} \in \mathbb{R}^{2\times G}$ and (ii) the matrix $\boldsymbol{W}$ whose two columns are the first two PCs of the data onto which we project the original (transformed) vectors $\{\hat{\boldsymbol{x}}_i\}_{i=1}^{G}$. Note, we can encode a new response vector $\boldsymbol{x}'$, after standardizing it to get $\hat{\boldsymbol{x}}'$, by projecting it onto the learned PCs, giving encoding $\boldsymbol{z}' = \boldsymbol{W}^{\boldsymbol{T}}\hat{\boldsymbol{x}}'$. Minimizing $\ell(\mathbf{W}, \mathbf{Z})$ under the constraint $\boldsymbol{W}^{\boldsymbol{T}}\boldsymbol{W} = \boldsymbol{I}$ is equivalent to choosing the orthonormal columns of $\mathbf{W}$ to align with the vectors onto which projections of the data exhibit maximal variance.[16, 17]

Finally, we applied PCA to the data matrix $\boldsymbol{X}$ using scikit-learn's implementation of PCA.[18]

The raw data and our Python code for both processing the data and conducting PCA are available at https://github.com/SimonEnsemble/cof-sensor-array-v2.

Since the sensor array response dataset contains a small number of examples (CO: 2, H_2S: 17, NH_3: 16, NO: 12, ∅: 2), overfitting is a concern when training a supervised machine learning model. To avoid overfitting, we (1) extracted only three intuitive, hand-crafted features from the

sensing trace as opposed to (a) learning features from the entire time series data e.g. via a recurrent or convolutional neural network[19-21] or (b) extracting numerous non-task-specific time series features[22] and (2) adopted a nearest-neighbor classifier as opposed to a parametric machine learning model, such a neural network, with many fitting parameters. Still, a *k*-nearest neighbors model with $k=1$ is prone to overfitting by describing an overly-complex (i.e., high variance) decision boundary.[23] However, we had no choice but to use $k=1$ because we possess only two examples of CO exposure (one allocated to the train set, the other to the test set). Our evaluation methodology—a test/train split of the examples according to concentration—would indicate overfitting by showing a poor accuracy on the test set. Instead, we found perfect accuracy on the test set, which serves as evidence that the model did not overfit. Notably, we avoided leaking subtle information about the test cases into the training set, too: instead of allocating each example to the test or train partition at random, we split the examples by *concentration* to prevent any two *replicate* experiments (associated with the same analyte concentration) from belonging to different partitions (one train, one test). If, instead, two replicate experiments did belong to different partitions, it would be easier for the model to predict a correct label on the one in the test set because the model would have encountered (and "memorized") a very similar response pattern (due to the same concentration) in the train set.

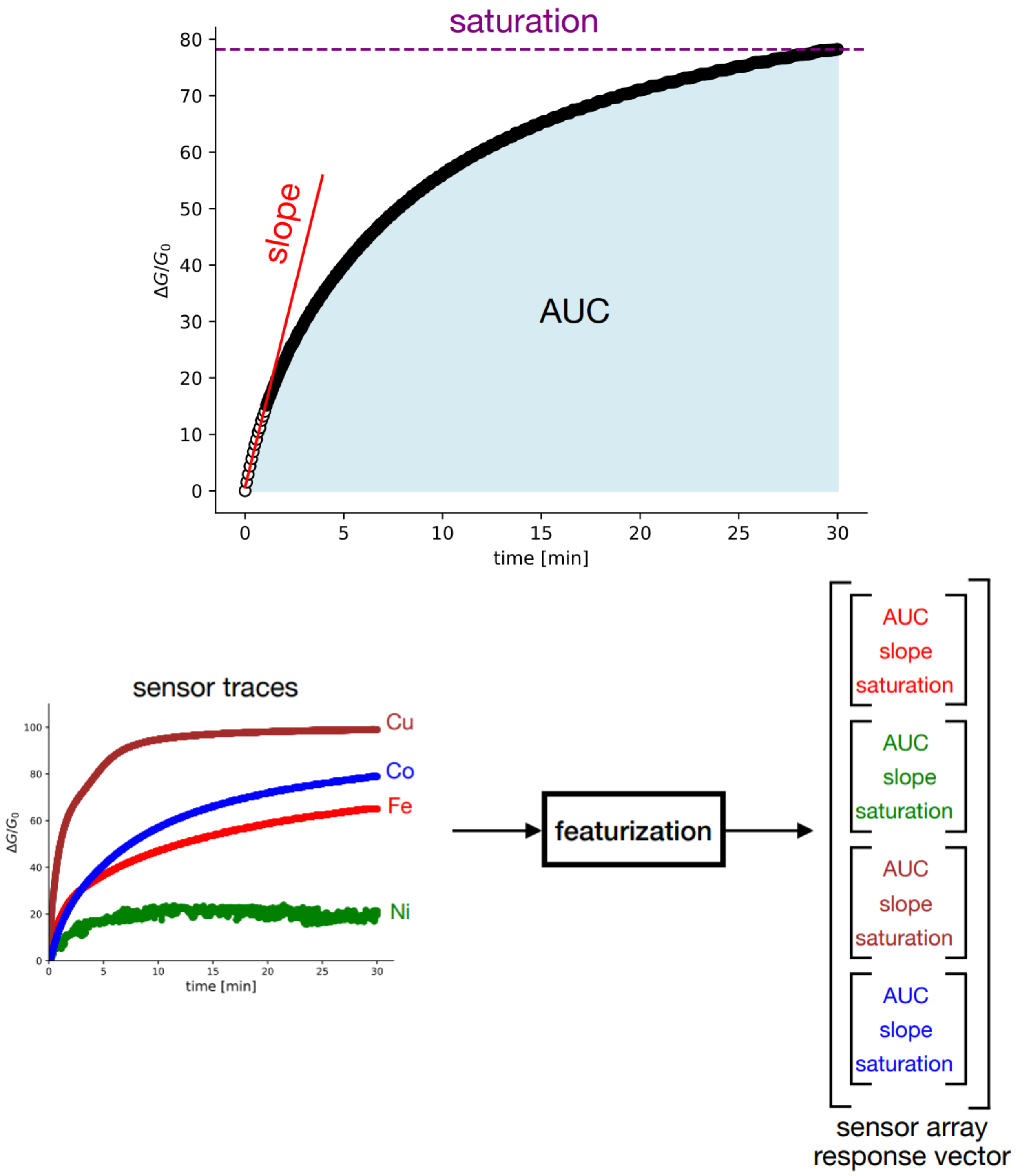

Figure S49. High-level workflow for our machine learning analysis of the response pattern of the sensor array.

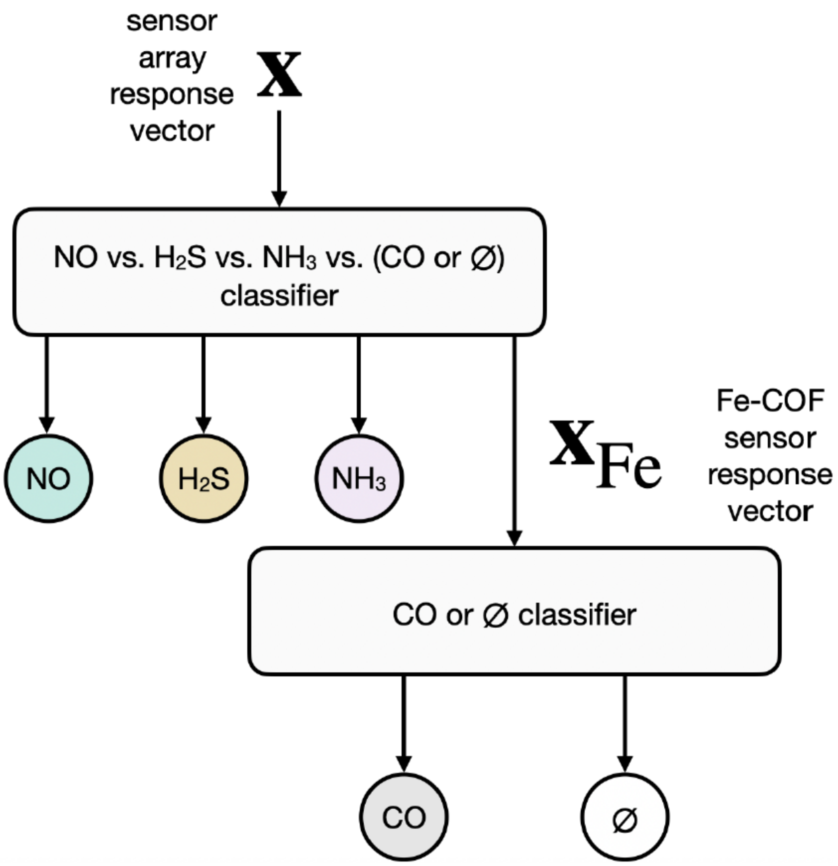

Figure S50. Decision tree schematic overviewing the two-step process employed to discriminate between the carrier gas, CO, H_2S, NH_3, and NO analytes and different concentrations of them based on the response of the M-COF-DC-8 (M=Fe, Co, Ni, and Cu) sensor array.

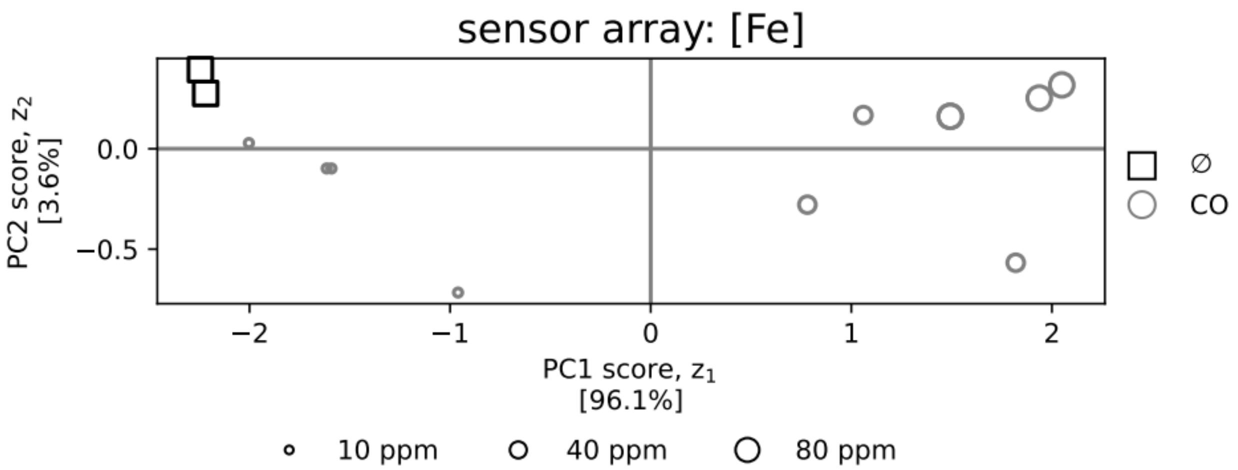

Figure S51. PCA to evaluate the ability to discriminate between the carrier gas and CO at different concentrations based on the response of the Fe-COF-DC-8 sensor.

analyte	train set	test set
∅	1	1
CO	1x 80 ppm	1x 80 ppm
H_2S	2x 5 ppm<br>4x 10 ppm	4x 20 ppm<br>3x 80 ppm
NH_3	3x 5 ppm<br>3x 10 ppm	4x 20 ppm<br>3x 80 ppm
NO	3x 10 ppm<br>3x 40 ppm	3x 20 ppm<br>3x 80 ppm

Figure S52. The train/test split of our labeled examples (each example: observation of the response of the sensor array to an exposure of an analyte) for our supervised machine learning (nearest neighbor) model.

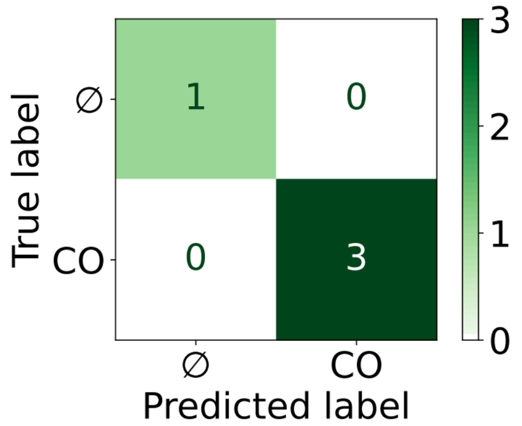

Figure S53. Confusion matrix over the test partition for the nearest neighbor classification of the analyte from the response of the Fe-COF-DC-8 sensor array towards carrier gas and CO. We employed the same procedure as for PCA to obtain the feature vector characterizing the response of the gas sensor array.

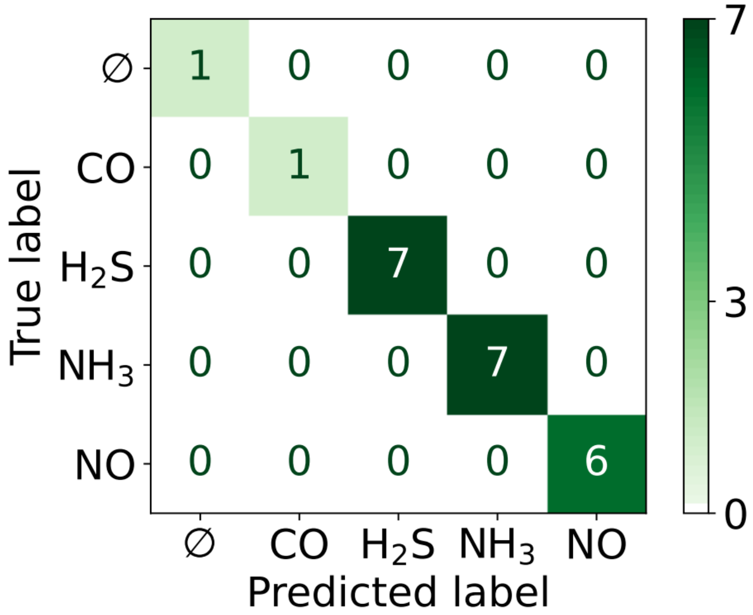

Figure S54. Confusion matrix over the test partition for the nearest neighbor classification of the analyte from the response of the [Fe, Co, Ni, Cu]-COF-DC-8 sensor array. We employed the same procedure as for PCA to obtain the feature vector characterizing the response of the gas sensor array.

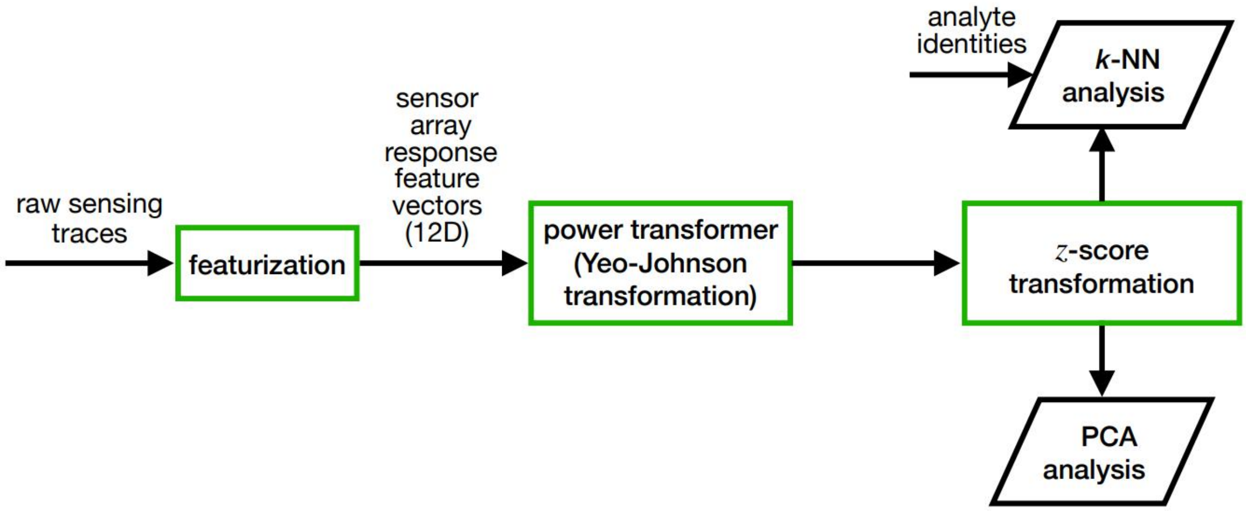

Figure S55. High-level workflow for our machine learning analysis of the response pattern of the sensor array.

Sensor Importance

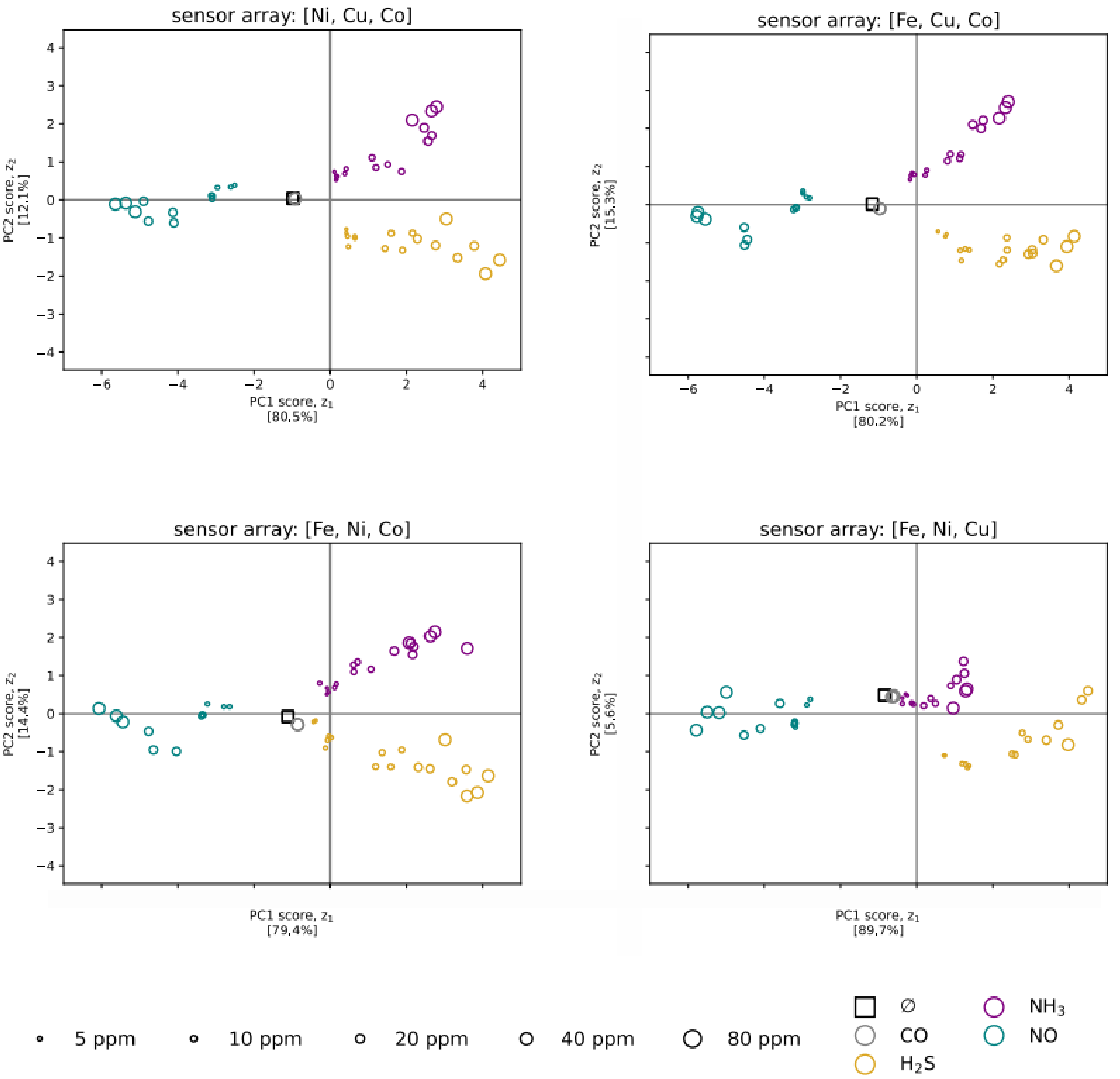

Figure S56. The PCA plot of the response vectors of the sensor array under dry conditions for $N = 3$ COF materials.

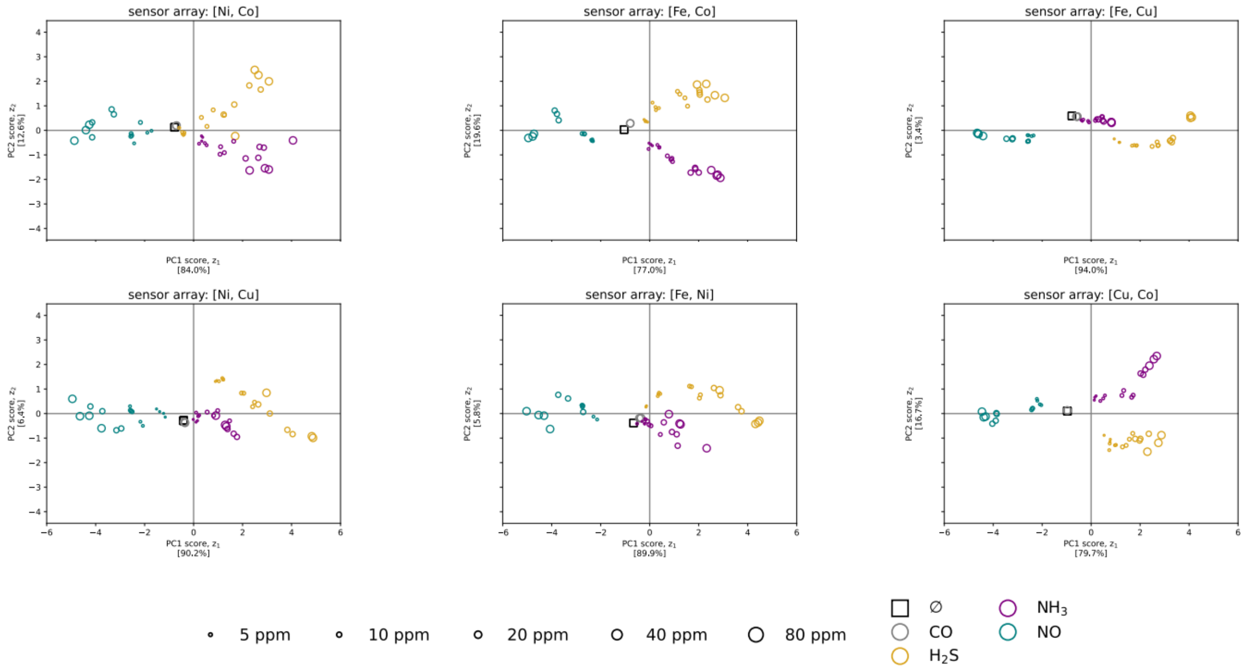

Figure S57. The PCA plot of the response vectors of the sensor array under dry conditions for $N = 2$ COF materials.

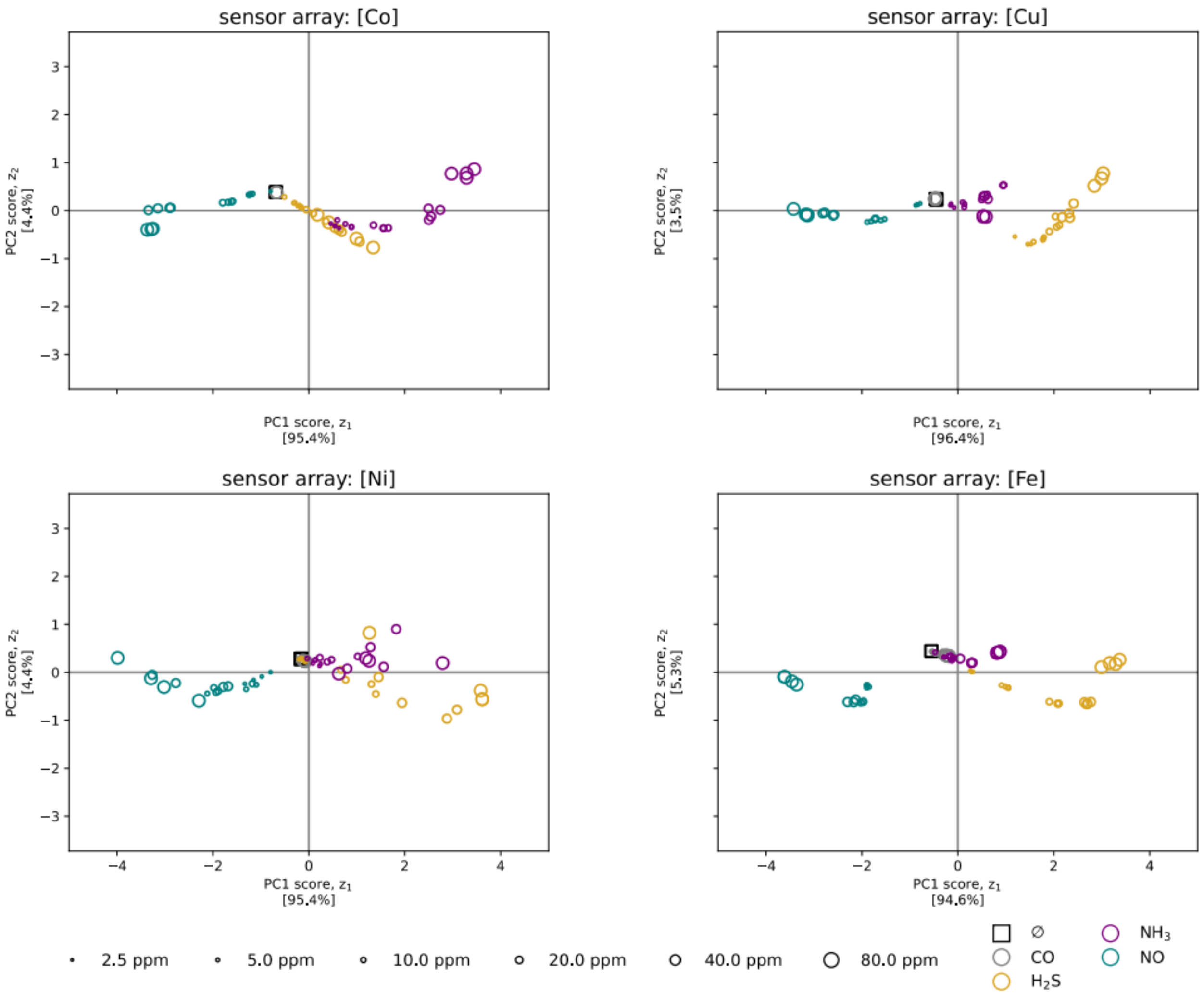

Figure S58. The PCA plot of the response vectors of the sensor array under dry conditions for $N = 1$ COF material.

XV. Computational Modeling of Host-Guest Interactions and Properties

Structural and electronic modeling of the M-COF-DC-8 materials was performed in *Material Studio 2019*. CASTEP was used with GGA-PBE methods to generate optimized unit cell geometries and to calculate high-symmetry K-points.[24, 25] The integrated density of states is displayed to the right of the corresponding band diagrams.[24] We studied potential unit cell symmetries having P4/mmm and I4/mmm symmetries to account for two possible spin orientations of metal-centered radicals in the COF. Specifically, Fe-COF-DC-8 (with potentially HS Fe^{2+} spin state), Co-COF-DC-8 (S=1/2 Co^{2+}), and Cu-COF-DC-8 (S=1/2, Cu^{2+}) were of interest due to the oxidation state of their metal constituents.

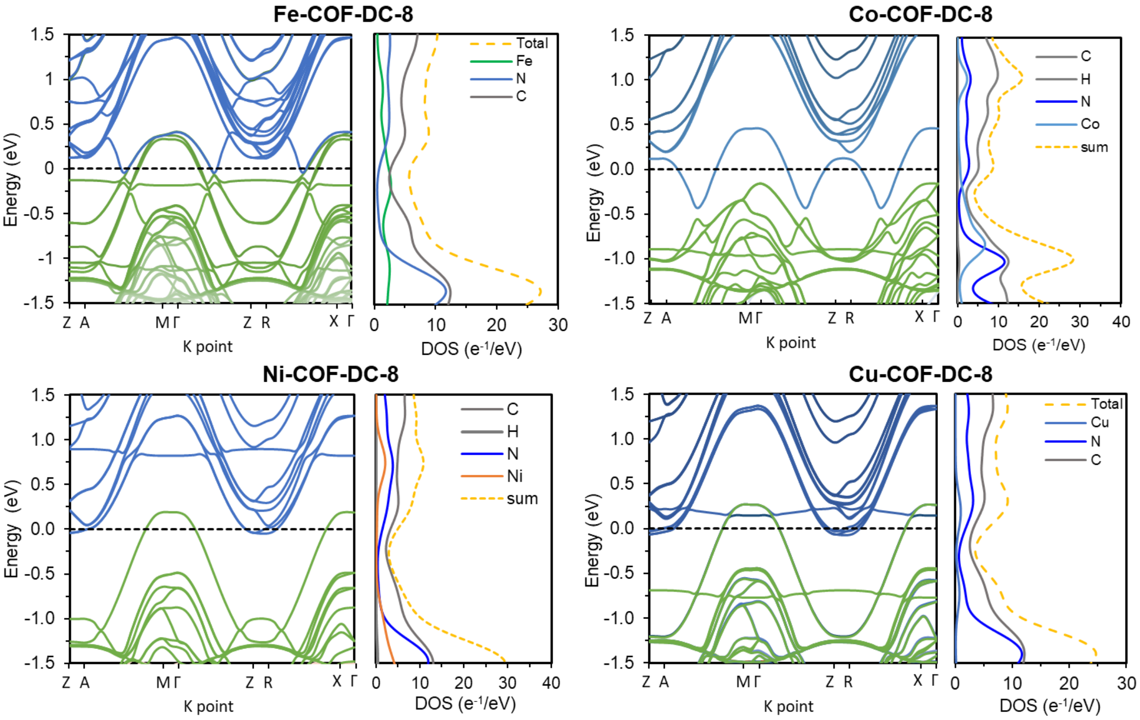

Figure S59. Computational models of the electronic band structure of MPc-based COFs exhibiting indirect band structures and zero bandgaps across all MPc analogs. The FePc and CuPc derivatives were modeled with ferromagnetic ordering. The NiPc derivative was modeled as low-spin S = 0.

The inclined stacking pattern of M-COF-DC-8 was structurally modeled using *Material Studio 2019*. CASTEP was used to geometrically optimize the eclipsed stacking pattern with GGA-PBE methods. The cut-off energy was set to 517.0 eV. The convergence tolerance was set at 1.0×10^{-5} eV/atom and the max force was set to 0.03 eV/A. Once the eclipsed structure was optimized, the α and β angles of the unit cell were set to 70° and the c axis was set to 3.711 Å. The Brillouin zones were sampled using a 1 × 1 × 8 k point mesh in the Monkhorst–Pack scheme.

Adsorption of Gases of the COF Basal Surface

The adsorption of gases to the M-COF-DC-8 frameworks is an important aspect of the sensing mechanism of the frameworks. Understanding gas adsorption phenomena, in particular favorable binding locations and geometries, adsorption energies, and influence of gas binding on the frameworks, could provide insight into the selectivity we observe in chemiresistive sensing experiments and provide additional insight into the reactivity of the COF exposed basal plane. We chose to examine the basal plane over other possible orientations because this surface was the most chemically distinct among the COFs and was most directly related to our proposed hypothesis related to selectivity imparted by the MPc unit. We modeled the four COFs in their pristine state as single layer, bi-layer, and tri-layer periodic structures capped with a 20 Å vacuum slab to simulate an exposed basal plane (**Figure S60**). To do this, we cleaved one layer in the (001) orientation of the P4/mmm unit cell and placed the cleaved layer in a periodic vacuum slab where the c direction was extended to 20 Å to ensure that layers and adsorbates were decoupled from the layer above and below. We refer to these structures by the abbreviation MPc-COF because they impose two important structural differences on the simulations compared to the M-COF-DC-8 compounds. First, the models we used for this study lacked structural defects such as stacking errors, omissions, and edge sites. Second, the models are monolayered, bilayered, or trilayered structures that are inherently electronically and chemically different from the experimentally obtained examples of MPc frameworks. These structures were optimized using the meta-generalized-gradient-approximation (mGGA) by Tao, Perdew, Staroverov, Scuseri (TPSS) modified with Grimme's dispersion correction (D3).[26, 27] Adsorption locations were identified using a Monte Carlo method in *Material Studio*. The most promising adsorption configurations were then optimized to a higher level of theory using the mGGA functional TPSS-D3 mentioned above.

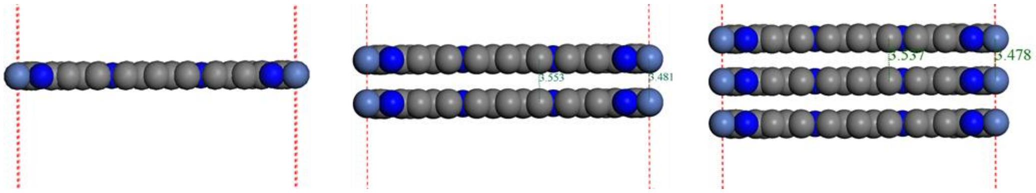

Figure S60. Monolayer, bilayer, and trilayer models of the COFs capped with vacuum slabs (10 nm) in the z-direction (c-axis) were used for the assessment of material properties after adsorbing gases. The multi-layered structures will be used in future directions.

We explored the adsorption properties of the analytes in our study (NO, CO, H_2S, and NH_3) on the monolayered materials modeled above. We also examined the adsorption of possible interferents (O_2 and H_2O). Adsorption energies were extracted using **Equation S6**.[3]

$$E_{ads} = E_{COF+gas} - (E_{COF} + E_{gas}) \quad \textbf{Eq. S6}$$

For the probes, the most exergonic binding location identified by simulation was the interaction of the probes with exposed metal sites (**Figure S61**). The adsorption energies between small gases and M-COF-DC-8 monolayers are reported in **Table S15**. This was expected from previous computational reports that detailed the high affinity of the metal centers for gas adsorption.[28] The adsorption enthalpies we calculated using this approach indicated that the metal center of the Fe-COF-DC-8 provided the most exergonic adsorption site among the MPc-COF derivatives and was the most capable of forming strong interactions with back binding adsorbates such as CO and NO. These binding energies were obtained for adsorption on a monolayer structure.

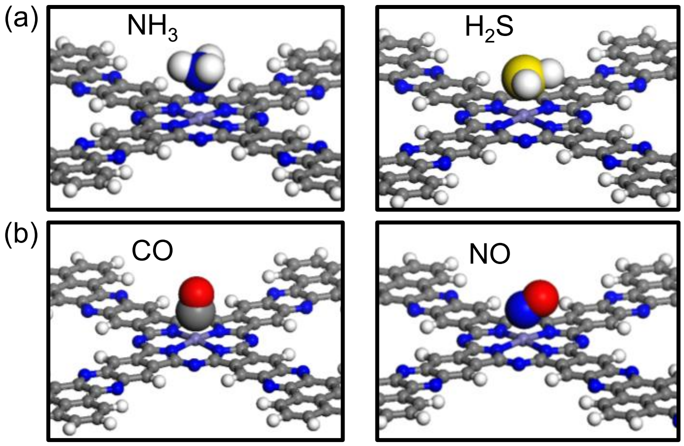

Figure S61. Ball and stick models of metal-centered host sites of M-COF-DC-8 with adsorbed analytes. Interactions feature (**a**) protic Lewis basic gases (i.e., NH_3 and H_2S), and (**b**) diatomic oxides (i.e., CO and NO).

E_{ads} (kcal/mol)	FePc-COF	CoPc-COF	NiPc-COF	CuPc-COF
CO	-91.2	-11.6	-3.3	-55.8
NO	-102.7	-80.4	-13.9	-32.1
O_2	-68.2	5.3	8.2	N. C.
NH_3	-106.0	-80.7	-15.4	-12.7
H_2S	-83.4	-63.2	-62.4	-59.4
H_2O	-86.1	-18.0	-61.5	-55.1

Table S15. Exergonicity of the adsorption process of small gases with MPc metal centers embedded in the basal plane of the MPc-COF monolayer model materials. These binding energies were obtained for adsorption on a monolayer structure. N.C.: No convergent structures could be found for these combinations.

After FePc, a general trend was observed with decreasing adsorption energies towards most analytes moving across the periodic table to CuPc-COF. This trend was consistent with previous computational studies that examine the interaction between MPc molecules and small gases. However, the magnitude of the exergonicity for specific strong-binding pairs seemed high and diverged from previous examples in the literature.[3] An important difference between the monolayer structures we examined here, and the majority of published work, is that most previous studies examine adsorption phenomena on single molecules whereas we examine adsorption on a periodic system. Having a periodic system and assigned symmetry artificially enforces structural rigidity in the *ab*-plane which limits deformation of the MPc units when interacting with strong

adsorbates. Such deformations are widely implicated in the chemistry of these molecular complexes (such as in heme-containing proteins).[29] Because these deformations that usually incur energetic penalties were subdued, we hypothesized that adsorption energies were increased relative to previous examples. A final observation we made from this initial adsorption study, was that analytes such as H_2O could energetically compete with the adsorption of targeted analytes such as CO in certain cases (NiPc-COF, CuPc-COF).

However, the pristine uncoordinated nature of the metal center in these initial studies was not what we anticipated as the true chemical state during sensing due to the numerous aqueous, and ambient air conditions the COFs were exposed to before integration into a chemiresistive device. We, therefore, assessed the exergonicity of the COF–probe systems as substitution reactions where a water molecule was first removed from the Lewis acid absorption site (LAS) of the MPc units before adsorption of the probe (see **Table S16**). This approach represented a more realistic assessment of the adsorption process and successfully supported the spectroscopic results where Fe-COF-DC-8 was selective for adsorption of CO and the responsible material feature was the metal identity.

E_{ads} (kcal/mol)	FePc-COF	CoPc-COF	NiPc-COF	CuPc-COF
CO	-5.0	6.4	58.2	-0.7
NO	-16.5	-62.4	47.6	23.0
O_2	17.9	23.3	69.7	N.C.
NH_3	-19.9	-62.7	46.0	42.4
H_2S	2.8	-45.1	-0.9	-4.3
H_2O	0.0	0.0	0.0	0.0

Table S16. Exergonicity of the adsorption process of small gases as substitution reactions removing an aqua ligand from the MPc adsorption site. N.C. no convergent structures could be found.

To test our hypothesis that the conformational restriction in our model, caused by the periodicity of the adsorbate, we independently calculated the adsorption energy of small gases on MPc molecules using Gaussian09.[30] Although direct comparisons between the E_{ads} values using TPSS-D3 and wb97X-D3 would be inappropriate, trends in the magnitude of resulting E_{ads} values supported our theory that the structural deformations allowed by single molecules of MPc may account for the exaggerated values observed for adsorption at monolayered structures. These

adsorption location studies conducted on MPc molecules demonstrate stronger gas adsorption energies for both Fe- and Co-derivatives compared to Ni- and Cu-derivatives (**Table S17**). This modelled finding aligns with spectroscopic evidence for the Fe- and Co-based materials stronger interaction with NO and CO when compared with the Ni- and Cu-based extended solids The Gaussian09 calculations also supported the trend of the strongest MPc-based surface adsorption site being FePc and the weakest being NiPc (**Table S17**).

E_{ads} (kcal/mol)	FePc	CoPc	NiPc	CuPc
NO	-27.1	-21.5	-4.8	-5.0
CO	-28.3	-25.5	-4.9	-5.3
O_2	-18.8	-19.9	-4.0	-4.1
NH_3	-28.0	-37.0	-9.1	-15.8

Table S17. Exergonicity of the probe gases binding to MPc molecules instead of periodic monolayers. These values were obtained from structures optimized in Gaussian09 using wB97X (6-31G) with an empirical dispersion correction (Grimme's D3).

XVI. References

(1) Hu, J.; Zhang, D.; Harris, F. W. Ruthenium(III) chloride catalyzed oxidation of pyrene and 2,7-disubstitued pyrenes: an efficient, one-step synthesis of pyrene-4,5-diones and pyrene-4,5,9,10-tetraones. *J. Org. Chem.* **2005**, *70* (2), 707-708.
(2) Shao, J.; Chang, J.; Chi, C. Linear and star-shaped pyrazine-containing acene dicarboximides with high electron-affinity. *Org. Biomol. Chem.* **2012**, *10* (35), 7045-7052.
(3) Jia, H.; Yao, Y.; Zhao, J.; Gao, Y.; Luo, Z.; Du, P. A novel two-dimensional nickel phthalocyanine-based metal–organic framework for highly efficient water oxidation catalysis. *Journal of Materials Chemistry A* **2018**, *6* (3), 1188-1195.
(4) Cheng, Z.; Cui, N.; Zhang, H.; Zhu, L.; Xia, D. Synthesis and Dimerization Behavior of Five Metallophthalocyanines in Different Solvents. *Adv. Mater. Sci. Eng.* **2014**, *2014*, 1-5.
(5) Meng, Z.; Stolz, R. M.; Mirica, K. A. Two-Dimensional Chemiresistive Covalent Organic Framework with High Intrinsic Conductivity. *J. Am. Chem. Soc.* **2019**, *141* (30), 11929-11937.
(6) Zhang, M. D.; Si, D. H.; Yi, J. D.; Zhao, S. S.; Huang, Y. B.; Cao, R. Conductive Phthalocyanine-Based Covalent Organic Framework for Highly Efficient Electroreduction of Carbon Dioxide. *Small* **2020**, *16* (52), e2005254.
(7) Ammu, S.; Dua, V.; Agnihotra, S. R.; Surwade, S. P.; Phulgirkar, A.; Patel, S.; Manohar, S. K. Flexible, all-organic chemiresistor for detecting chemically aggressive vapors. *J. Am. Chem. Soc.* **2012**, *134* (10), 4553-4556.
(8) Meng, Z.; Stolz, R. M.; De Moraes, L. S.; Jones, C. G.; Eagleton, A. M.; Nelson, H. M.; Mirica, K. A. Gas-Induced Electrical and Magnetic Modulation of Two-Dimensional Conductive Metal-Organic Framework. *Angew. Chem. Int. Ed. Engl.* **2024**, *63* (24), e202404290.
(9) Chan, J. Y. M.; Shehayeb, E. O.; Pennington, D. L.; Hendon, C. H.; Mirica, K. A. Molecular Engineering of a Conductive Metal-Organic Framework for Ultrasensitive, Rapid, Selective, and Reversible Sensing of Nitric Oxide. *J. Am. Chem. Soc.* **2025**, *147* (32), 29003-29012.
(10) England, C.; Corcoran, W. H. Kinetics and Mechanisms of the Gas-Phase Reaction of Water Vapor and Nitrogen Dioxide. *Ind. Eng. Chem., Fundam.* **2002**, *13* (4), 373-384.
(11) England, C. C., William H. The Rate and Mechanism of the Air Oxidation of Parts-per-Million Concentrations of Nitric Oxide in the Presence of Water Vapor. *Ind. Eng. Chem., Fundam.* **1975**, *14* (1).
(12) Campbell, M. G.; Liu, S. F.; Swager, T. M.; Dinca, M. Chemiresistive Sensor Arrays from Conductive 2D Metal-Organic Frameworks. *J. Am. Chem. Soc.* **2015**, *137* (43), 13780-13783.
(13) Logan, J. A. Nitrogen oxides in the troposphere: Global and regional budgets. *J. Geophys. Res.* **1983**, *88* (C15).
(14) Sjövall, H.; Blint, R. J.; Olsson, L. Detailed Kinetic Modeling of NH3 and H2O Adsorption, and NH3 Oxidation over Cu-ZSM-5. *J. Phys. Chem. C* **2009**, *113* (4), 1393-1405.
(15) Yeo, I. K.; Johnson, R. A. A new family of power transformations to improve normality or symmetry. *Biometrika* **2000**, *87* (4), 954-959.
(16) Bishop, C. M. *Pattern Recognition and Machine Learning*; Springer, 2006. DOI: 10.1117/1.2819119.
(17) Murphy, K. P. *Probabilistic machine learning: an introduction.*; MIT Press, 2022.
(18) Pedregosa, F.; Varoquaux, G.; Gramfort, A.; Michel, V.; Thirion, B.; Grisel, O.; Blondel, M.; Prettenhofer, P.; Weiss, R.; Dubourg, V.; Vanderplas, J.; Passos, A.; Cournapeau, D.; Brucher, M.; Perrot, M.; Duchesnay, É. Scikit-learn: Machine Learning in Python. *Journal of Machine Learning Research* **2011**, *12*, 2825-2830.
(19) Pan, X.; Zhang, H.; Ye, W.; Bermak, A.; Zhao, X. A Fast and Robust Gas Recognition Algorithm Based on Hybrid Convolutional and Recurrent Neural Network. *IEEE Access* **2019**, *7*, 100954-100963.

(20) Zhang, W.; Wang, L.; Chen, J.; Xiao, W.; Bi, X. A Novel Gas Recognition and Concentration Detection Algorithm for Artificial Olfaction. *IEEE Trans. Instrum. Meas.* **2021**, *70*, 1-14.
(21) Fukuyama, K.; Matsui, K.; Omatsu, S.; Rivas, A.; Corchado, J. M. Feature Extraction and Classification of Odor Using Attention Based Neural Network. Cham, 2020; Springer International Publishing: pp 142-149.
(22) Christ, M.; Braun, N.; Neuffer, J.; Kempa-Liehr, A. W. Time Series FeatuRe Extraction on basis of Scalable Hypothesis tests (tsfresh – A Python package). *Neurocomputing* **2018**, *307*, 72-77.
(23) James, G. W., D.; Hastie, T.; Tibshirani, R.; Taylor, J. *An introduction to statistical learning: Python Edition*; 2023.
(24) Clark, S. J.; Segall, M. D.; Pickard, C. J.; Hasnip, P. J.; Probert, M. I. J.; Refson, K.; Payne, M. C. First principles methods using CASTEP. *Z. Kristallogr. Cryst. Mater.* **2005**, *220* (5-6), 567-570.
(25) Perdew, J. P.; Burke, K.; Ernzerhof, M. Generalized Gradient Approximation Made Simple. *Phys. Rev. Lett.* **1996**, *77* (18), 3865-3868.
(26) Grimme, S.; Antony, J.; Ehrlich, S.; Krieg, H. A consistent and accurate ab initio parametrization of density functional dispersion correction (DFT-D) for the 94 elements H-Pu. *J. Chem. Phys.* **2010**, *132* (15), 154104.
(27) Tao, J.; Perdew, J. P.; Staroverov, V. N.; Scuseria, G. E. Climbing the density functional ladder: nonempirical meta-generalized gradient approximation designed for molecules and solids. *Phys. Rev. Lett.* **2003**, *91* (14), 146401.
(28) Rana, M. K.; Sinha, M.; Panda, S. Gas sensing behavior of metal-phthalocyanines: Effects of electronic structure on sensitivity. *Chem. Phys.* **2018**, *513*, 23-34.
(29) Reyes, Y. I. A.; Franco, F. C. DFT study on the effect of proximal residues on the Mycobacterium tuberculosis catalase-peroxidase (katG) heme compound I intermediate and its bonding interaction with isoniazid. *Phys. Chem. Chem. Phys.* **2019**, *21* (30), 16515-16525.
(30) *Gaussian 09, Revision A.02*; 2016.
